# Multicomponent Solids of Niflumic and Mefenamic Acids Based on Acid-Pyridine Synthon

**DOI:** 10.3389/fchem.2022.729608

**Published:** 2022-03-31

**Authors:** Vineet Kumar, Pramod Kumar Goswami, Balendra, Shailabh Tewari, Arunachalam Ramanan

**Affiliations:** ^1^ Department of Chemistry, Indian Institute of Technology Delhi, New Delhi, India; ^2^ Department of Chemistry, Sri Venkateswara College, University of Delhi, New Delhi, India

**Keywords:** cocrystallization, acid-pyridine synthon, intermolecular interactions, ΔpKa rule, hirshfeld surface analysis

## Abstract

The present study discusses comparative structural features of fourteen multicomponent solids of two non-steroidal anti-inflammatory drugs, Niflumic and Mefenamic acids, with amine and pyridine-based coformers. All the solids were structurally characterized through PXRD, SCXRD, DSC, and the monophasic nature of some of the solids was established through Rietveld refinement. The solid forms include salt, cocrystal, hydrate, and solvate. Except for two, all the solids reported here showed relatively higher solubility compared to the acids. The difference in p*K*a and similarity in structural features of both the molecules enabled us to study the effect of Δp*K*a on crystallization outcome systematically. The structures of all the solids are described through acid-pyridine synthon perspective.

## Introduction

**SCHEME 1:**
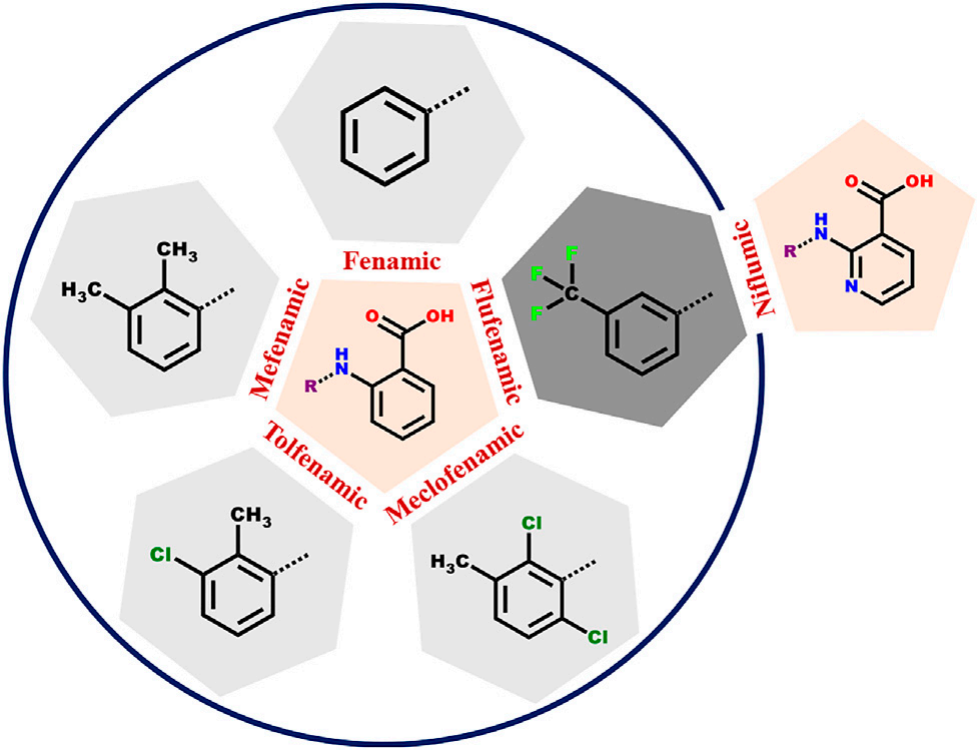
The anthranilic acid derivatives (fenamates) surveyed in this study. The five NSAIDs within the circle have a common anthranilic acid moiety with different substitutions. In case of Flufenamic and Niflumic acids, the difference is the presence of nitrogen instead of carbon in the anthranilic acid ring.

**SCHEME 2:**
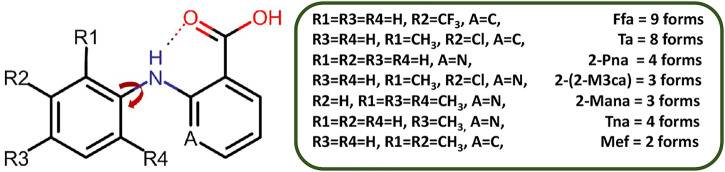
Polymorphic forms of fenamate derivatives. The substitutions on the phenyl ring are critical in governing the relative orientation of the two aromatic rings which in turn impacts the number of polymorphic forms as well as how it interacts with a coformer under given conditions. Here, Ffa = Flufenamic Acid, Ta = Tolfenamic Acid, 2-Pna = 2-(phenylamino)nicotinic acid, 2-(2-M3ca) = 2-(2-Methyl-3-chloroanilino)nicotinic acid, 2-Mana = 2-(Mesitylamino)nicotinic acid, Tna = 2-(phynylamino)nicotinic acid, and Mef = Mefenamic Acid.

**SCHEME 3:**
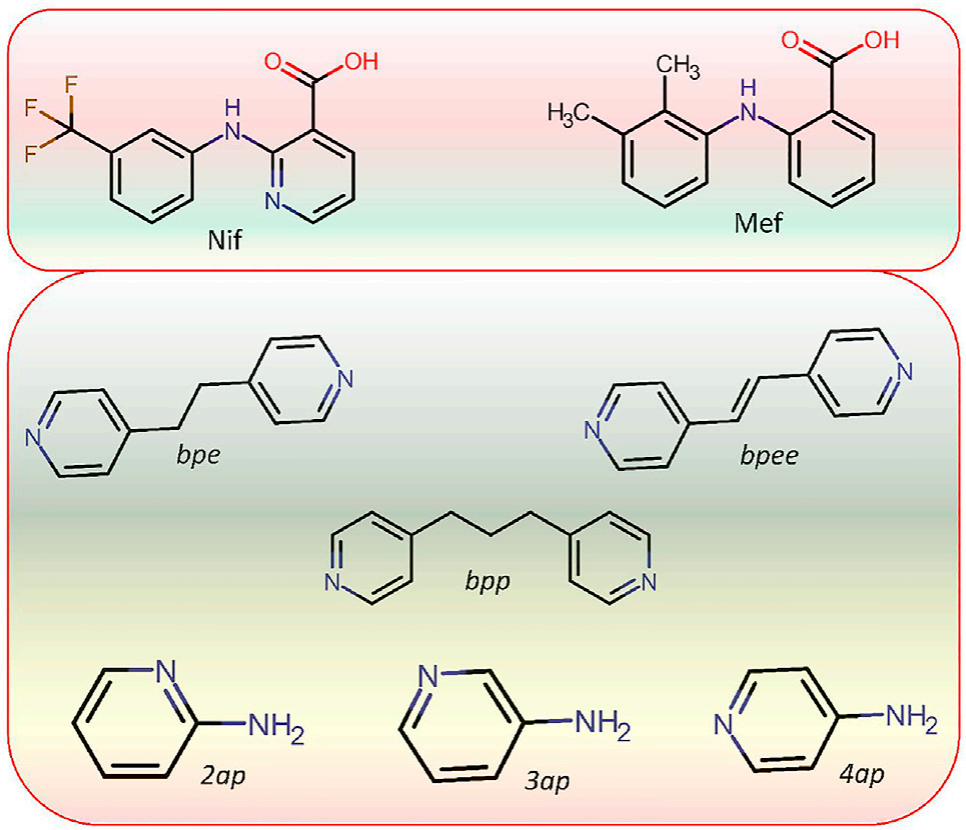
Molecular structure of APIs Niflumic (Nif) and Mefenamic (Mef) acid as well as coformers (*bpe, bpee, bpp,2ap,3ap,* and *4ap*) used in this study.

Niflumic acid (Nif) or 2-{[3-(Trifluromethyl)phenyl]amino}nicotinic acid, and Mefenamic acid (Mef) or 2-(2,3-dimethylphenyl) aminobenzoic acid, are non-steroidal anti-inflammatory drugs NSAIDs (Uyemura et al., 1997; Sturkenboom, 2005; Khansari and Halliwell, 2009). These NSAIDs are among the most commonly used pharmaceutical molecules, as analgesic, anti-inflammatory, and antipyretic agents (Dionne and Berthold, 2001; Garg and Azim, 2021). Nif and Mef are used to treat various diseases: Nif is used in rheumatoid arthritis, arthrosis, and joint diseases (Sydnes, 1973), while Mef is prescribed in dental pain, postoperative surgery, premenstrual syndrome, and headache (Bonnar and Sheppard, 1996). Mef has also shown anti-cancer activity (colon and liver cancer) and therapeutic effect in Alzheimer’s disease. Both Nif and Mef (along with Meclofenamic and Tolfenamic acid) belong to a class of NSAIDs called fenamates which are derivatives of anthranilic acid (Scheme 1). Fenamates, generally show poor solubility and high permeability and are classified as Class II drugs as per BCS (Biopharmaceutics Classification System) (SeethaLekshmi and Guru Row, 2012; Bodnár et al., 2017). Radacsi et al. used different crystallization techniques: microwave-assisted evaporation, electrospray, and atmospheric pressure cold plasma to improve the bioavailability of Nif (Radacsi et al., 2012a; Radacsi et al., 2012b; Radacsi et al., 2013). Szunyoghet al. tried nanonization of Niflumic acid by co-grinding to improve dissolution rate (Szunyogh et al., 2013). Wittering et al. employed cocrystallization to prevent polymorphism in fenamates (Wittering et al., 2015). Recently, Bhattacharya et al. improved solubility of fenamates by formation of drug-drug multicomponent solids with another drug trimethoprim (Bhattacharya et al., 2020). Moreover, the extensive use of these drugs regularly worldwide led to their presence in wastewater at higher concentrations than the predicted no effect concentration (Margot et al., 2015; Mila et al., 2019), and removing them can be a challenging task (Greenstein et al., 2018). Based on our earlier experience, robust acid-pyridine synthon can be utilized to tune the solubility of these molecules (Kumar et al., 2018; Goswami et al., 2020). In the first series, we employed 1,2-bis(4-pyridyl)ethane (*bpe*); 1,2-bis(4-pyridyl)ethylene (*bpee*), and 1,3-di (4-pyridyl)propane (*bpp*) with an objective to systematically vary the spacing and flexibility of 4,4-bipyridyl (*4,4-bpy*), which was earlier used by Wittering et al. as bipyridine are extensively used for the formation of robust material (polymers and membranes), which can further help to remove contaminants from wastewater. In the second series, the three aminopyridines, 2-aminopyridine (*2ap*), 3-aminopyridine (*3ap*), and 4-aminopyridine (*4ap*), were used as coformers to understand the structural chemistry and improve the solubility. Although the amino pyridines do not belong to the GRAS category and out of three *2ap*, (LD50 = 200 mg/kg in case of rat when used orally) (Shimizu et al., 2000), *3ap* (LD50 = 178 mg/kg in case of quail when used orally) (Shimizu et al., 2000) and *4ap* (LD50 = 20 mg/kg in case of rat when used orally) (Schafer, 1973) only the latter is well studied for medicinal use. These commonly used lab chemicals make attractive coformers due to their easy and reliable weak bond formation with a vast range of molecules. A CSD search of Nif showed overall thirty-nine hits, out of which twenty were organic solids. The other NSAID, Mef, showed one hundred and five solids, of which fifty-four were organic solids, of those only 32 and 13 are multicomponent solids, respectively. Fenamates are known to be polymorphic as a consequence of free rotation between the two rings, thus allowing them to have more than one crystal structure (as depicted in Scheme 2); exceptions include a few such as Nif and meclofenamic acid (Delaney et al., 2014; López-Mejías and Matzger, 2015). López-Mejías et al. discovered six new polymorphs for flufenamic acid; the compound is the second most polymorphic molecule (nine polymorphs) after 5-methyl-2-[(2-nitrophenyl)-amino]thiophene-3-carbonitrile or ROY (thirteen polymorphs) (López-Mejías et al., 2012). Uzoh et al. compared crystal energy landscapes of fenamates and showed that conformational flexibility between the two phenyl rings is responsible for this behavior (Uzoh et al., 2012). Though multicomponent solids of Mef are known with *bpe, bpee, bpp* and *4ap* (Nechipadappu and Trivedi, 2017; Zheng et al., 2018), the literature still lacks a comparative study of all these structures and their solubility. A careful analysis of the six selected anthranilic acids with N-based coformers reported in CSD (Supplementary Tables S1, S2) showed acid-pyridine (or N-based co-former) synthon drove the formation of the majority of the multicomponent solids. The table also highlights the need to explore the structural landscape of an acid molecule with a series of structurally related base coformers. Since acid-base interaction is a major driving force, it would be possible to rationalize the composition (A_2_B, AB or AB_2_) occurring at the microlevel and how further supramolecular aggregation to a cocrystal, salt or its solvate is facilitated through the functional groups at the periphery of these aggregates. In Scheme 3, we have given the molecular structure of APIs and coformers used in this study. We employed different crystallization techniques and solvent variations to investigate the structural landscape of the system, acid (Nif or Mef)-N-pyridine based conformer-solvent. In Table 1, we have provided the reaction condition for the isolation of the solids reported here.

**TABLE 1:** Crystallization method used for the synthesis of Nif and Mef based multicomponent solids in the present study and their physical properties. Solids **5**, **6** and **11** could not be isolated as pure phases.

Composition in the solid	Method and solvent of crystallization	Color and morphology	m. p. (°C) (from DSC)
Nif	Used as received	Greenish	203
Mef	Used as received	Colorless	230
(Nif)_2_·(bpe) (1)	Neat grinding, methanol assisted grinding, methanol	Rod, colorless	177
(Nif)_2_·(bpee) (2)	Neat grinding, methanol assisted grinding, methanol	Block, green	179
(Nif)_2_·(bpee).1,4-dioxane (2a)	1,4- dioxane assisted grinding, 1,4- dioxane	Rod, green	178
(Nif)_2_·(bpp) (3)	Neat grinding, methanol assisted grinding, methanol	Rod, colorless	92
(Nif)^−^·(2apH)^+^(4)	Neat grinding, methanol assisted grinding, methanol	Rod, yellow	123
(Nif)^−^·(2apH)^+^(4a)	Neat grinding, acetone assisted grinding, acetone	Rod, yellow	124
(Nif)^−^·(3apH)^+^(5)	Neat grinding, methanol assisted grinding, methanol	Rod, yellow	—
(Nif)^−^·(4apH)^+^(6)	Neat grinding, methanol assisted grinding, methanol	Rod, colorless	—
(Mef)_2_·(bpe) (7)	Neat grinding, methanol assisted grinding, methanol	Rod, colorless	188
(Mef)_2_·(bpee) (8)	Neat grinding, methanol assisted grinding, methanol	Rod, green	216
Mef·bpp (9)	Neat grinding, methanol assisted grinding, methanol	Block, colorless	192
(Mef)·(2apH)^+^·H_2_O (10)	Neat grinding, methanol assisted grinding, methanol	Block, red	118
Mef·*3ap* (11)	Neat grinding, methanol assisted grinding, methanol	Block, colorless	—
(Mef)^−^·(4apH)^+^·H_2_O (12)	Neat grinding, methanol assisted grinding, methanol	Block, colorless	125

## Materials and Methods

All the reagents (Nif, Mef, *bpe*, *bpee*, *bpp*, *2ap, 3ap,* and *4ap*) were purchased from Sigma-Aldrich and were used as received.

### Mechanochemical Reaction

The method was used to prepare new solid forms of Nif and Mef based molecules. API and coformer were ground in an agate mortar, either neat or in the presence of two drops of solvent (acetone/methanol/1,4-dioxane). PXRD was used to confirm the formation of the new phase. Good quality crystals suitable for SCXRD were grown by dissolving the powder in a suitable solvent (Table 1).

### Solvent Evaporation

Both API (1 mM) and conformer (1 mM) was dissolved in an appropriate solvent (2 ml) with gentle stirring till a transparent solution was obtained (usually 10–15 min). The clear solution was kept for crystallization at room temperature. In most cases, good-quality crystals were filtered after seven to 10 days. Ten new and four previously reported multicomponent solids of Nif and Mef were isolated in this study. Crystal data and structure refinement are summarized in Table 2.

**TABLE 2:** Crystal data and structure refinement of the solids **1**‒**12.**

	**1**	**2**	**2a**	**3**	**4**
Empirical formula	C38 H30 F6 N6 O4	C19 H14 F3 N3 O2	C21 H18 F3 N3 O3	C39 H32 F6 N6 O4	C18 H15 F3 N4 O2
Formula weight	748.68	373.33	417.38	762.71	376.34
Temperature (K)	298 (2)	298 (2)	298 (2)	298 (2)	298 (2)
Crystal system	Monoclinic	Monoclinic	Triclinic	Monoclinic	Monoclinic
Space group	*P*2_1_/n	*C*c	*P* ī	*C* 2/c	*C*c
*a* (Å)	10.6728 (16)	25.380 (6)	9.355 (5)	17.889 (4)	7.413 (2)
*b* (Å)	12.4719 (19)	12.495 (3)	10.722 (6)	7.4942 (18)	33.061 (10)
*c* (Å)	25.232 (4)	10.764 (3)	11.110 (6)	28.063(6)	7.690 (2)
*α* (°)	90.00	90.00	67.844 (11)	90.00	90.00
*β* (°)	92.169 (3)	92.040 (5)	79.027 (12)	104.669 (5)	109.745 (6)
*γ* (°)	90.00	90.00	75.084 (11)	90.00	90.00
V (Å^3^)	3356.3 (9)	3411.4 (13)	991.9 (9)	3639.6 (14)	1773.8 (9)
Z	4	4	2	4	4
D_calc_, (gcm^−3^)	1.482	1.454	1.398	1.392	1.409
µMoKα (cm^−1^)	0.121	0.119	0.114	0.113	0.116
Goodness-of-fit (GOF) on *F* ^2^	1.073	1.025	0.998	1.160	1.060
*λ* (Å)	0.71073	0.71073	0.71073	0.71073	0.71073
R_1,_ *w*R_2_[I > 2σ (*I*)]^a^	0.0684,0.1524	0.0896,0.1461	0.0902,0.2861	0.0881,0.2493	0.0429,0.1119
CCDC	1574267	1574270	1574266	1574275	1574269

—	**4a**	**5**	**6**	**7**	**8**
Empirical formula	C18 H15 F3 N4 O2	C18 H15 F3 N4 O2	C18 H15 F3 N4 O2	C21 H21 N2 O2	C21 H20 N2 O2
Formula weight	376.34	376.34	376.34	333.40	332.39
Temperature (K)	298 (2)	298 (2)	298 (2)	298 (2)	298 (2)
crystal system	Triclinic	Orthorhombic	Monoclinic	Triclinic	Triclinic
Space group	*P* ī	*Pbca*	*C*c	*P* ī	*P* ī
*a* (Å)	8.0525 (5)	7.8043 (3)	14.6959 (16)	7.783 (2)	7.814 (2)
*b* (Å)	8.1125 (5)	12.2520 (5)	10.4708 (16)	8.866 (3)	8.787 (2)
*c* (Å)	25.6798 (18)	34.9035 (15)	22.935 (3)	13.261 (4)	13.111 (3)
*α* (°)	87.750 (2)	90.00	90.00	101.121 (5)	99.996 (5)
*β* (°)	83.626 (2)	90.00	95.197 (3)	98.179 (6)	98.722 (5)
*γ* (°)	89.763 (2)	90.00	90.00	92.780 (5)	92.125 (6)
V (Å^3^)	1665.90 (19)	3337.4 (2)	3514.8 (8)	886.0 (5)	874.4 (4)
Z	4	8	8	2	2
D_calc_, (gcm^−3^)	1.500	1.498	1.422	1.250	1.263
µMoKα (cm^−1^)	0.123	0.123	0.117	0.081	0.082
Goodness-of-fit (GOF) on *F* ^2^	1.057	0.958	1.075	1.145	1.078
*λ* (Å)	0.71073	0.71073	0.71073	0.71073	0.71073
R_1,_ *w*R_2_[I > 2σ(*I*)]^a^	0.0459, 0.1227	0.0408, 0.1328	0.0705, 0.1828	0.0799, 0.1901	0.0740, 0.1943
CCDC	1574277	2089680	1574268	1574274	1574271

—	**9**	**10**	**11**	**12**
Empirical formula	C28 H29 N3 O2	C20 H23 N3 O3	C20 H21 N3 O2	C20 H23 N3 O3
Formula weight	439.54	353.41	335.40	353.41
Temperature (K)	298 (2)	298 (2)	298 (2)	298 (2)
crystal system	Triclinic	Triclinic	Triclinic	Monoclinic
Space group	*P* ī	*P* ī	*P* ī	*P*2_1_/n
*a* (Å)	7.675 (3)	7.954 (3)	6.0177 (5)	7.785 (2)
*b* (Å)	7.828 (3)	8.027 (3)	11.1933 (9)	8.359 (2)
*c* (Å)	20.462 (7)	15.822(5)	13.7316 (13)	28.691 (8)
*α* (°)	88.438 (7)	83.883 (7)	79.420 (4)	90.00
*β* (°)	86.370 (6)	85.659 (7)	78.913 (4)	95.470 (6)
*γ* (°)	79.737 (7)	66.931 (7)	75.134 (3)	90.00
V (Å^3^)	1207.2 (7)	923.5 (6)	868.45 (13)	1858.5 (9)
Z	2	2	2	4
D_calc_, (g cm^−3^)	1.209	1.271	1.283	1.263
µMoKα (cm^−1^)	0.077	0.087	0.084	0.086
Goodness-of-fit (GOF) on *F* ^2^	0.917	1.111	1.083	1.031
*λ* (Å)	0.71073	0.71073	0.71073	0.71073
R_1,_ *w*R_2_[I > 2σ(*I*)]^a^	0.0503, 0.1790	0.0936, 0.2462	0.0517, 0.1471	0.0700, 0.2244
CCDC	1574272	1574273	2089681	2089918

### X-Ray Structure Determination

X-ray diffraction studies of crystals mounted on a capillary were carried out on a BRUKER AXS SMART-APEX diffractometer with a CCD area detector (MoKα = 0.71073 Å, monochromator: graphite) (Bruker Analytical X-ray Systems, 2000, SMART: Bruker Molecular Analysis Research Tool, Madison, WI, 2000). Frames were collected at T = 298 K by ω, ϕ and 2θ-rotation at 10 s per frame with SAINT (Bruker Analytical X-ray Systems, SAINT-NT., 2001). The measured intensities were reduced to F^2^ and corrected for absorption with SADABS (Bruker Analytical X-ray Systems, 2001, SAINT-NT., 2001). Structure solution, refinement, and data output were carried out with the SHELXTL program suite on the Olex-2 platform (Dolomanov et al., 2009). Non-hydrogen atoms were refined anisotropically. C−H hydrogen atoms were placed in geometrically calculated positions by using a riding model. O−H and N−H hydrogen atoms were localized by difference Fourier maps and refined in subsequent refinement cycles. Images were created with Crystal Impact Diamond software ((Putz et al., nd) Visual Crystal Structure Information, http://www.ccp14.ac.uk/ccp/web-mirrors/crystalimpact/diamond/publ/jac). Hydrogen bonding interactions in the crystal lattice were calculated with SHELXTL.

### Solubility Studies

Solubility of all multicomponent solids reported here was determined using UV-Vis method reported by Choudhury et al. (Karanam and Choudhury, 2013; Joshi and Roy Choudhury, 2018), and Higuchi and Connor in 1965 (Higuchi and Connors, 1965). A measured quantity of each solid was completely dissolved in a large excess of distilled water (pH = 6.8). The stock solutions were suitably diluted to get absorbance values within 1 in the UV-vis spectrum and to prepare standard solutions for generating the calibration curves. The λ_max_ values of Nif/Mef in all the salts were then determined using a PerkinElmer Lambda 1050 UV/Vis/NIR spectrophotometer with a quartz cuvette. The absorbance values of the primary standard solutions were determined at the respective λ_max_ values. The absorbance values were plotted in the y-axis, and the concentrations were plotted in the x-axis, and the points were fitted to a straight line (calibration curve). Simultaneously, a suspension of Nif/Mef in distilled water was stirred at room temperature for 24 h. The excess solids were filtered, and the solution was diluted to get the absorbance value within 1 in the UV-vis spectrum. PXRD analysis of the residual solids was carried out to assess the nature of the solid forms. The absorbance of the clear diluted solution was determined at λ_max_ of Nif/Mef for all solids, and the concentration of the salt was determined using the calibration curve, which was generated earlier. The solubility of all the solids was measured at 33°C. The solubility was calculated by multiplying the concentration of the dilute solution by 1,000.

Solubility of all the salts and cocrystals reported in this study was measured and compared with free acids in similar conditions. Crystalline salts of Nif and Mef showed considerable improvement in solubility with aminopyridine coformers compared with bipyridine coformers. In the case of Nif based solids, the salt of Nif with *2ap* coformer showed higher solubility while the cocrystals of Nif with *bpe* and *bpee* exhibited a decrease in solubility. The salt of *2ap* with Mef showed the most remarkable solubility improvement among Mef based solids, while *bpe* and *bpee* based solids showed only marginal improvement. We were unsuccessful to correlate the solubility with the percentage of occurrence of various noncovalent interactions. The solubility trends for Nif and Mef based multicomponent solids have been depicted in Supplementary Figure S3. Solubility of the solids **4a, 5,** and **11** were not measured due to lack of purity in the samples.

### Other Physical Measurements

DSC analysis was carried out using a PerkinElmer DSC system on well-ground samples under a nitrogen atmosphere at the rate of 10°C min^−1^. Room-temperature powder X-ray diffraction data were collected on a Bruker D8 Advance diffractometer using Ni-filtered CuKα radiation. Data were collected with a step size of 0.05 and at a count time of 1 s per step over the range 10° < 2θ < 50°. A Rietveld treatment of the powder diffraction data of the powder sample was carried out based on single-crystal data using TOPAS 4.2, Bruker to ascertain the homogeneity of the bulk sample (Supplementary Figure S1) (Coelho, 2018, https://www.bruker.com/products/x-ray-diffraction-and-elemental-analysis/x-ray-diffraction/xrd-software/topas.html).

## Results and Discussion

**SCHEME 4:**
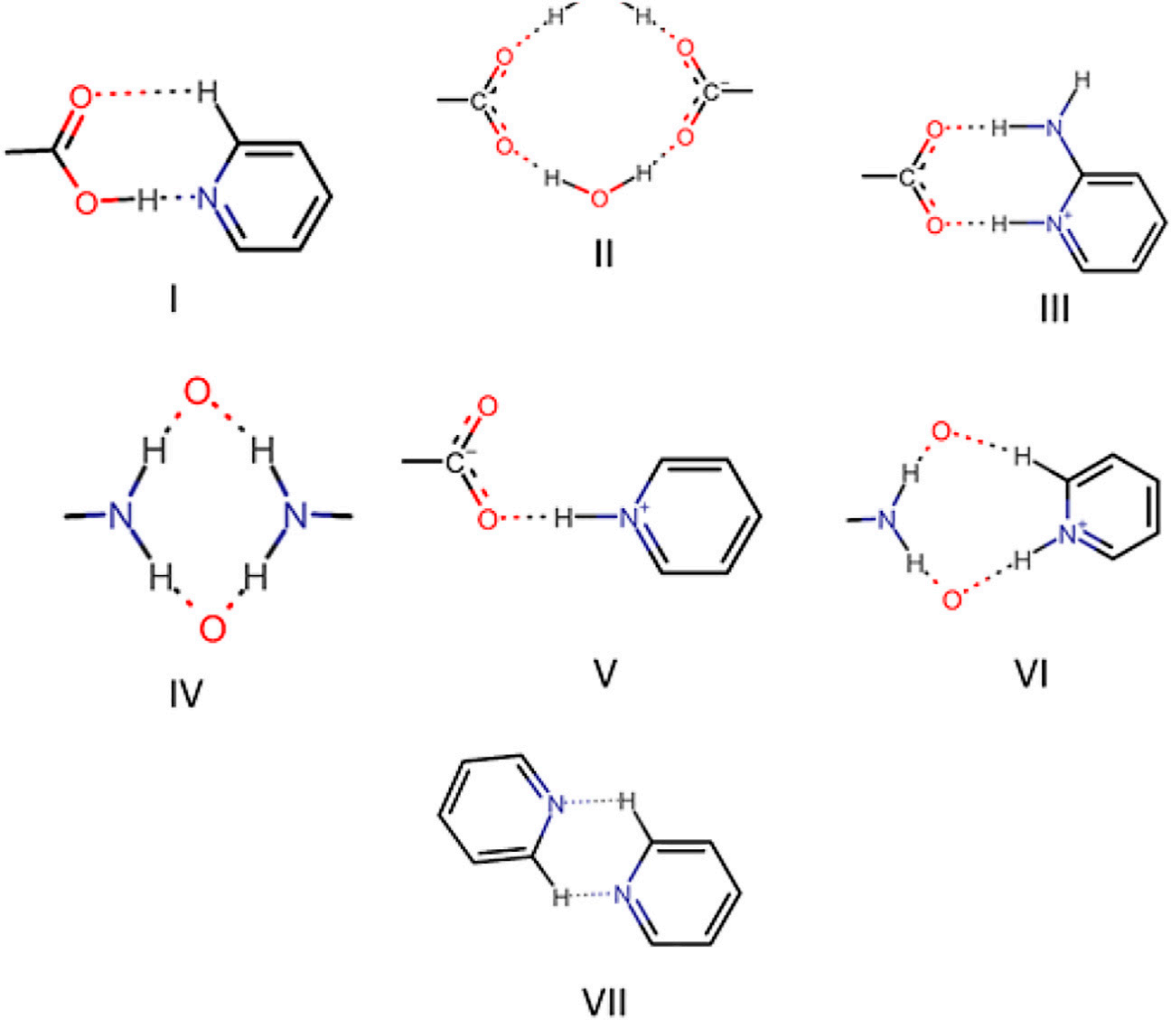
Different types of synthons as observed in the solids **1-12.**

The robust acid-pyridine synthon is the main driving force for the formation of multicomponent solids. In some solids, a proton was transferred from acid to pyridine; in selected cases, solvent/water molecule was included in the crystal. Although π∙∙∙π, C─H∙∙∙π, and C─F∙∙∙H─C interaction played a significant role in the structure formation of all the solids, acid-pyridine synthon remained decisive in dictating the crystal structures. All major synthons involved in this study are shown in Scheme 4. In this system, variation of composition and solvent affected the outcome of crystallization only in two cases. In general, bipyridine coformers, namely, *bpe, bpee,* and *bpp,* yielded solids with 2:1 composition as the trimeric acid-pyridine synthon is the main driving force for the supramolecular aggregation. However, solvent variation led to two exceptions: solvated Nif*bpee* and salt polymorphs Nif·*2ap*. In the case of aminopyridines as coformers, the outcome of crystallization was a 1:1 salt with Nif based solids. Interestingly, Mef based solids resulted in the form of salt monohydrates.

### Crystal Structure of Bipyridine Based Solids

**FIGURE 1:**
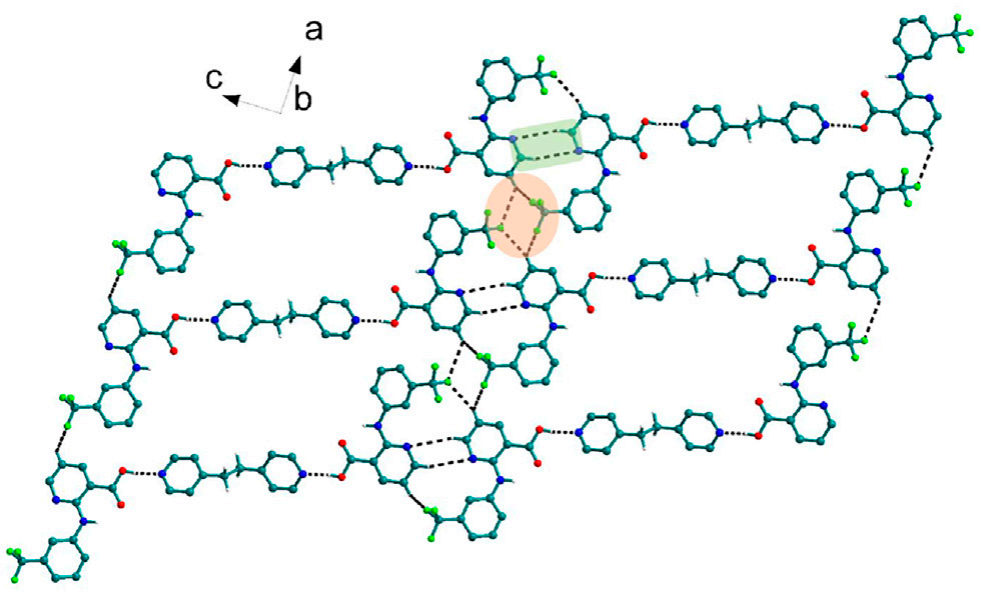
Nif─*bpe*─Nif trimers are interacted *via* C─H∙∙∙N (synthon VII) and C─H∙∙∙F interaction, forming a planar sheet.

**FIGURE 2:**
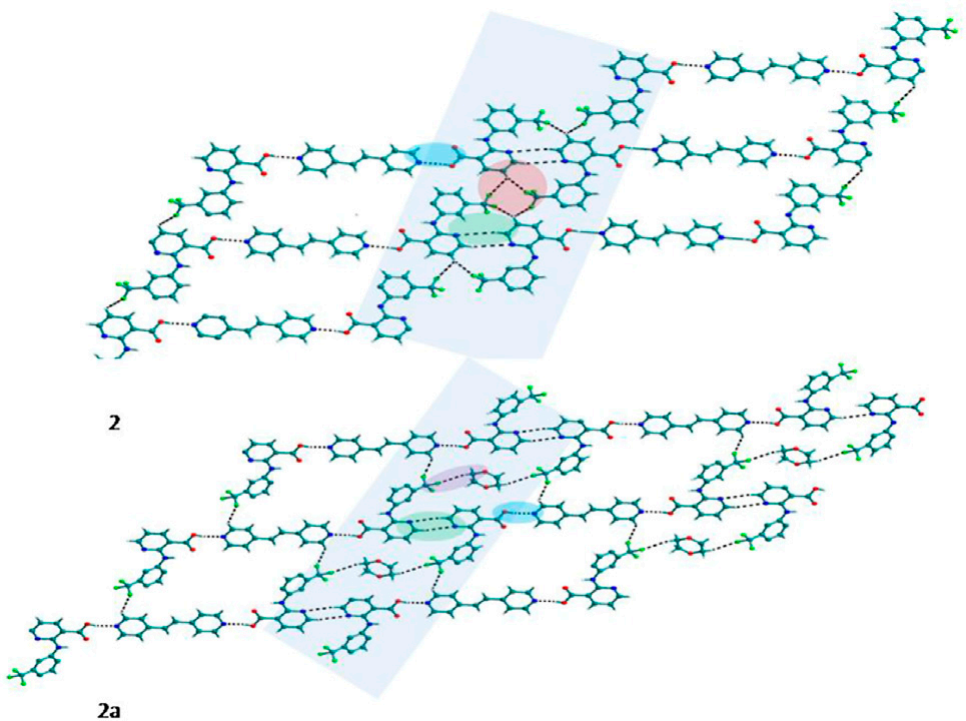
In **2** and **2a**, Nif─*bpee*─Nif trimers are interconnected via C─H∙∙∙N and C─H∙∙∙F interactions, forming a planar sheet.

**FIGURE 3:**
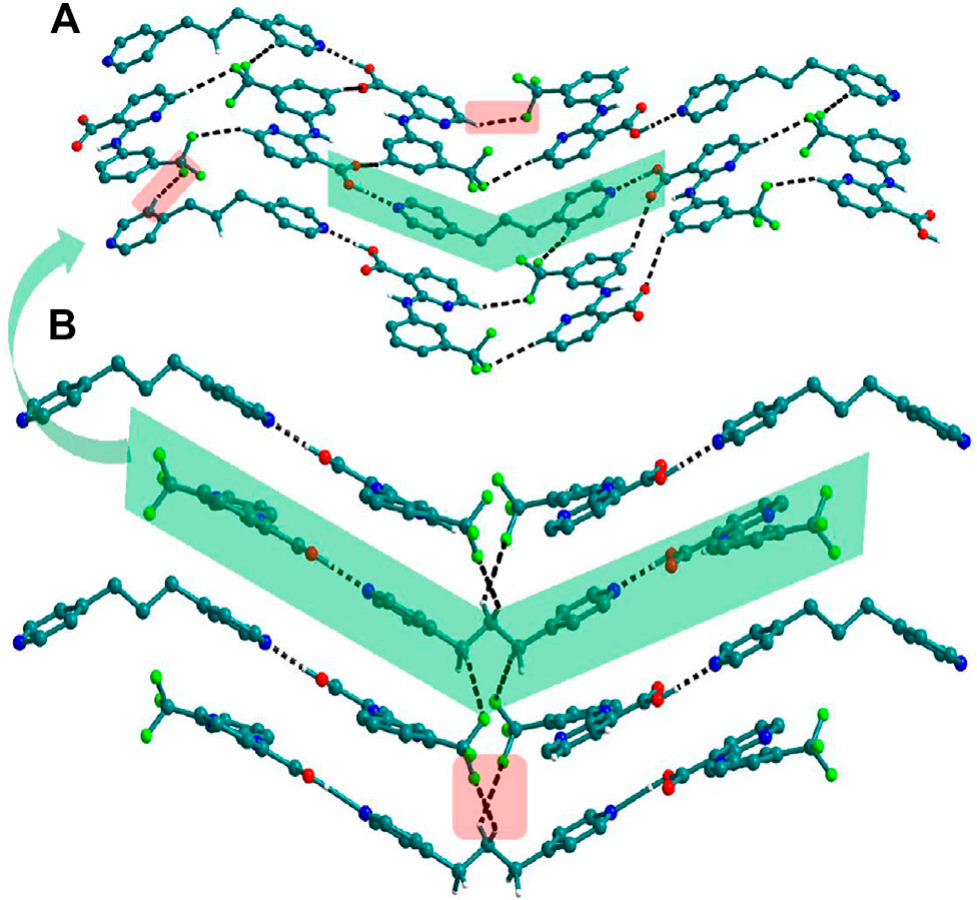
**(A)** In **3**, Nif─*bpp*─Nif trimers are connected with other trimers forming a planar sheet *via* C─H∙∙∙F interaction. **(B)** Different sheets further connect via other C─H∙∙∙F interactions to form a 3D structure.

**FIGURE 4:**
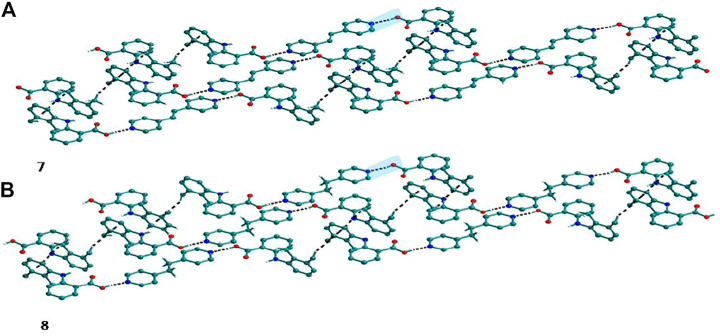
**(A)** Mef─*bpe*─Mef trimers are connected with other trimers via C─H∙∙∙π interaction in **7**. **(B)** Isostructural **8** shows the same types of Mef─*bpee*─Mef trimers interactions.

**FIGURE 5:**
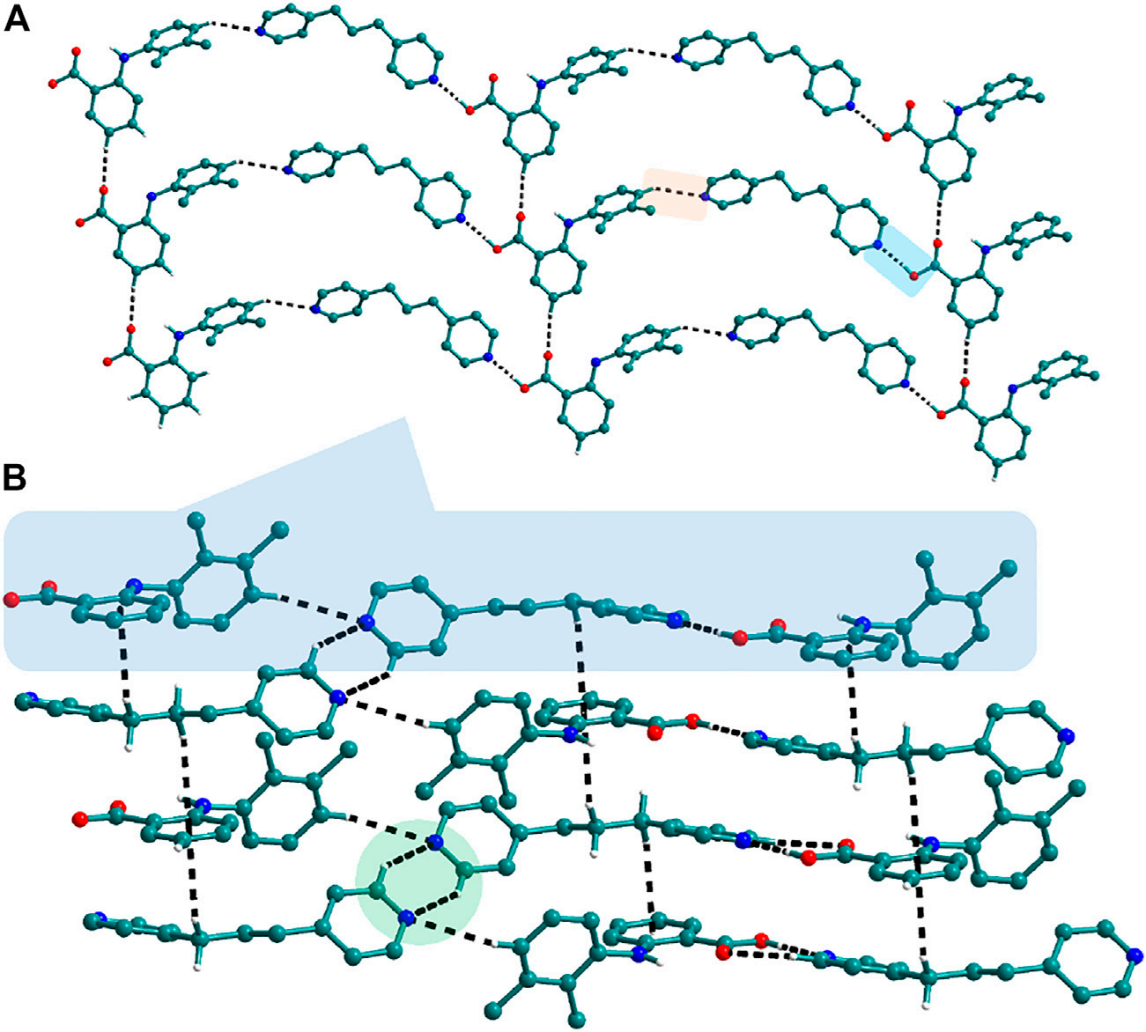
**(A)** Nif─bpp dimers interconnected *via* C─H∙∙∙N forming a planar sheet in **9**. **(B)** Planar sheets *via* C─H∙∙∙π and C─H∙∙∙N interaction are stacked one over the other, forming a 3D structure.

Bipyridine-based coformers like *bpe*, *bpee* and *bpp* formed solids **1**, **2**, **2a** and **3** with Nif and **7**, **8,** and **9** with Mef. The reaction of Nif and *bpe* formed a 2:1 cocrystal **1,** where two molecules of Nif and one molecule of *bpe* are present in the asymmetric unit. In **1**, the trimer Nif–*bpe*–Nif, driven by acid-pyridine planar heterosynthon I (Scheme 4), is the main building block. The trimers interact through C─H∙∙∙N (3.08 Å) and C─H∙∙∙F (2.591, 3.022, 2.807, and 3.486 Å), forming a 2D planar sheet (Figure 1). Nif cocrystallized with *bpee,* forming a 2:1 cocrystal **2,** which is isostructural with solid **1**. In **2**, Nif─*bpee*─Nif trimers connect with each other through C─H∙∙∙N (3.143 Å) and C─H∙∙∙F (2.653, 3.652, 2.653, and 3.652 Å) interactions (Figure 2). The reaction of Nif with *bpee* in 1,4-dioxane as solvent formed solid **2a.** The only difference between the structure of **2a** and the previous two solids is the inclusion of 1,4-dioxane solvent in the crystal structure. In **2a**, two trimers are bridged through 1,4-dioxane via C─H-F (2.669 Å) instead of a ring formation (Figure 2). The reaction of Nif with *bpp* formed a 2:1 cocrystal **3** with one molecule of Nif and half a molecule of *bpp* in the asymmetric unit. The trimer formed with acid-pyridine synthon again connected through C─H∙∙∙F (2.580 Å and 2.760 Å), resulting in a planar sheet; the sheets are further linked via other C─H∙∙∙F (3.442 Å) interactions (Figure 3). C─H∙∙∙N interactions formed by the nitrogen of the pyridyl group in Nif were also observed in the solids **1**, **2** and **2a** but were absent in **3**. The reaction of Mef with *bpe* and *bpee* formed two isostructural solids **7** and **8**. The acid∙∙∙pyridine heterosynthon I (Scheme 4) was the major synthon as expected. In both the solids, the trimers are connected through the C─H∙∙∙π bond (3.435 and 3.367 Å) with other trimers on the *bc*-plane, forming a three-dimensional network (Figure 4). Interestingly, cocrystallization of Mef with *bpp* yielded **9**, wherein a dimer was the building block instead of the usual trimer. The asymmetric unit showed the presence of one molecule of Mef and *bpp* each. The other pyridine nitrogen of the dimer interacted with the other dimer via C─H∙∙∙N (2.899 Å) interaction, forming a planar sheet (Figure 5A). The sheets are stacked one over the other via C─H∙∙∙π (2.879, 2.933 and 2.897 Å) and C─H∙∙∙N (2.837 Å) interactions (Figure 5B).

### Crystal Structures of Aminopyridine Based Solids

**FIGURE 6:**
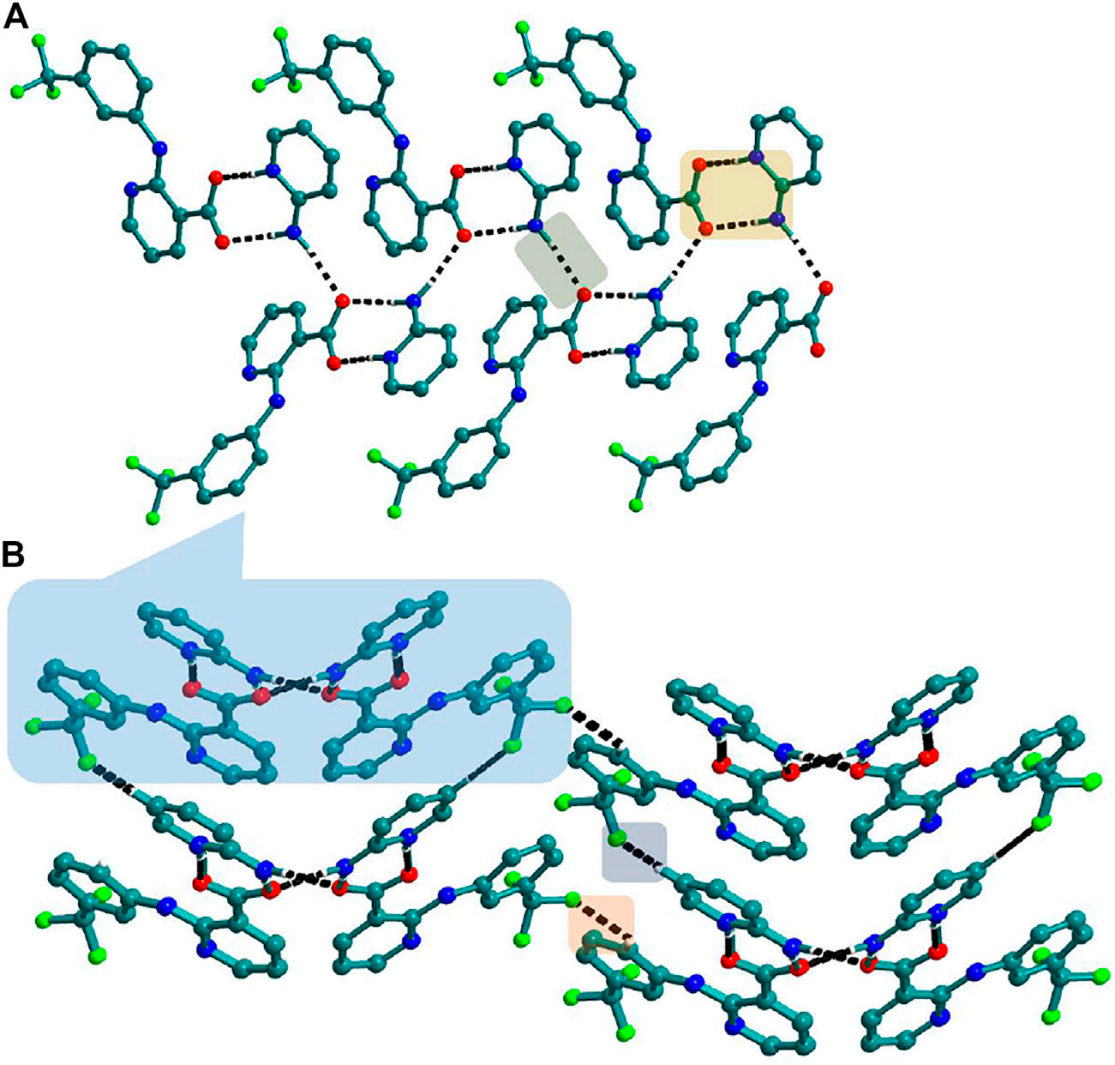
**(A)** Nif─*2ap* dimers interacted with other dimers via N─H∙∙∙O to form a 1D Chain in **4**. **(B)** The chains are connected through C─H∙∙∙F in the other two planes forming a 3D network.

**FIGURE 7:**
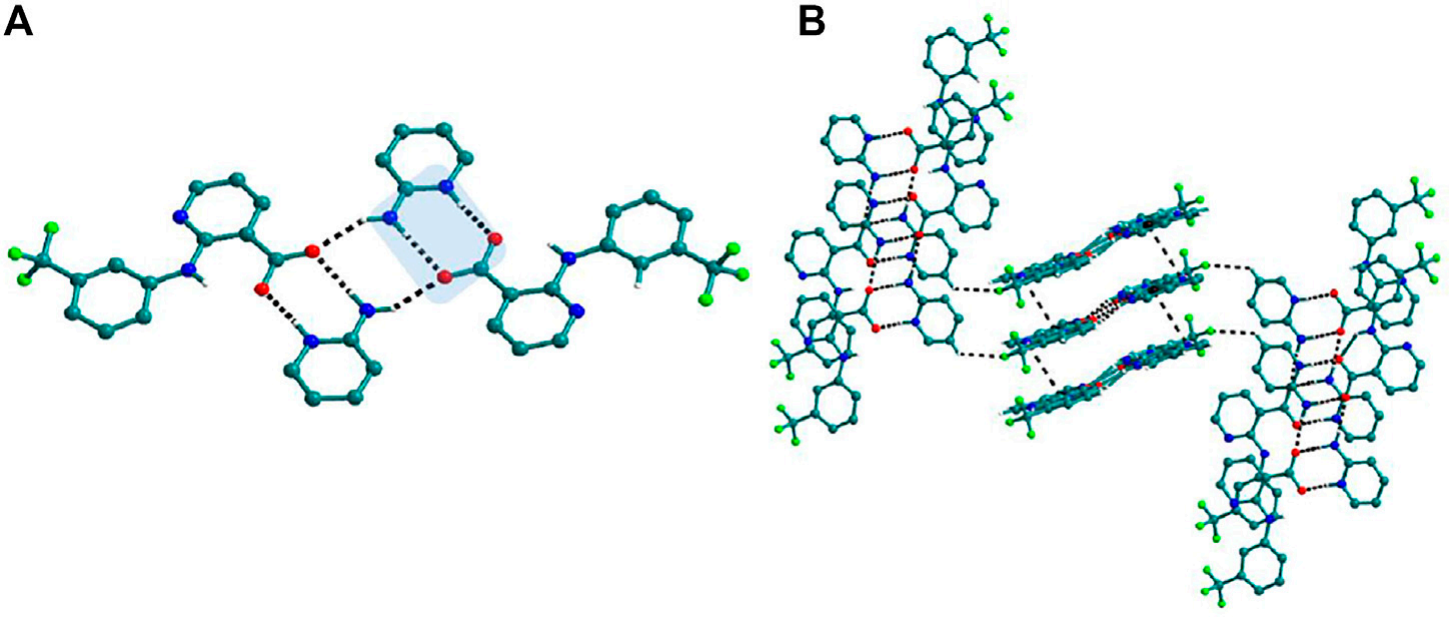
**(A)** Interaction of Nif─*2ap* dimers via N─H∙∙∙O with other dimers to form a tetramer, in **4a**. **(B)** Stacking of tetramer through C−H∙∙∙π interaction one over the other and cross-sects each other via C−H∙∙∙F interaction to form a 3D structure.

**FIGURE 8:**
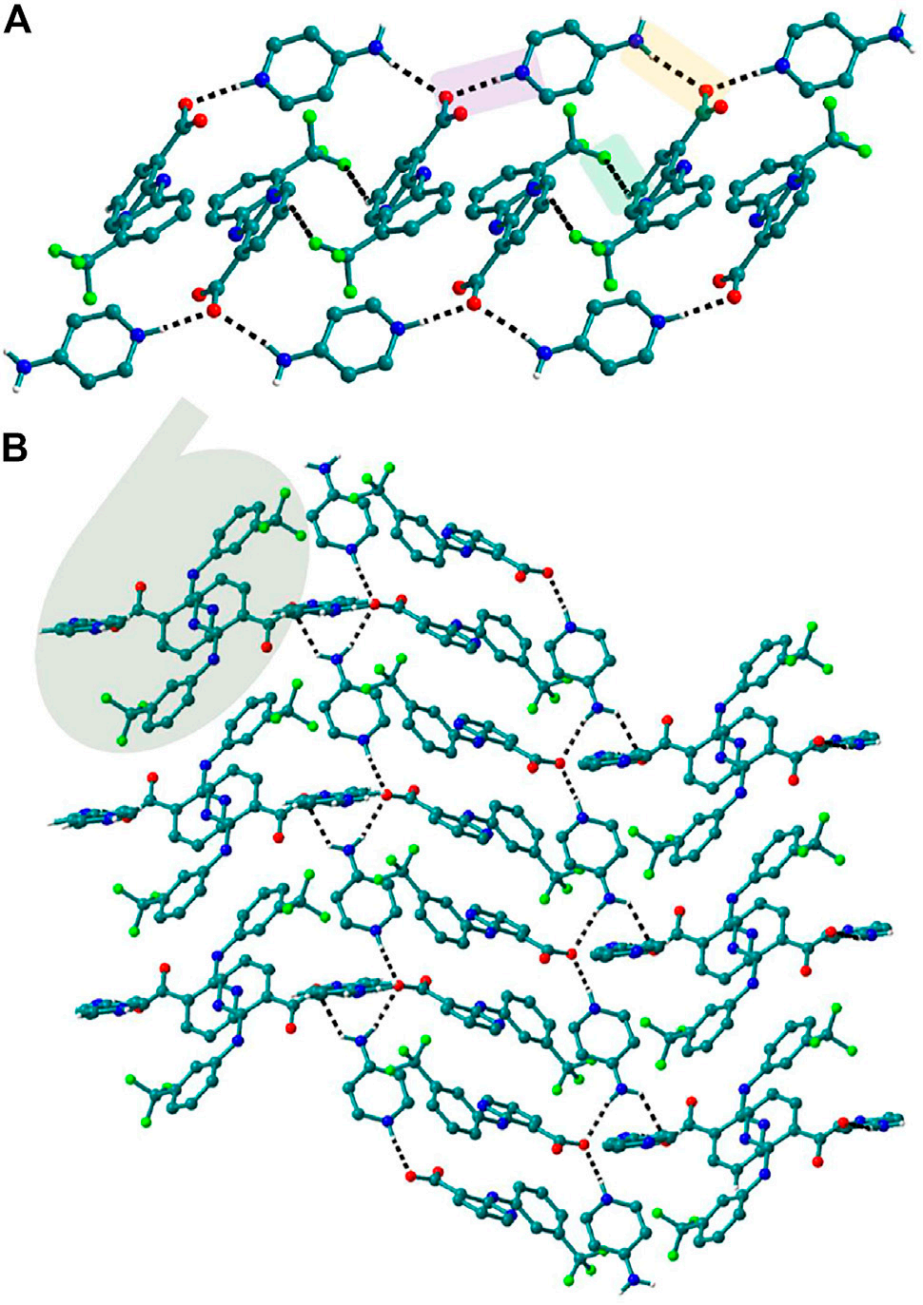
**(A)** In **6**, Nif─*4ap* dimers interacted with other dimers via N─H∙∙∙O, forming a 1D Chain. **(B)** The chains are interconnected *via* C─H∙∙∙F and C─H∙∙∙O interaction in a perpendicular fashion.

**FIGURE 9:**
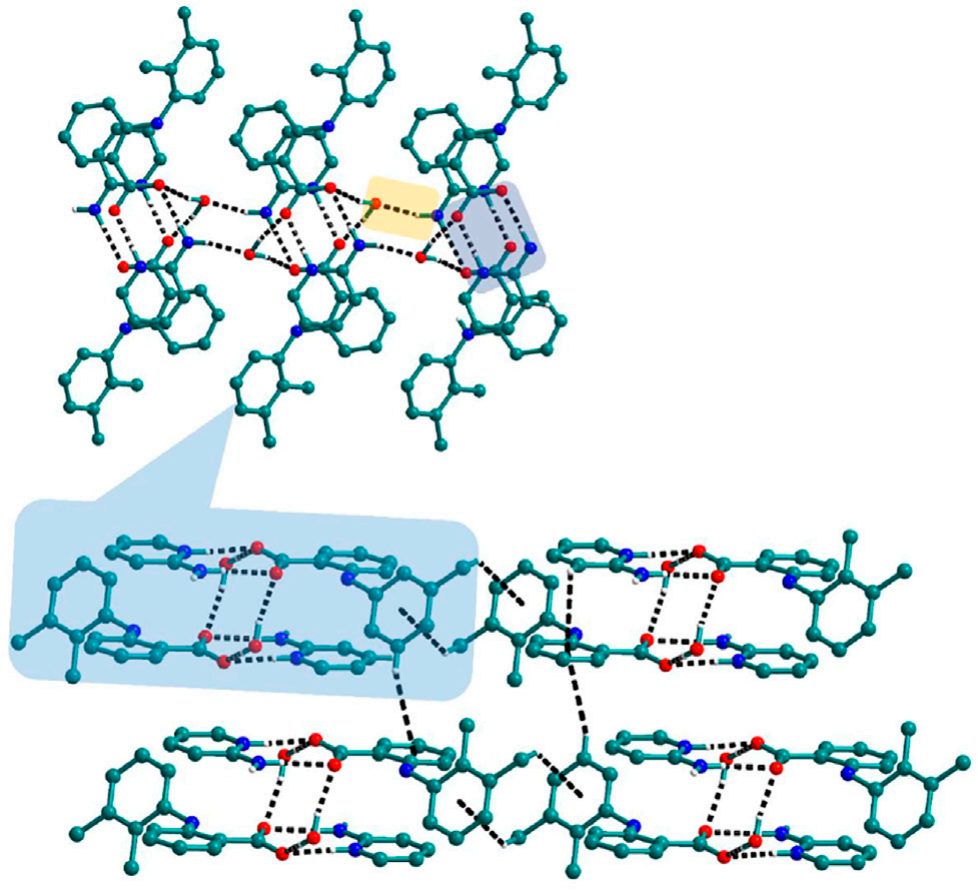
In **10**, The dimers of Mef─*2ap* are connected with other dimers through N─H∙∙∙O forming columns. These columns further connect with other columns via C─H∙∙∙π to form a 3D network.

**FIGURE 10:**
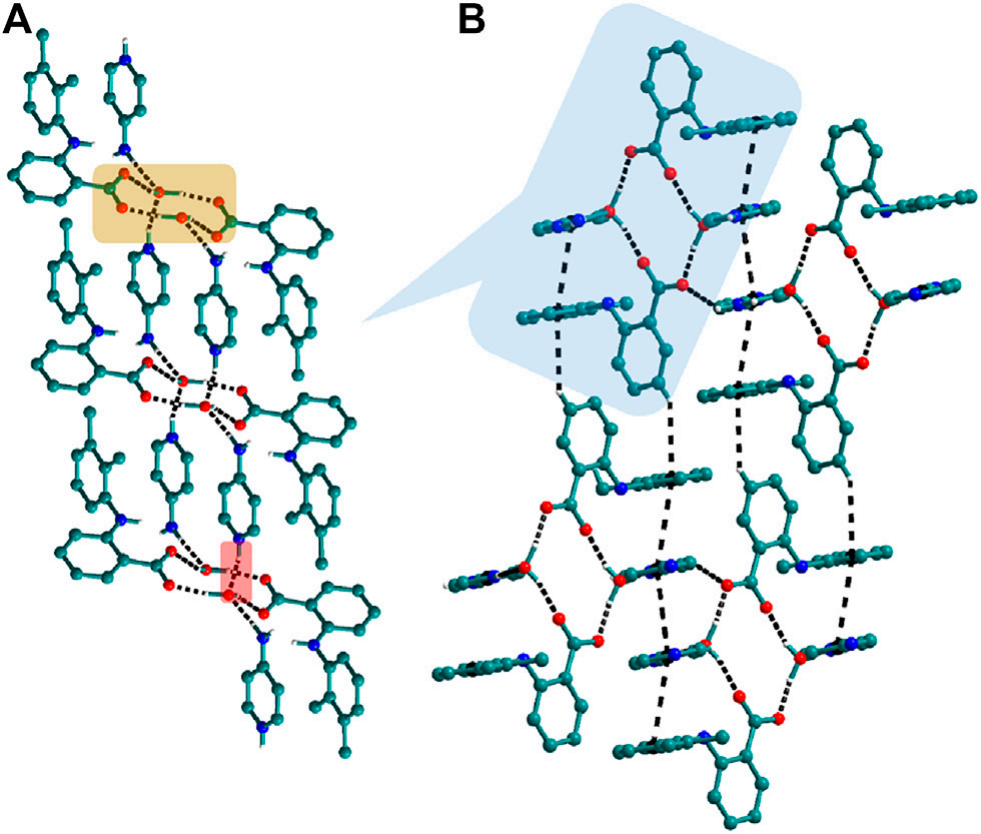
**(A)** In **12**, tetramers of Mef─H_2_O─Mef are formed *via* synthon II which further connected through N─H∙∙∙O with other tetramers forming columns. **(B)** These columns, interacting with others *via* C─H∙∙∙π and π∙∙∙π interactions, form a 3D network.

The use of aminopyridine coformers *2ap, 3ap,* and *4ap* with Nif led to the isolation of the solids **4**, **4a**, **5,** and **6** while Mef yielded the solids **10, 11,** and **12.** The reaction of Nif and *2ap* formed a 1:1 salt **4** and its solvate, **4a**. In **4**, one molecule each of Nif and *2ap* is present in the asymmetric unit. In the dimer formed between Nif and *2ap*, the proton transfer from the oxygen of the carboxylate group in Nif to nitrogen of pyridyl group in *2ap* ensured the heterosynthon III (Scheme 4) as the main building block. These dimers are connected to each other via N─H∙∙∙O (2.058 Å), where the amino group of *2ap* and the other oxygen of carboxylate acting as donor and acceptor, respectively, forming 1D H-bonded chains (Figure 6A); the chains are further interconnected via C─H∙∙∙F (2.669 and 2.586 Å) to form a 3D network (Figure 6B). The salt solvate **4a** was isolated when crystallization was carried out in a different solvent (Table 1). In **4a**, two molecules, each of Nif and *2ap,* are present in the asymmetric unit. The dimer formed through the heterosynthon III (N─H∙∙∙O: 2.647 and 2.857 Å) (Scheme 4) is the main building block. The dimers are further connected via heterosynthon IV (N─H∙∙∙O: 2.871 Å) (Scheme 4), forming a tetramer (Figure 7A). These tetramers are stacked one over the other through C−H∙∙∙π interactions (3.242 and 3.581 Å) forming a layer; these layers cross-link each other *via* C−H∙∙∙F (3.682 Å) interactions (Figure 7B). Solid **5** contains one molecule each of Nif and *3ap* in the asymmetric unit. The solid **6** formed by the reaction of Nif and *4ap*, where again dimer formation took place via heterosynthon V (Scheme 4), which are further connected with other dimers through N─H∙∙∙O (2.228 Å), C─H∙∙∙F (2.605 Å), and N─H∙∙∙O (2.228 Å) through the oxygen of the carboxylate moiety of Nif and the amine moiety of *4ap*, forming a chain. These chains are further connected with other chains in a perpendicular fashion via C─H∙∙∙O (2.495 Å) and N─H∙∙∙O (2.311 Å) interactions, forming a 3D network (Figure 8). Interestingly, the reaction of Mef with aminopyridine coformers *2ap, 3ap,* and *4ap* formed two salt hydrates **(**solids **10** and **12)** with the composition 1:1:1 and a cocrystal (solid **11**) with the composition 1:1. In **10,** Mef and *2ap* formed a dimer via synthon III (Scheme 4) like in **4**. However, interaction of the dimers was facilitated via water molecules through N─H∙∙∙O (2.007 Å) forming a chain. It should be noted that a similar observation was not found in Nif·*2ap*, solid **4**. These two chains are further mediated by water molecules forming a column involving synthon II *via* O─H∙∙∙O (1.842 Å and 1.947 Å). These columns further extend through C─H∙∙∙π (3.173 Å and 3.677 Å) to the other two dimensions (Figure 9). The reaction between Mef and *4ap* produced a salt hydrate **12**, which was recently reported by Trivedi et al. (Nechipadappu and Trivedi, 2017). Surprisingly, there is no acid-pyridine synthon observed in this structure; instead, the nitrogen of the pyridyl group of *4ap* interacted with the oxygen of the water molecule. The water mediates a pair of carboxylate dimers from two Mef, forming a tetramer via synthon II (Scheme 4). The tetramers further interact with *4ap* through N─H∙∙∙O (1.967 Å and 2.001 Å), forming a column. These columns are further linked to each other through synthon VII, C─H∙∙∙O (2.523 Å), C─H∙∙∙π (3.132 Å), and π∙∙∙π (3.640 Å) interactions (Figure 10).

### Hirshfeld Surface Analysis

**FIGURE 11:**
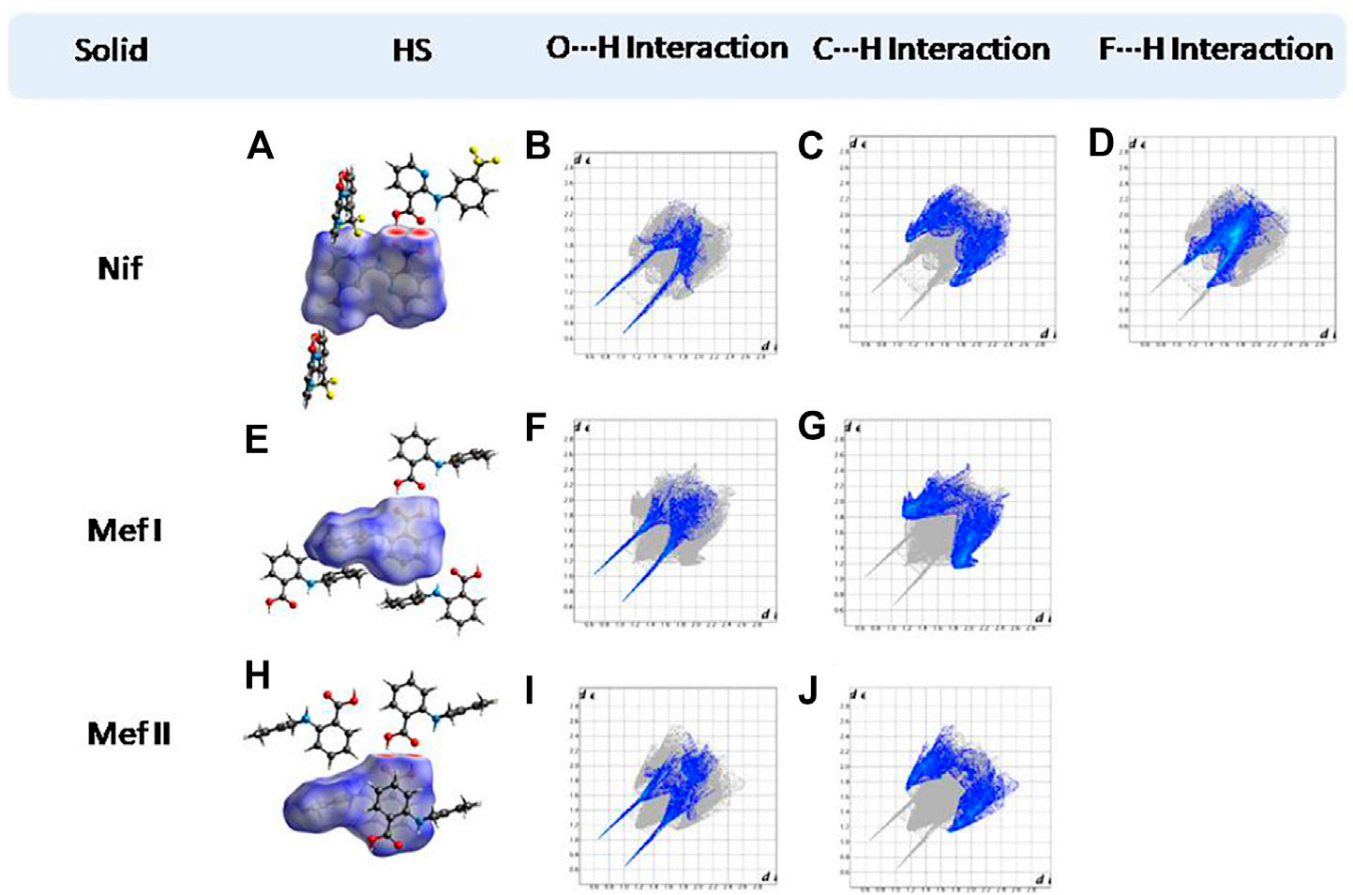
**(A,E,H)** Hirshfeld surface analysis and structural environment of Nif, Mef I, and Mef II. **(B–D)** O∙∙∙H/H∙∙∙O, C∙∙∙H/H∙∙∙C, and F∙∙∙H/H∙∙∙F interactions resolved fingerprint plots of Nif. **(F,G)** and **(I,J)** O∙∙∙H/H∙∙∙O and C∙∙∙H/H∙∙∙C interactions resolved fingerprint plots of both forms of Mef. The two spikes present in the solids are characteristic of the carboxylic acid dimer.

**FIGURE 12:**
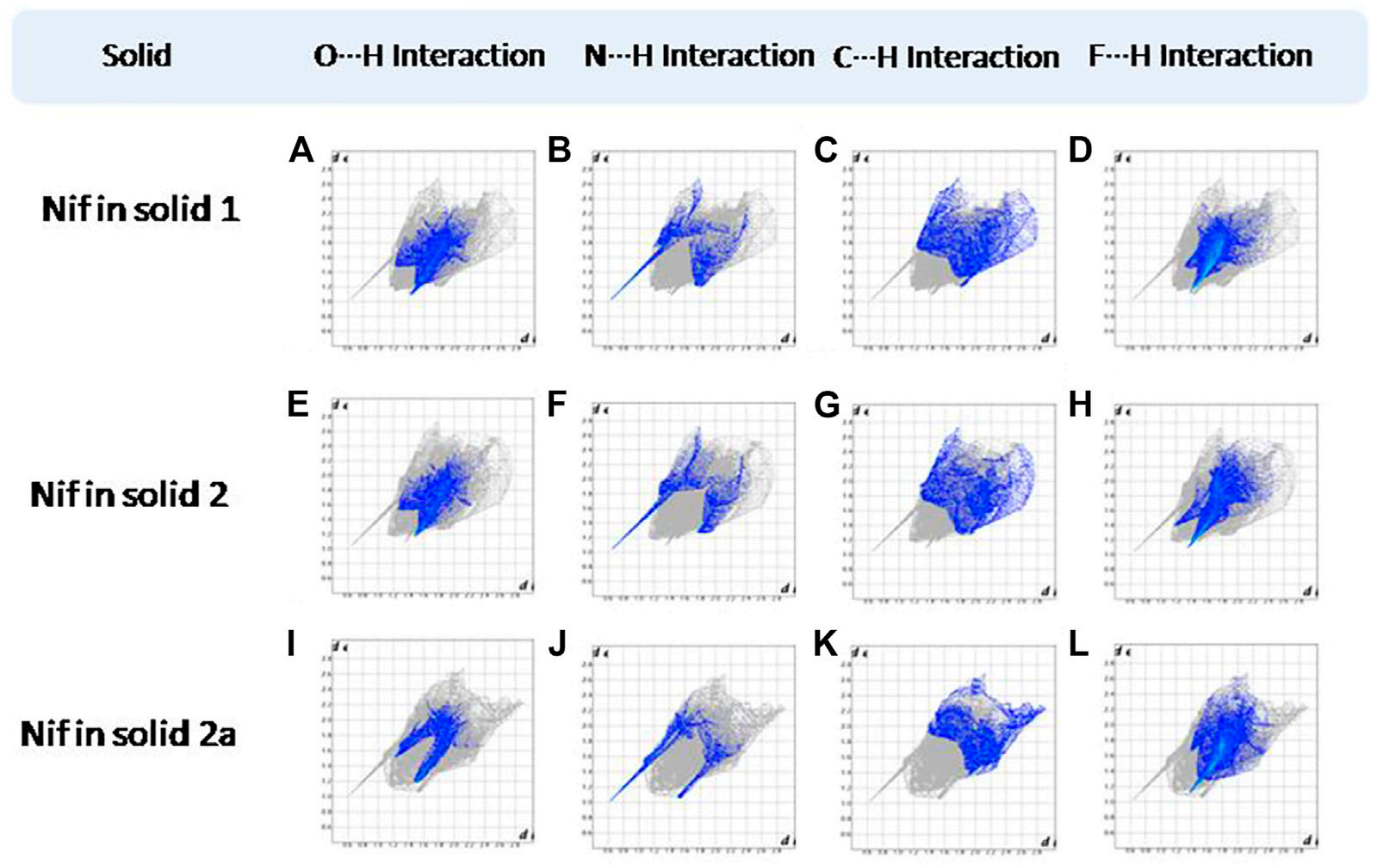
**(A–D)** O∙∙∙H/H∙∙∙O N∙∙∙H/H∙∙∙N, C∙∙∙H/H∙∙∙C, and F∙∙∙H/H∙∙∙F interactions Resolved fingerprint plots of Nif in solid **1**, respectively. **(E–L)** O∙∙∙H/ H∙∙∙O and N∙∙∙H/H∙∙∙N interactions resolved fingerprint plots of Nif of solid **2** and **2a**, respectively.

**FIGURE 13:**
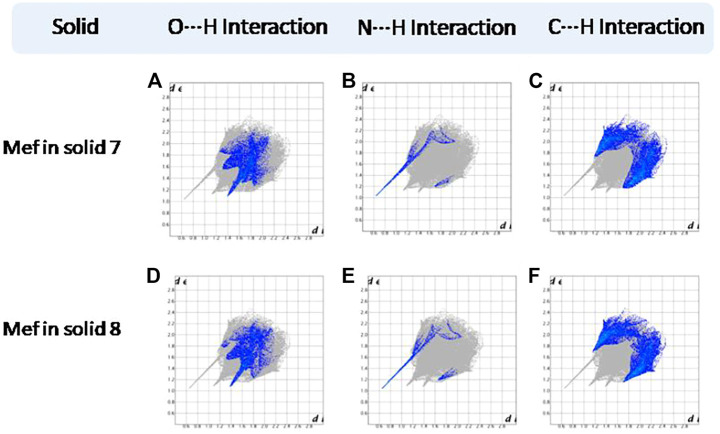
**(A–F)** Resolved fingerprint plots of Mef of **7** and **8** in O∙∙∙H/ H∙∙∙O, N∙∙∙H/H∙∙∙N, and C∙∙∙H/ H∙∙∙C interactions, respectively.

**FIGURE 14:**
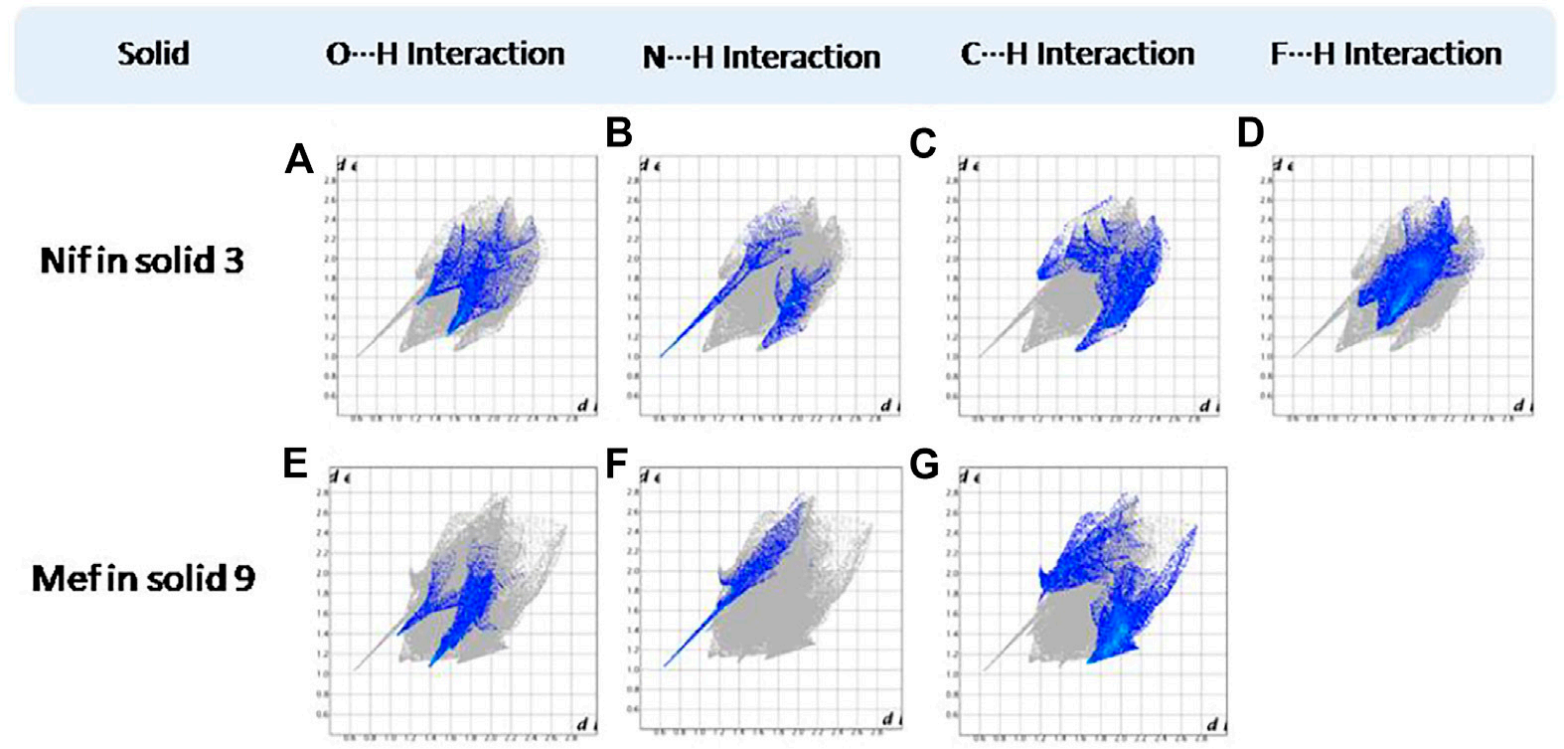
**(A–D)** Resolved fingerprint plots of Nif of solid **3** in O∙∙∙H/ H∙∙∙O, N∙∙∙H/H∙∙∙N, C∙∙∙H/ H∙∙∙C, and F∙∙∙H/ H∙∙∙F interactions, respectively. **(E–G)** Resolved fingerprint plots of Mef of solid **9** in O∙∙∙H/ H∙∙∙O, N∙∙∙H/H∙∙∙N, and C∙∙∙H/H∙∙∙C interactions, respectively.

**FIGURE 15:**
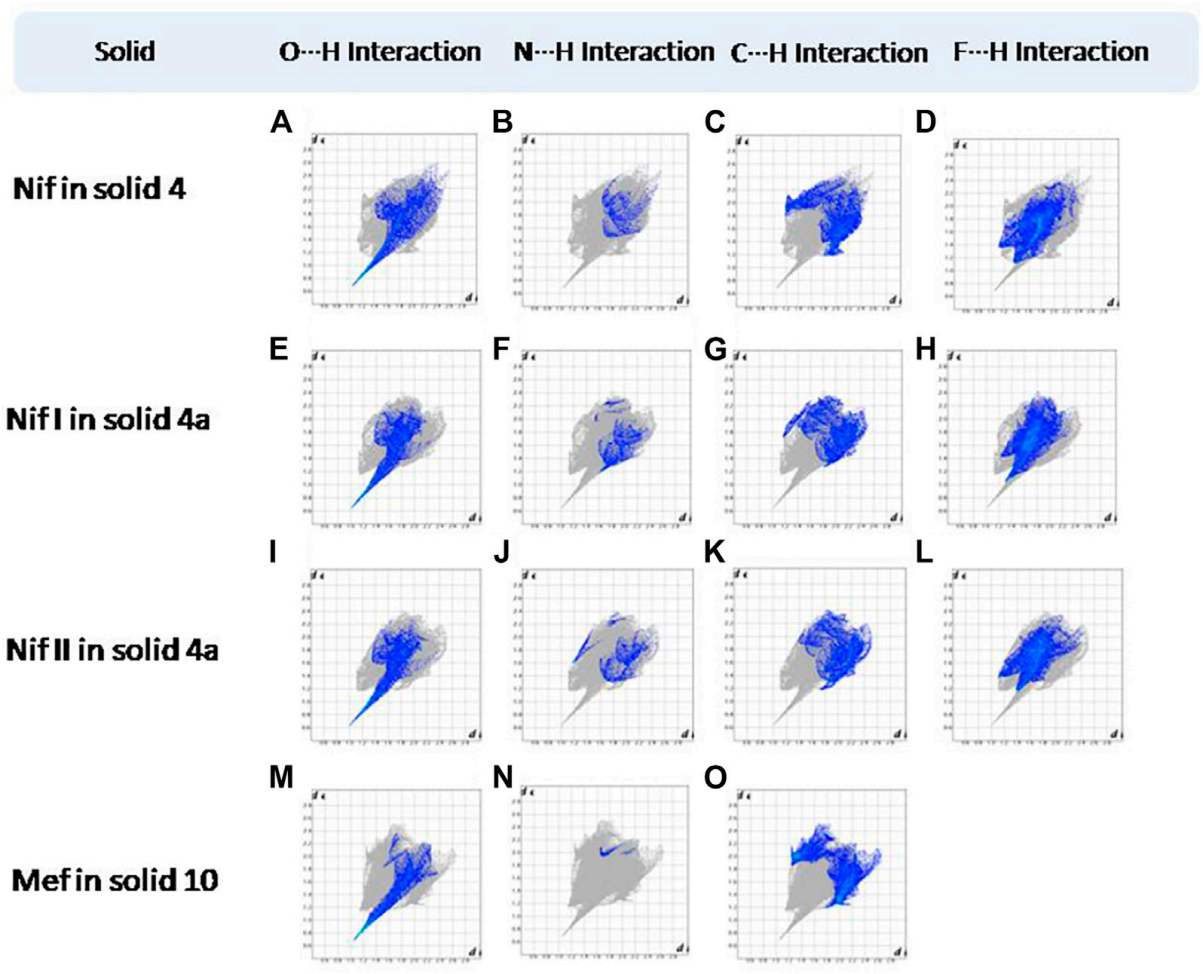
**(A–L)** Resolved fingerprint plots of Nif in **4** and Nif I &Nif II in **4a** in O∙∙∙H/ H∙∙∙O, N∙∙∙H/H∙∙∙N, C∙∙∙H/H∙∙∙C, and F∙∙∙H/H∙∙∙F interactions, respectively. **(M–O)** Resolved fingerprint plots of Mef of **10** in O∙∙∙H/H∙∙∙O, N∙∙∙H/H∙∙∙N, and C∙∙∙H/H∙∙∙C interactions, respectively.

**FIGURE 16:**
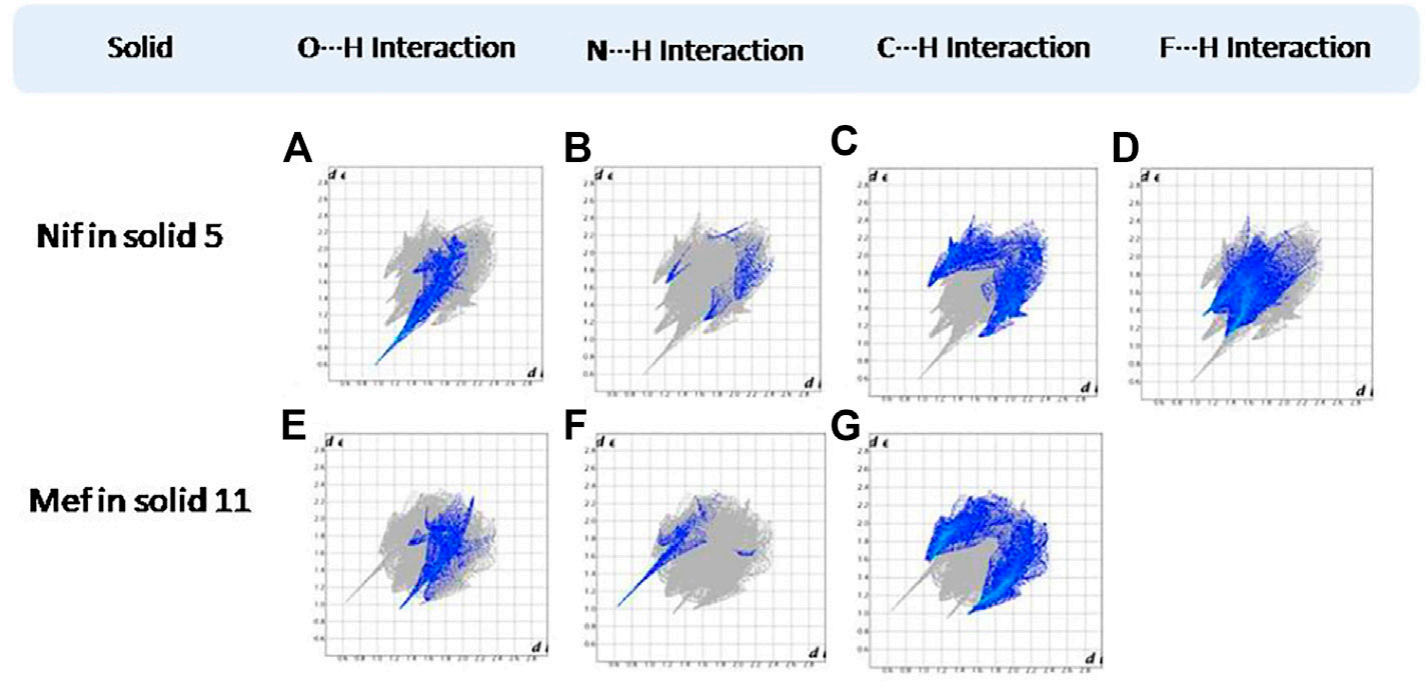
**(A–D)** Resolved fingerprint plots of Nif in **5** in O∙∙∙H/H∙∙∙O, N∙∙∙H/H∙∙∙N, C∙∙∙H/H∙∙∙C, and F∙∙∙H/H∙∙∙F interactions, respectively. **(E–G)** Resolved fingerprint plots of Mef in **11** in O∙∙∙H/ H∙∙∙O, N∙∙∙H/H∙∙∙N, and C∙∙∙H/ H∙∙∙C interactions, respectively.

**FIGURE 17:**
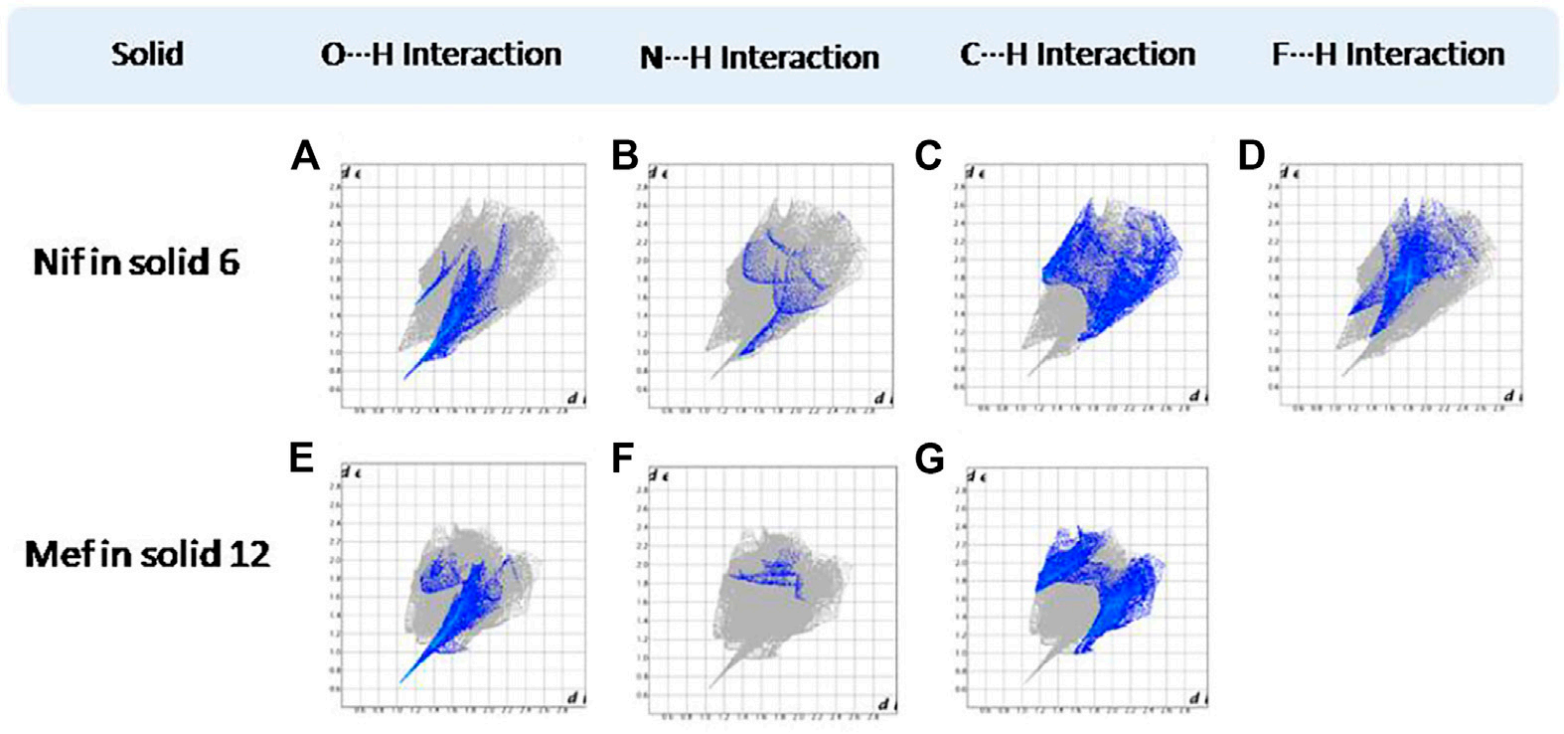
**(A–D)** Resolved fingerprint plots of Nif in **6** in O∙∙∙H/H∙∙∙O, N∙∙∙H/H∙∙∙N, C∙∙∙H/H∙∙∙C, and F∙∙∙H/H∙∙∙F interactions, respectively. **(E–G)** Resolved fingerprint plots of Mef in **12** in O∙∙∙H/ H∙∙∙O, N∙∙∙H/H∙∙∙N, and C∙∙∙H/ H∙∙∙C interactions, respectively.

Hirshfeld surfaces (HS) are frequently used to depict various types of interactions in multicomponent solids such as cocrystal, salt, hydrate or solvate and their polymorphs (Spackman and Jayatilaka, 2009). 2D finger plots derived from Hirshfeld surfaces of these solids are particularly helpful to compare intermolecular interactions that are not obvious in structurally similar compounds (Spackman and Jayatilaka, 2009). The fingerprint plots for all the solids prepared in this study were generated using di (distance from the surface to the nearest atom in the molecule) and de (distance from the surface to the nearest atom outside the molecule) as a pair of coordinates in an interval of 0.01 Å, for each surface spot resulting in two-dimensional histograms. The Hirshfeld surface resulted in a 2D plot where different colors (blue to red) indicate different frequencies of the occurrence of interaction. Hirshfeld surfaces and 2D fingerprint plots of the monomorphic Nif (NIFLUM) and dimorphic Mef (XYNAC and XYNAC02) are shown in Figures 11A–J. The HS and fingerprint plots show the variation in the environment of the molecule, which dictates the structural difference. A comparison of Nif and Mef with the coformers *bpe* and *bpee* 2D fingerprint plots (**1**, **2**, **2a, 7,** and **8**) showed similar features (Figures 12, 13). The 2D plots of **1**, **2**, **2a, 7,** and **8** show a single spike corresponding to N∙∙∙H/H∙∙∙N interaction histogram indicating the disruption of the acid-acid dimer of Nif and Mef *via* acid-pyridine synthon. Notice the absence of two spikes observed for carboxylic dimer in Figure 11. Both Nif molecules present in the asymmetric unit of **1** showed a similar histogram except for a slight difference in F∙∙∙H/H∙∙∙F interactions. The 2D fingerprint plots of Mef in **7** and **8** (Figure 13) showed exactly similar features due to the isostructural nature of **7** and **8.** A comparison of 2D plots of Nif and Mef with the conformer *bpp* (Figure 14) in **3** and **9** again showed the absence of the characteristic “two spikes” of O∙∙∙H/H∙∙∙O interaction and the presence of a single spike in N∙∙∙H/H∙∙∙N interaction due to carboxylic acid dimer disruption and acid-pyridine synthon formation. The difference in N∙∙∙H/H∙∙∙N histogram in **3** and **9** can be attributed to the difference in composition of acid and base in the solids. Solid **3** is a 2:1 acid-pyridine trimer, while **9** is an acid-pyridine dimer. The second nitrogen of *bpp* is involved in C−H∙∙∙N interaction, as is inferred by the presence of shoulder in N∙∙∙H/H∙∙∙N histogram of **9** (Figure 14G). The 2D fingerprint plots of Nif with the conformer *2ap* in solids **4** and **4a** and Mef in **10** (Figure 15) showed the absence of N∙∙∙H/H∙∙∙N interaction; the presence of one single spike in O∙∙∙H/H∙∙∙O interaction histogram specifies the O∙∙∙H interaction, which is due to proton transfer from Nif and Mef to *2ap*. The C∙∙∙H/H∙∙∙C interaction histogram of Nif (Figures 15C,G) is different in **4** and **4a** due to the difference in the extent of C−H∙∙∙π bonding in these solids. The fingerprint plots of Nif and Mef in **5** and **11** are depicted in Figure 16 and in **6** and **12** in Figure 17. The latter showed similar features of **4** and **10,** indicating the salt formation with *4ap*.

### Effect of p*K*a on Solid Forms

Crystallization of acid with a series based on structurally similar basic coformers Δp*K*_a_. The molecular salts for the carboxylic acid–pyridine reaction have a COO∙∙∙provided a platform to explore the structural difference due to H–N_arom_ heterosynthon, while the cocrystals have a COO–H∙∙∙N_arom_ heterosynthon. Formation of a cocrystal or a salt is usually predicted by an empirical indicator, the Δp*K*_a_ [Δp*K*_a_ (base) − Δp*K*_a_ (acid)] rule. (Sarma et al., 2009; Stahl et al., 2011; Lemmerer et al., 2015). As a general rule, Δp*K*_a_< 0 yields a cocrystal, while Δp*K*_a_> 3.75 leads to a salt (Childs et al., 2007; Delori et al., 2013). It is generally believed that the cocrystal or salt, or both, can appear in the domain between 0 and 4, though proton transfer is unpredictable in this region (Cruz-Cabeza, 2012). We validated the Δp*K*_a_ rule to all the multicomponent solids reported in this study as these were the products of acid and base. We found good agreement in all the cases. Solids which have Δp*K*_a_ ˃ 3.75 (**4, 4a**, **5**, **6, 10** and **12**) exclusively formed salts while solids with Δp*K*a ˂ 3.75 (**1**, **2**, **2a**, **3**, **7**, **8, 9** and **11**), resulted in the formation of a cocrystal. The data has been summarized in (Table 3).

**TABLE 3:** The outcome of solids by p*K*a difference between Nif, Mef, and coformers.

S. No.	NSAID	Coformer	ΔpKa = [pKa(base) – pKa(acid)]	Solid form type	
				Salt	Cocrystal
1	Mef	*bpee*	4.99–3.89 = 1.1	—	**8**
2	Mef	*bpe*	5.32–3.89 = 1.43	—	**7**
3	Mef	*bpp*	5.42–3.89 = 1.53	—	**9**
4	Mef	*3ap*	5.75–3.89 = 1.86	—	**11**
5	Mef	*2ap*	6.82–3.89 = 2.93	**10**	
6	Nif	*bpee*	4.99–1.89 = 3.1	—	**2, 2a**
7	Nif	*bpe*	5.32–1.89=3.43	—	**1**
8	Nif	*bpp*	5.42–1.89 = 3.53	—	**3**
9	Nif	*3ap*	5.75–1.89 = 3.86	**5**	—
10	Nif	*2ap*	6.82–1.89 = 4.93	**4, 4a**	—
11	Mef	*4ap*	8.95–3.89 = 5.06	**12**	—
12	Nif	*4ap*	8.95–1.89 = 7.06	**6**	—

### Conformational Flexibility

**FIGURE 18:**
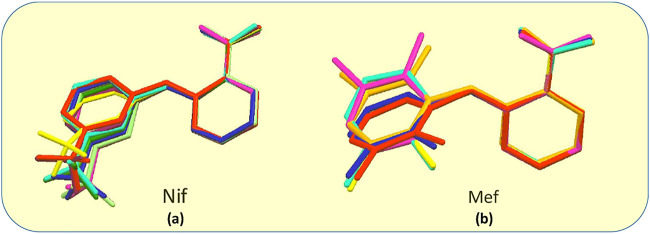
**(A)** Molecular overlay diagrams of Nif in salts/cocrystals. Color codes: Light blue‒Nif, Red‒**1**, Orange‒**2**, Yellow‒**2a**, Green‒**3**, Cyan‒**4**, Blue‒**4a**, Purple‒**5**, Magenta‒**6**. **(B)** Molecular overlay diagrams of Mef molecule in salts/cocrystals. Color codes: Light blue‒Mef I, Green‒Mef II, Red‒**7**, Orange‒**8**, Yellow‒**9**, Cyan‒**10**, Blue‒**11**, Purple‒**12**.

Fenamates, due to their free rotation around the dihedral C‒C‒N‒C bond, showed conformational polymorphs. The flexibility of free Nif/Mef in its salts/cocrystals enables the molecule to show different conformations in the solid-state. Both Nif and Mef are very flexible molecules, as can be seen from Table 4. Their different conformations are depicted in an overlayed fashion in Figure 18. Mef is dimorphic, while Nif is monomorphic. This conformational flexibility enabled Nif/Mef to form multicomponent solids with efficient hydrogen-bonding packing. The difference in torsion angles among all the solids based on Nif is evidence of its conformational flexibility. Two different Nif molecules in the asymmetric unit of **1** have almost equal torsion angles in the opposite direction. The torsion angles in Nif based solids although vary in different forms. In the case of Mef based solids, the torsion angle of **8** is close to Mef II, while in **7**, **9**, **10**, **11,** and **12,** it is different. To summarize, Nif based solids have torsion angles close to 0° or parallel, while the Mef based solids have torsion angles close to 90° or perpendicular. The ortho substitution in Mef could be the probable reason for this variation.

**TABLE 4:** Torsional angles of Nif and Mef in the salt/cocrystal form and in the free state.

Nif based solids	Nif	**1**	**2**	**2a**	**3**	**4**	**4a**	**5**	**6**
τ	–4.62	17.66, –18.70	17.98	33.03	11.90	–5.92	24.76	—	–7.45
**Mef based solids**	**Mef I**	**Mef II**	**7**	**8**	**9**	**10**	**11**	**12**	—
τ	–119.98	−80.82	90.35	−91.97	74.66	76.05	—	94.73	—

### Crystallization of Six Selected Anthranilic Acid Based NSAIDs (A) With N-Containing Coformers (B)

CSD analysis of the six selected anthranilic acid based NSAIDs (A) with N-containing co-formers (B) solids (Supplementary Scheme S1, Supplementary Tables S1, S2), led to the following conclusions based on the structural features. 4,4′-bipy is the only reported coformer that formed 2:1 cocrystal (A_2_B) with all the six acids surveyed here. Interestingly the crystal structures of all the six solids were dominated by the trimeric acid pyridine synthon (A٠٠٠B٠٠٠A) as observed in all bipyridine solids (**1**, **2**, **2a**, **3**, **7** and **8**) reported here except solid **9**. As expected, 4,4′-azopyridine is also reported to form a cocrystal with composition A_2_B. In the case of reaction with *bpee*, we obtained a cocrystal solvate concomitantly. The solid **9** (AFOPAP) showed a rare coformer-coformer homosynthon (synthon VII in Scheme 4) which could be a contributing factor towards its composition of 1:1 (Zheng et al., 2018).

Monopyridine containing solids such as acridine, methyl, chloro or amide substituted pyridine invariably led to anhydrous 1:1 cocrystals. The solvent DMF, sulfamethazine and pyridine-2-one all formed 1:1 solid. An interesting addition the cocrystal **11** formed between Mef (*p*Ka = 5.75) and *3ap*. The same coformer, however, yielded a 1:1 salt (solid **5**) with *Nif*. All coformers containing a single amino group formed only 1:1 salt with fenamic acids. Piperazine (more basic one) is the only coformer that showed both 2:1 and 1:1 salts and hydrates with the acids, *Mef*, *Tol* and *Mec*. The two cyclic tetramine and ethylenediamine gets easily diprotonated and hence formed salts of the composition, AB_2_^.^ Only three salts with refcodes BEBGOH, ZAZGEO, and JUDPUW showed a deviation in composition with the occurrence of *AB*_*2*_ for the first two and A_2_B for the last one. The three examples suggested that apart from charge balance (e.g. A^−^B^+^), microscopic stabilization of the building blocks (dimer, trimer, etc) could lead to inclusion of a neutral coformer as in AB_2_ or A_2_B or solvent in the final outcome of the crystallization.

It was observed that, statistically, ortho substituted fenamates, namely, Mefenamic acid (Mef), Tolfenamic acid (Tol), and Meclofenamic acid (Mec), showed higher tendency (39, 41 and 33%, respectively) to form solvates than Flufenamic acid (Flu) and Niflumic Acid (Nif) (18 and 10% respectively). It should be noted that Flu and Nif have higher chance of free rotation around the dihedral angle for better packing efficiency; this conformational rotation is restricted in Mef, Tol and Mec due to ortho substitution. The two acids, a sulfonic and maleic formed 1:1 salt with a protonated *Nif* with a favorable -COO٠٠٠H−N (py).

## Conclusion

The present study demonstrates the robustness of acid∙∙∙pyridine synthon to design new cocrystals, salts, salt-cocrystals, and salt hydrates based on Nif and Mef. The presence of conformational flexibility enables Nif/Mef to form multicomponent solids with efficient hydrogen-bonding packing. The Nif molecules present in the asymmetric unit of **1** showed two confirmations having almost equal torsion angles in the opposite direction. To compare, torsion angles of Nif based solids are close to 0° or parallel, while the Mef based solids have torsion angles close to 90° or perpendicular. This variation can probably be ascribed to the ortho substitution in Mef. The stoichiometry of the resulting solids is governed by conformational flexibility and/or supramolecular aggregation. The structural differences of Nif and Mef based solids are discussed via fingerprint plots generated through Hirshfeld surface analysis. The impact of Δp*K*a has been discussed and validated on Nif and Mef based solids. Apart from the two bipyridine based solids (**1** and **2**), relative solubilities of the solids showed an increase compared to their respective fenamates.

## Data Availability

The datasets presented in this study can be found in online repositories. The names of the repository/repositories and accession number(s) can be found in the article/Supplementary Material.
