# Supplementary material for: Multicomponent Solids of Niflumic and Mefenamic Acids Based on Acid-Pyridine Synthon
Source: Front Chem. 2022 Mar 31;10:729608. doi: 10.3389/fchem.2022.729608 (PMC9009247; doi:10.3389/fchem.2022.729608)
Supplement: Supplementary file 1 [file DataSheet3.docx]

**Multicomponent solids of Niflumic and Mefenamic acid based on acid-pyridine synthon**

Vineet Kumar, ^†^ Pramod Kumar Goswami, ^†^ Balendra, ^†#^ Shailabh Tewari, ^†^ and Arunachalam Ramanan^* †^

^†^Department of Chemistry, Indian Institute of Technology Delhi, Hauz Khas, New Delhi-110016, India

# Department of Chemistry, Sri Venkateswara College, University of Delhi, Delhi-110007, India

E-mail : [aramanan@chemistry.iitd.ac.in](mailto:aramanan@chemistry.iitd.ac.in) (A.R.), Tel : +91-11-26591507. Fax: +91-11-26581102.

**Scheme S1.** Polymorphic forms of fenamate derivatives. The substitutions on the phenyl ring are critical in governing the relative orientation of the two aromatic rings which in turn impacts the number of polymorphic forms as well as how it interacts with a coformer under given conditions. Here, Ffa = Flufenamic Acid, Ta = Tolfenamic Acid, 2-Pna = 2-(phynylamino)nicotinic acid, 2-(2-M3ca) = 2-(2-Methyl-3-chloroanilino)nicotinic acid, 2-Mana = 2-(Mesitylamino)nicotinic acid, Tna = 2-(phynylamino)nicotinic acid, and Mef = Mefenamic Acid.

Scheme S2. Number of multicomponenrt solids in CCDC version 5.43 (November 2021) with different fenamates.

Table S1. Literature analysis of multicomponent solids based on fenamates in CCDC version 5.43 (November 2021). Compositions depicted in blue are reported as cocrystals while that in red are reported as salts. The CCDC code mentioned is hyperlinked to the [CCDC webpage](https://www.ccdc.cam.ac.uk/structures/?ccdc-check=2dbbdb1af729caf6f19d64fd625c52ed) for the corresponding structure. Solids reported in this study are labelled with their respective number in the present study and have been coloured green and highlighted. The *p*Ka values for fenamates as well as coformers have been calculated using ChemAxon software.

| **Base Moiety** | **** | | | | |  |
| --- | --- | --- | --- | --- | --- | --- |
| **R** |  |  |  |  |  |  |
| **Coformers** | **Fenamic Acid**  **(H*fen*)** | **Mefenamic Acid**  **(H*mef*)** | **Tolfenamic Acid**  **(H*tol*)** | **Meclofenamic Acid (H*mec*)** | **Flufenamic Acid**  **(H*flu*)** | **Niflumic Acid**  **(H*nif*)** |
|  | (p*K*a =4.91) | (p*K*a =4.94) | (p*K*a =4.77) | (p*K*a =4.59) | (p*K*a =4.91) | (p*K*a =2.63) |
| 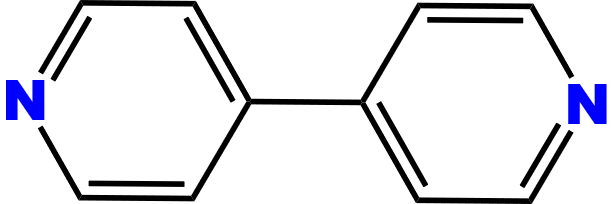  *bipy* = 4,4'-bipyridine  1^st^ p*K*a=5.25  2^nd^ p*K*a = 4.44 | **(H*fen)_2_·(bipy)***  [PEFCAG01](https://www.ccdc.cam.ac.uk/structures/Search?Ccdcid=PEFCAG01&DatabaseToSearch=Published)  (Surov et al. 2014)  *P*$\bar{1}$ *a*=7.760Å  *b*=9.757Å  *c*=20.063Å α*=*98.29°  β=92.42°  γ*=*104.59° V=1450.01Å^3^ Z*=*2 | ***(*H*mef)_2_·(bipy)***  [XOWKEB](https://www.ccdc.cam.ac.uk/structures/Search?Ccdcid=XOWKEB&DatabaseToSearch=Published)  (Surov et al. 2014; Wittering et al. 2015)  *P*$\bar{1}$ *a=*7.308Å  *b=*8.669Å  *c=*13.632Å  α=106.13°  β=99.42°  γ=98.75° V*=*800.46Å^3^ Z*=*1 | ***(*H*tol)_2_·(bipy)***  [XOWKAX01](https://www.ccdc.cam.ac.uk/structures/Search?Ccdcid=XOWKAX01&DatabaseToSearch=Published)  (Wittering et al. 2015)  *P*2_1_/*c* *a=*4.708Å  *b=*45.219Å  *c=*7.918Å  β=106.89° V*=*1613.12Å^3^ Z*=*2 | ***(*H*mec)_2_·(bipy)***  [SAXPEO](https://www.ccdc.cam.ac.uk/structures/Search?Ccdcid=SAXPEO&DatabaseToSearch=Published)  (Sanphui, Bolla, and Nangia 2012)  *P*2_1_/*c* *a=*7.418Å  *b=*8.214Å  *c=*28.771Å  β=97.09°  V*=*1739.64Å^3^ Z*=*4 | ***(*H*flu)_2_·(bipy)***  [ZIQFEM02](https://www.ccdc.cam.ac.uk/structures/Search?Ccdcid=ZIQFEM02&DatabaseToSearch=Published)  (Wittering et al. 2015)  *P*$\bar{1}$ *a=*9.829Å  *b=*10.383Å  *c=*24.990Å  α=84.68°  β=81.33°  γ=71.77° V*=*2392.05Å^3^ Z*=*3 | ***(*H*nif)_2_·(bipy)***  [XOWKIF](https://www.ccdc.cam.ac.uk/structures/Search?Ccdcid=XOWKIF&DatabaseToSearch=Published)  (Surov et al. 2015)  *C*2/*c* *a=26.001*Å  *b=6.739*Å  *c=19.854*Å  β=111.38°  V*=3239.37*Å^3^ Z*=4* |
| *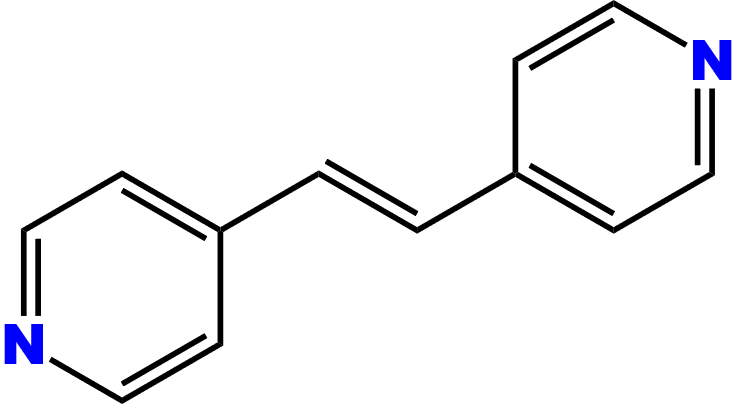*  *bpee* = 1,2-bis(4-pyridyl)ethylene  1st p*K*a =5.59  2nd p*K*a =4.99 |  | **Solid 8**  ***(*H*mef)_2_·(bpee)***  [AFONIV](https://www.ccdc.cam.ac.uk/structures/Search?Ccdcid=AFONIV&DatabaseToSearch=Published)  (Zheng et al. 2018)  *P*$\bar{1}$ *a=*7.819Å  *b=*8.615Å  *c=*12.912Å  α=98.21°  β=99.51°  γ=90.02° V*=*848.06Å^3^ Z*=*1 |  |  |  | **Solid 2 *(*H*nif)_2_·(bpee)*** |
|  |  |  |  |  |  | **Solid 2a *(*H*nif)_2_·(bpee)***  ***·1,4-dioxane*** |
| *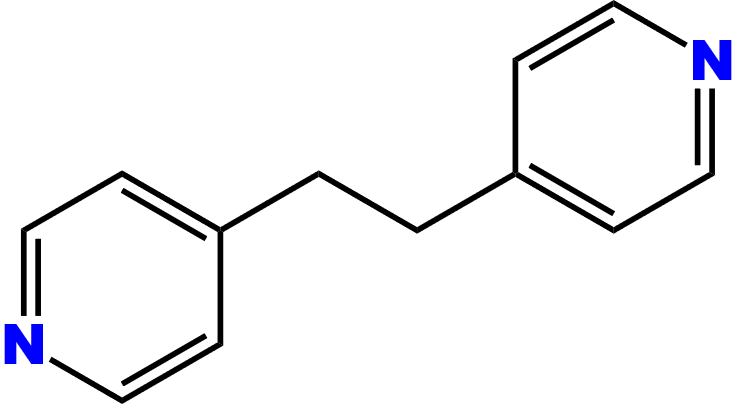*  *bpe* = 1,2-bis(4-pyridyl)ethane  1st p*K*a =5.98  2nd p*K*a =5.38 |  | **Solid 7**  ***(*H*mef)_2_·(bpe)***  [AFONOB](https://www.ccdc.cam.ac.uk/structures/Search?Ccdcid=AFONOB&DatabaseToSearch=Published)  (Zheng et al. 2018)  *P*$\bar{1}$ *a=*7.451Å  *b=*7.835Å  *c=*16.895Å  α=94.3°  β=93.16°  γ=117.89° V*=*864.61Å^3^ Z*=*1 |  |  |  | **Solid 1 *(*H*nif)_2_·(bpe)*** |
| *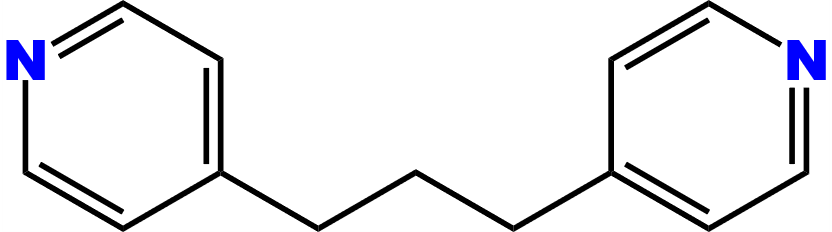*  *bpp* = 4,4'-(propane-1,3-diyl)dipyridine  1st p*K*a =6.02  2nd p*K*a =5.42 |  | **Solid 9**  ***(*H*mef)·(bpp)***  [AFOPAP](https://www.ccdc.cam.ac.uk/structures/Search?Ccdcid=AFOPAP&DatabaseToSearch=Published)  (Zheng et al. 2018)  *P*$\bar{1}$ *a=*7.53Å  *b=*7.84Å  *c=*20.38Å  α=88.77°  β=85.38°  γ=79.99° V*=*1176.22Å^3^ Z*=*2 |  |  |  | **Solid 3 *(*H*nif)_2_·(bpp)*** |
| *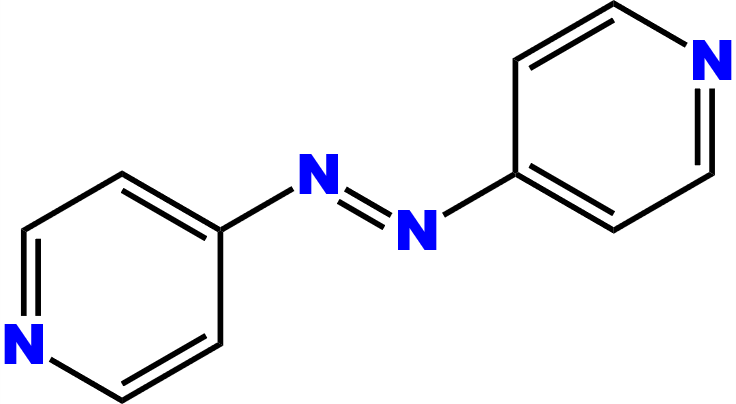*  *azo* = 4,4'-azopyridine  1st p*K*a =3.43  2nd p*K*a =2.82 |  | ***(*H*mef)_2_·(azo)***  [AFONUH](https://www.ccdc.cam.ac.uk/structures/Search?Ccdcid=AFONUH&DatabaseToSearch=Published)  (Zheng et al. 2018)  *P*$\bar{1}$ *a=*7.549Å  *b=*8.736Å  *c=* 13.095Å  α=100.62°  β=99.73°  γ=93.92° V*=*832.17Å^3^ Z*=*1 |  |  |  |  |
| *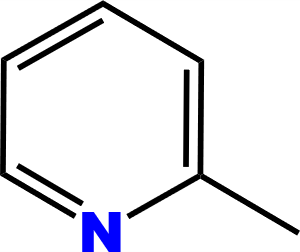*  *2mp* = 2-methylpyridine  p*K*a =5.81 |  | ***(*H*mef)·(2mp)***  [UZUYIZ01](https://www.ccdc.cam.ac.uk/structures/Search?Ccdcid=UZUYIZ01&DatabaseToSearch=Published)  (Bouanga Boudiombo and Jacobs 2016)  *P*2_1_/*n* *a=*7.785Å  *b=*8.185Å  *c=*27.953Å  β=96.87° V*=*1768.73Å^3^ Z*=*4 | ***(*H*tol)·(2mp)***  [UZUZIA](https://www.ccdc.cam.ac.uk/structures/Search?Ccdcid=UZUZIA&DatabaseToSearch=Published)  (Bouanga Boudiombo and Jacobs 2016)  *P*2_1_/*n* *a=*7.758Å  *b=*8.069Å  *c=*28.056Å  β=97.291° V*=*1742.39Å^3^ Z*=*4 |  |  |  |
| *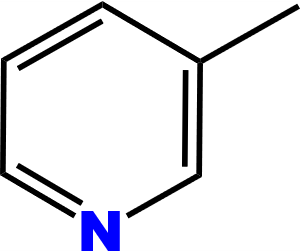*  *3mp* = 3-methylpyridine  p*K*a =5.63 |  | ***(*H*mef)·(3mp)***  [UZUYUL](https://www.ccdc.cam.ac.uk/structures/Search?Ccdcid=UZUYUL&DatabaseToSearch=Published)  (Bouanga Boudiombo and Jacobs 2016)  *P*$\bar{1}$ *a=*7.673Å  *b=*7.759Å  *c=*16.272Å  α=78.95°  β=83.67°  γ=65.09° V*=*862.11Å^3^ Z*=*2 | ***(*H*tol)·(3mp)***  [UZUZOG](https://www.ccdc.cam.ac.uk/structures/Search?Ccdcid=UZUZOG&DatabaseToSearch=Published)  (Bouanga Boudiombo and Jacobs 2016)  *Pbca* *a=*7.806Å  *b=*13.987Å  *c=*32.185Å  V*=*3514.44Å^3^ Z*=*8 |  |  |  |
| *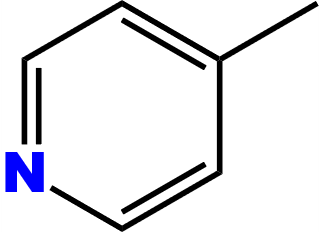*  *4mp* = 4-methylpyridine  p*K*a =5.85 |  | ***(*H*mef)·(4mp)***  [UZUZEW](https://www.ccdc.cam.ac.uk/structures/Search?Ccdcid=UZUZEW&DatabaseToSearch=Published)  (Bouanga Boudiombo and Jacobs 2016)  *P*$\bar{1}$ *a=*7.527Å  *b=*7.570Å  *c=*15.661Å  α=88.11°  β=78.41°  γ=89.21° V*=*873.86Å^3^ Z*=*2 |  |  |  |  |
| *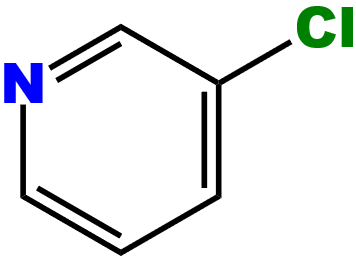*  *3chp* = 3-chloropyridine  p*K*a =3.31 |  | ***(*H*mef)·(3chp)***  [UZUZAS](https://www.ccdc.cam.ac.uk/structures/Search?Ccdcid=UZUZAS&DatabaseToSearch=Published)  (Bouanga Boudiombo and Jacobs 2016)  *P*$\bar{1}$ *a=*7.671Å  *b=*7.744Å  *c=*16.056Å  α=80.06°  β=84.88°  γ=65.52° V*=*854.82Å^3^ Z*=*2 |  |  |  |  |
| 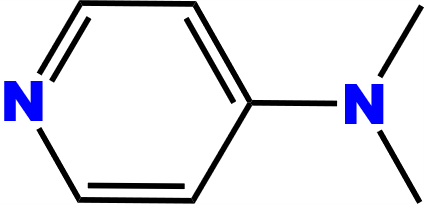  *map* = 4-(dimethylamino)pyridine  p*K*a =8.78 |  | ***(mef)^-^(*H*map)^+^***  [JUDPIK](https://www.ccdc.cam.ac.uk/structures/Search?Ccdcid=JUDPIK&DatabaseToSearch=Published)  (Ranjan et al. 2020)  *P*$\bar{1}$ *a=*7.757Å  *b=*9.472Å  *c=*13.307Å  α=87.51°  β=78.59°  γ=74.17° V*=*922.20Å^3^ Z*=*2 | ***(*H*tol)_2_·(map)***  [JUDNUU](https://www.ccdc.cam.ac.uk/structures/Search?Ccdcid=JUDNUU&DatabaseToSearch=Published)  (Ranjan et al. 2020)  *P*$\bar{1}$ *a=*10.886Å  *b=*12.271Å  *c=*13.781Å  α=106.97°  β=105.78°  γ=103.32° V*=*1595.53Å^3^ Z*=*2 |  |  |  |
|  |  | ***(mef)^-^(*H*map)^+^***  [JUDPUW](https://www.ccdc.cam.ac.uk/structures/Search?Ccdcid=JUDPUW&DatabaseToSearch=Published)  (Ranjan et al. 2020)  *P*$\bar{1}$ *a=*10.767Å  *b=*11.967Å  *c=*13.786Å  α=106.15°  β=105.84°  γ=103.49° V*=*1546.91Å^3^ Z*=*2 | ***(tol)^-^(*H*map)^+^***  [JUDNII](https://www.ccdc.cam.ac.uk/structures/Search?Ccdcid=JUDNII&DatabaseToSearch=Published)  (Ranjan et al. 2020)  *P*$\bar{1}$ *a=*7.929Å  *b=*9.322Å  *c=*13.586Å  α=87.77°  β=76.93°  γ=76.03° V*=*949.23Å^3^ Z*=*2 |  |  |  |
|  |  | ***(mef)^-^(*H*map)^+^·H_2_O***  [TAMCIW01](https://www.ccdc.cam.ac.uk/structures/Search?Ccdcid=TAMCIW01&DatabaseToSearch=Published)  (Nechipadappu and Trivedi 2017; Ranjan et al. 2020)  *P*$\bar{1}$ *a=*7.8149Å  *b=*8.1357Å  *c=*16.3674Å  α=79.58°  β=81.33°  γ=89.95° V*=*1011.43Å^3^ Z*=*2 | ***(tol)^-^(*H*map)^+^·H_2_O***  TAMCUI  (Nechipadappu and Trivedi 2017; Ranjan et al. 2020)  *P*$\bar{1}$ *a=*7.848Å  *b=*8.103Å  *c=*16.309Å  α=100.58°  β=98.03°  γ=90.38° V*=*1008.98Å^3^ Z*=*2 |  |  |  |
| *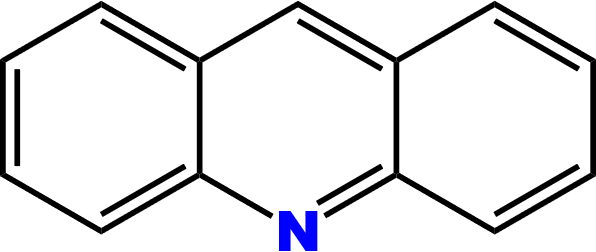*  *acd* = acridine  p*K*a =6.95 | **(H*fen)·(acd)***  [OYEBAX](https://www.ccdc.cam.ac.uk/structures/Search?Ccdcid=OYEBAX&DatabaseToSearch=Published)  (Jerzykiewicz, Sroka, and Majerz 2016)  *P*2_1_/*c*  *a*=9.184Å  *b*=17.224Å  *c*=13.116Å β=106.08° V*=*1993.58Å^3^ Z*=*4 |  |  |  |  |  |
| *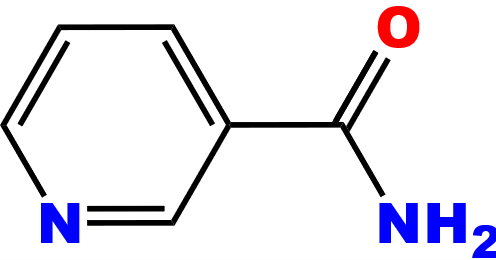*  *nic* = nicotinamide  p*K*a =3.63 |  | ***(*H*mef)·(nic)_2_***  [EXAQOK](https://www.ccdc.cam.ac.uk/structures/Search?Ccdcid=EXAQOK&DatabaseToSearch=Published)  (Fábián et al. 2011; Vaksler et al. 2021)  *P*$\bar{1}$ *a=*4.064Å  *b=*12.509Å  *c=*24.088Å  α=99.89°  β=90.73°  γ=92.43° V*=*1205.15Å^3^ Z*=*2 | ***(*H*tol)·(nic)_2_***  [EXAQIE](https://www.ccdc.cam.ac.uk/structures/Search?Ccdcid=EXAQIE&DatabaseToSearch=Published)  (Fábián et al. 2011)  *P*$\bar{1}$ *a=*4.006Å  *b=*12.554Å  *c=*24.126Å  α=100.32°  β=90.40°  γ=92.53° V*=*1192.55Å^3^ Z*=*2 |  | ***(*H*flu)·(nic)***  [EXAQAW](https://www.ccdc.cam.ac.uk/structures/Search?Ccdcid=EXAQAW&DatabaseToSearch=Published)  (Fábián et al. 2011)  *P*2_1_/*c* *a=*5.105Å  *b=*15.961Å  *c=*22.119Å  β=90.47° V*=*1802.35Å^3^ Z*=*4 | ***(*H*nif)·(nic)***  [EXAQEA](https://www.ccdc.cam.ac.uk/structures/Search?Ccdcid=EXAQEA&DatabaseToSearch=Published)  (Fábián et al. 2011)  *P*2_1_/*c* *a=15.152*Å  *b=5.060*Å  *c=24.674*Å  β=112°  V*=1751.72*Å^3^ Z*=4* |
| 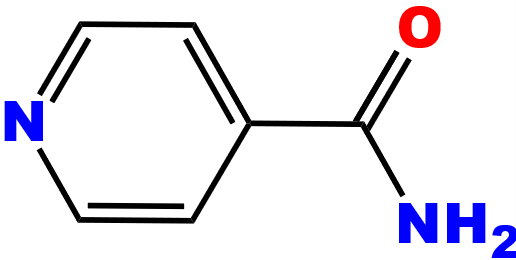  *inic* = isonicotinamide  p*K*a =3.45 |  |  |  | ***(*H*mec)·(inic)***  [SAXPAK](https://www.ccdc.cam.ac.uk/structures/Search?Ccdcid=SAXPAK&DatabaseToSearch=Published)  (Sanphui, Bolla, and Nangia 2012)  *P*2_1_/*c* *a=*7.532Å  *b=*31.313Å  *c=*9.601Å  β=121.44°  V*=*1932.14Å^3^ Z*=*4 |  |  |
| *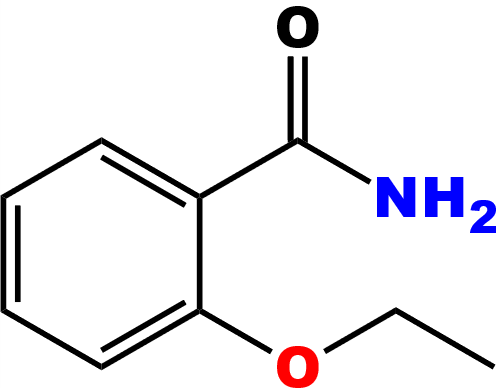*  *etb* = 2-ethoxybenzamide  p*K*a = -0.93 |  |  |  |  | ***(*H*flu)·(etb)***  [FAQXAZ](https://www.ccdc.cam.ac.uk/structures/Search?Ccdcid=FAQXAZ&DatabaseToSearch=Published)  (Nechipadappu, Tekuri, and Trivedi 2017)  *P*$\bar{1}$ *a=*7.628Å  *b=*8.807Å  *c=*17.427Å  α=103.78°  β=94.74°  γ=102.94° V*=*1096.74Å^3^ Z*=*2 |  |
| *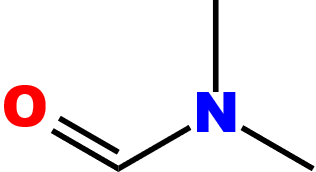*  *dmf* = N, N-dimethylformamide  p*K*a =-1.26 |  | ***(*H*mef)·(dmf)***  [ZAZGAK](https://www.ccdc.cam.ac.uk/structures/Search?Ccdcid=ZAZGAK&DatabaseToSearch=Published)  (SeethaLekshmi and Guru Row 2012)  *P*$\bar{1}$ *a=*7.473Å  *b=*9.559Å  *c=*13.306Å  α=105.07°  β=103.78°  γ=103.41° V*=*846.51Å^3^ Z*=*2 | ***(*H*tol)·(dmf)***  [JUDPEG](https://www.ccdc.cam.ac.uk/structures/Search?Ccdcid=JUDPEG&DatabaseToSearch=Published)  (Ranjan et al. 2020)  *P*$\bar{1}$ *a=*10.480Å  *b=*11.842Å  *c=*13.331Å  α=94.33°  β=95.88°  γ=102.87° V*=*1596.16Å^3^ Z*=*4 |  |  |  |
| *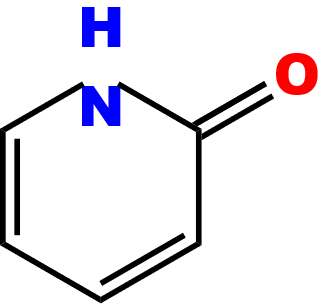*  *pyo* = pyridin-2-one  p*K*a = 11.40 |  |  |  |  | ***(*H*flu)·(pyo)***  [ZIQFAI](https://www.ccdc.cam.ac.uk/structures/Search?Ccdcid=ZIQFAI&DatabaseToSearch=Published)  (Aitipamula et al. 2014)  *P*$\bar{1}$ *a=*7.562Å  *b=*7.645Å  *c=*14.552Å  α=90.45°  β=92.14°  γ=95.81° V*=*836.39Å^3^ Z*=*2 | ***(*H*nif)·(pyo)***  [RIQXEX](https://www.ccdc.cam.ac.uk/structures/Search?Ccdcid=RIQXEX&DatabaseToSearch=Published)  (Mittapalli et al. 2019)  *P*$\bar{1}$ *a=7.207*Å  *b=10.314*Å  *c=11.889*Å  α=100.34°  β=101.77°  γ=90.48° V*=850.17*Å^3^ Z*=2* |
| *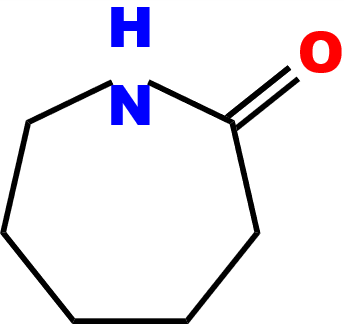*  *azp* = azepan-2-one  p*K*a =-1.44 |  |  |  |  |  | ***(*H*nif)·(azp)***  [RIQXAT](https://www.ccdc.cam.ac.uk/structures/Search?Ccdcid=RIQXAT&DatabaseToSearch=Published)  (Mittapalli et al. 2019)  *P*$\bar{1}$ *a=5.451*Å  *b=10.478*Å  *c=16.891*Å  α=89.61°  β=85.51°  γ=86.69° V*=960.14*Å^3^ Z*=2* |
| 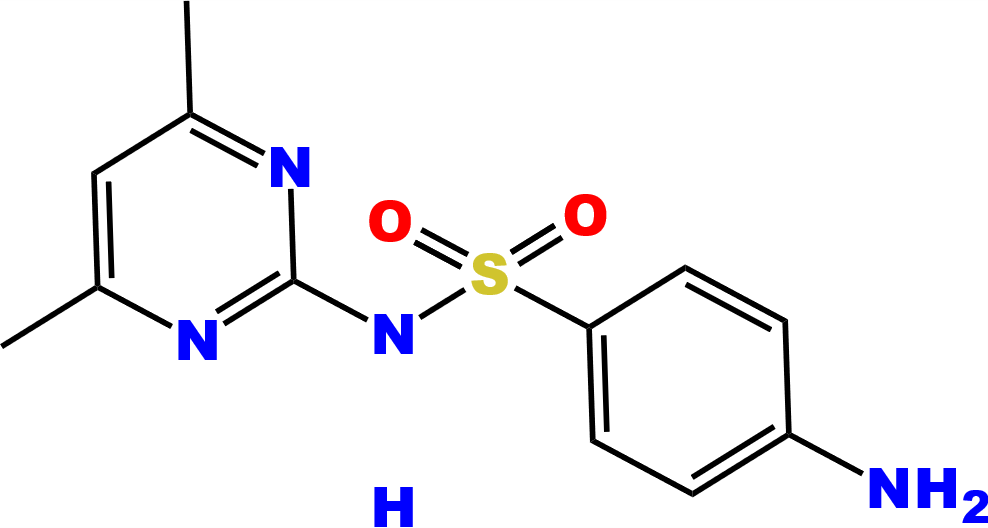  smz = sulfamethazine  p*K*a =2 |  |  |  |  | ***(*H*flu)·(smz)***  [DARNOC01](https://www.ccdc.cam.ac.uk/structures/Search?Ccdcid=DARNOC01&DatabaseToSearch=Published)  (Bhattacharya et al. 2020)  *Pbca* *a=*15.931Å  *b=*14.710Å  *c=*21.885Å V*=*5129.08Å^3^ Z*=*8 | ***(*H*nif)·(smz)***  [DARGEL](https://www.ccdc.cam.ac.uk/structures/Search?Ccdcid=DARGEL&DatabaseToSearch=Published)  (Kumar et al. 2017; Bhattacharya et al. 2020)  *Pbca* *a=15.840*Å  *b=14.613*Å  *c=21.829*Å  V*=5052.76*Å^3^ Z*=8* |
| *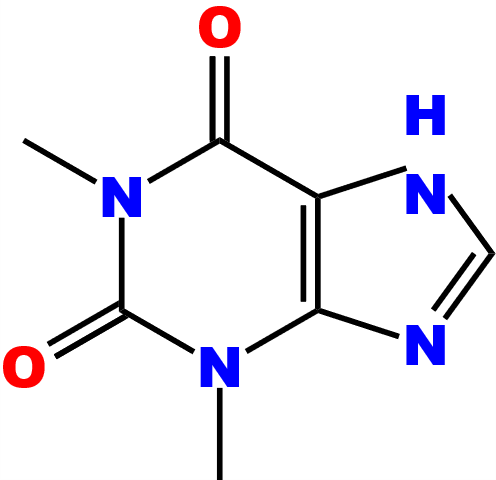*  *mhp* = 1,3-dimethyl-3,7-dihydro-1H-purine-2,6-dione  p*K*a =-0.78 |  |  |  |  | ***(*H*flu)·(mhp)***  [ZIQDUA](https://www.ccdc.cam.ac.uk/structures/Search?Ccdcid=ZIQDUA&DatabaseToSearch=Published)  (Aitipamula et al. 2014)  *P*$\bar{1}$ *a=*6.993Å  *b=*10.127Å  *c=*15.112Å  α=95.46°  β=96.7°  γ=101.17°  V*=*1035.13Å^3^ Z*=*2 |  |
| *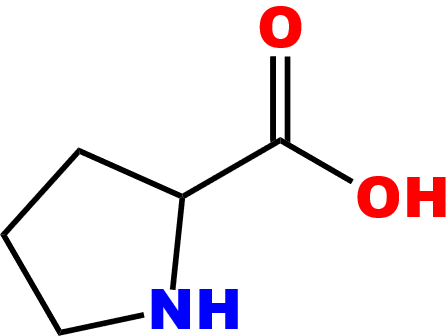*  *pro* = L-proline  p*K*a =1.94,11.33  Zwiterion |  |  |  |  |  | ***(*H*nif)·(pro)***  [LIRSEN](https://www.ccdc.cam.ac.uk/structures/Search?Ccdcid=LIRSEN&DatabaseToSearch=Published)  (Surov et al. 2018) *P*2_1_  *a=11.807*Å  *b=5.812*Å  *c=13.348*Å  β=97.84°  V*=907.51*Å^3^ Z*=2* |
| *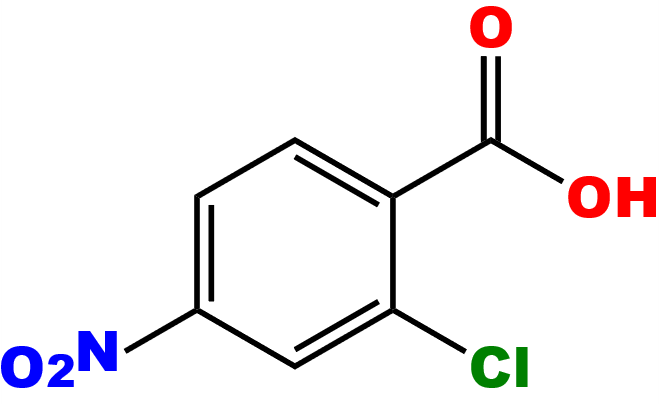*  *cnba* = 2-chloro-4-nitrobenzoic acid  p*K*a =2.46 |  |  |  |  | ***(*H*flu)·(cnba)***  [FAQWUS](https://www.ccdc.cam.ac.uk/structures/Search?Ccdcid=FAQWUS&DatabaseToSearch=Published)  (Nechipadappu, Tekuri, and Trivedi 2017)  *P*2_1_/*c* *a=*7.633Å  *b=*32.137Å  *c=*8.440Å  β=95.17° V*=*2062.16Å^3^ Z*=*4 |  |
| *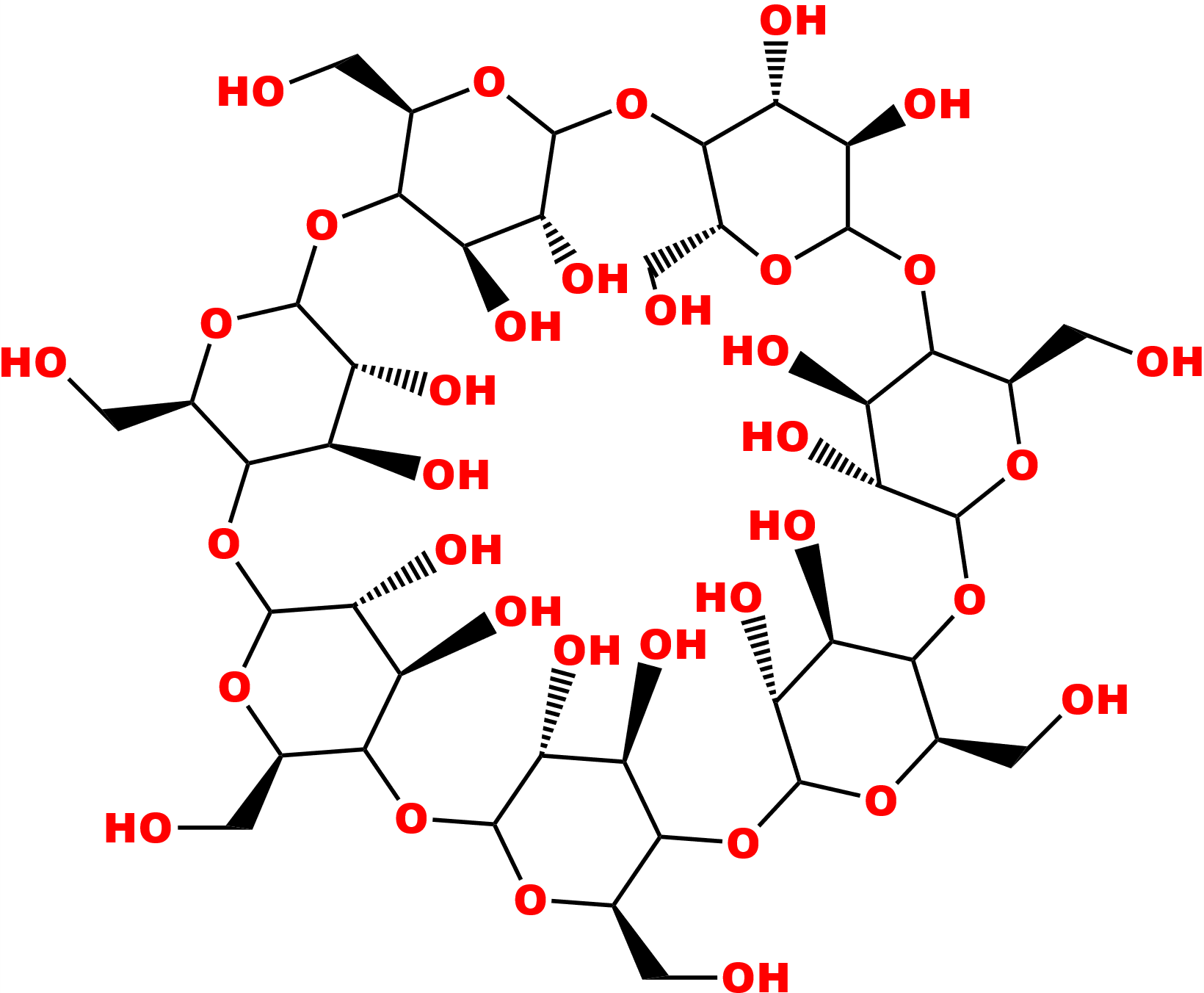*  *cyd* = β-Cyclodextrin |  | ***(*H*mef)·(cyd)***  [MUPNEQ01](https://www.ccdc.cam.ac.uk/structures/Search?Ccdcid=MUPNEQ&DatabaseToSearch=Published)  (Pop et al. 2002)  *P*2_1_ *a=*15.479Å  *b=*25.589Å  *c=*9.297Å  β=98.97° V*=*3637.68Å^3^ Z*=*2 |  |  |  |  |
| *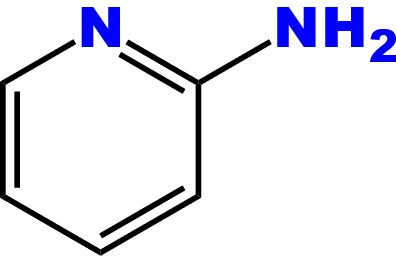*  *2ap* ***=*** 2-aminopyridine  p*K*a =6.84 |  | **Solid 10**  ***(mef)^-^·(H2ap)^+^·H2O*** |  | ***(mec)^-^(*H*2ap)^+^***  [SAXQIT](https://www.ccdc.cam.ac.uk/structures/Search?Ccdcid=SAXQIT&DatabaseToSearch=Published)  (Sanphui, Bolla, and Nangia 2012)  *P*2_1_/*n* *a=*14.675Å  *b=*7.132Å  *c=*18.605Å  α=90°  β=110.73°  γ=90° V*=*1821.12Å^3^ Z*=*4 |  | **Solid 4**  ***(nif)^-^(*H*2ap)^+^*** |
|  |  |  |  |  |  | **Solid 4a**  ***(nif)^-^(*H*2ap)^+^· 1,4-dioxane*** |
| *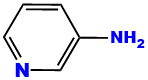*  *3ap= 3-aminopyridine*  p*K*a=5.75 |  | **Solid 11**  **(mef)·(3ap)** |  |  |  | **Solid 5**  ***(nif)^-^(*H*3ap)^+^*** |
| 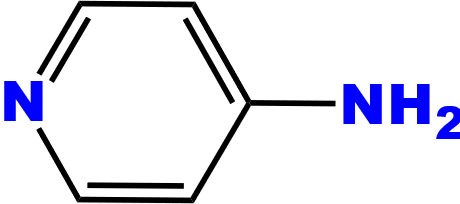  *4ap* = 4-aminopyridine  p*K*a=8.95 |  | **Solid 12**  ***(mef)^-^(*H*4ap)^+^·H_2_O***  [TAMCES](https://www.ccdc.cam.ac.uk/structures/Search?Ccdcid=TAMCES&DatabaseToSearch=Published)  (Nechipadappu and Trivedi 2017)  *P*2_1_/*n* *a=*7.760Å  *b=*8.352Å  *c=*28.623Å  β=95.31° V*=*1847.21Å^3^ Z*=*4 | ***(tol)^-^(*H*4ap)^+^·H_2_O***  [TAMCOC](https://www.ccdc.cam.ac.uk/structures/Search?Ccdcid=TAMCOC&DatabaseToSearch=Published)  (Nechipadappu and Trivedi 2017)  *P*2_1_/*n* *a=*7.774Å  *b=*8.326Å  *c=*28.369Å  β=95.17° V*=*1829.04Å^3^ Z*=*4 | ***(mec)^-^(*H*4ap)^+^·H_2_O***  [SAXQOZ](https://www.ccdc.cam.ac.uk/structures/Search?Ccdcid=SAXQOZ&DatabaseToSearch=Published)  (Sanphui, Bolla, and Nangia 2012)  *P*2_1_/*c* *a=*16.227Å  *b=*8.477Å  *c=*15.686Å  α=90°  β=116.18°  γ=90° V*=*1936.35Å^3^ Z*=*4 |  | **Solid 6**  ***(nif)^-^(*H*4ap)^+^*** |
| *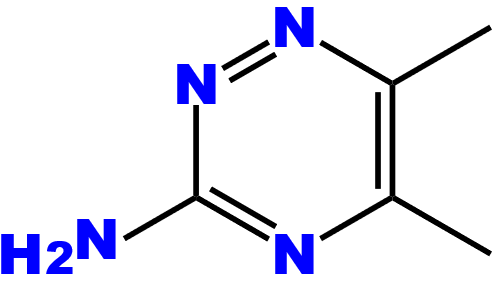*  *trz =* 3-amino-5,6-dimethyl-1,2,4-triazine  p*K*a=4.55 | ***(fen)^-^(*H*trz)^+^(trz)***  [BEBGOH](https://www.ccdc.cam.ac.uk/structures/Search?Ccdcid=BEBGOH&DatabaseToSearch=Published)  (Sangeetha et al. 2017)  (2:1)  *P*$\bar{1}$ *a*=7.501Å  *b*=7.666Å  *c*=21.433Å α*=*93.74°  β=94.77°  γ*=*106.19°  V=1174.52Å^3^ Z=2 |  |  |  |  |  |
| 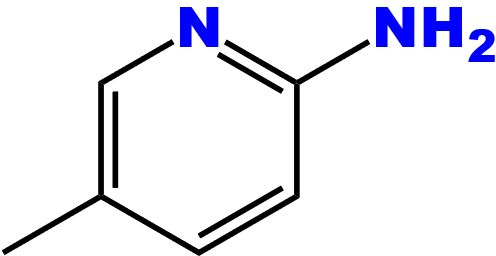  *mpd* = 2-amino-5-methylpyridine  p*K*a=7.22 | ***(fen)^-^(*H*mpd)^+^***  [OZEXAU](https://www.ccdc.cam.ac.uk/structures/Search?Ccdcid=OZEXAU&DatabaseToSearch=Published)  (Farhadikoutenaei and Ibrahim 2016)  *P*2_1_/*c* *a*=17.379Å  *b*=6.672Å  *c*=14.387Å β=92.15° V*=*1667.32Å^3^ Z*=*4 |  |  |  |  |  |
| *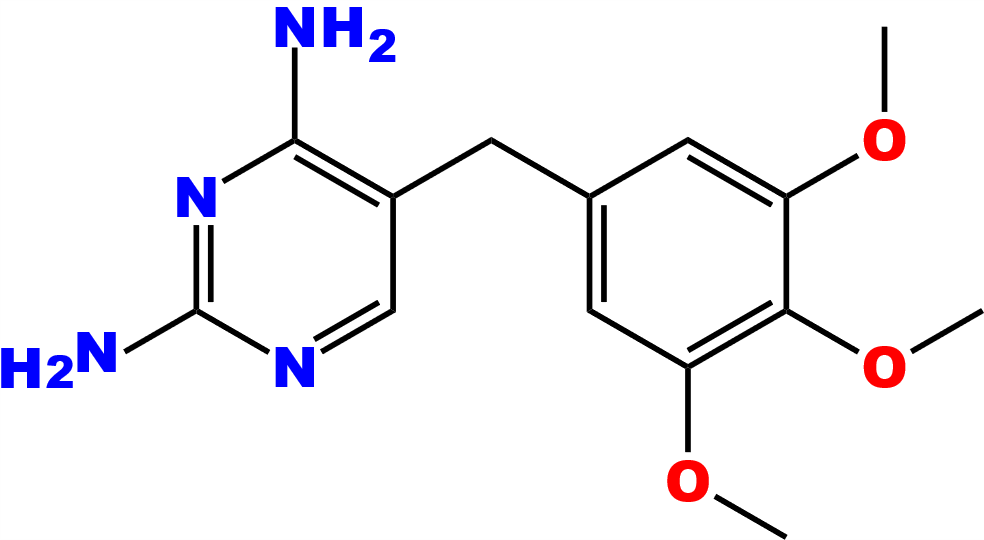*  *tmp* ***=*** trimethoprim  p*K*a=7.16 |  | ***(*H*mef) ^-^(tmp) ^+^·H_2_O***  [PORVEA](https://www.ccdc.cam.ac.uk/structures/Search?Ccdcid=PORVEA&DatabaseToSearch=Published)  (Bhattacharya et al. 2020)  *P*$\bar{1}$ *a=*9.360Å  *b=*12.251Å  *c=*13.876Å  α=98.25°  β=107.41°  γ=108.56° V*=*1388.40Å^3^ Z*=*2 | ***(tol)^-^(*H*tmp)^+^·H_2_O***  [PORVAW](https://www.ccdc.cam.ac.uk/structures/Search?Ccdcid=PORVAW&DatabaseToSearch=Published)  (Bhattacharya et al. 2020)  *P*$\bar{1}$ *a=*9.351Å  *b=*12.216Å  *c=*13.934Å  α=98.43°  β=108.31°  γ=108.26° V*=*1381.14Å^3^Z*=*2 |  | ***(flu)^-^(*H*tmp)^+.^H2O***  [PORTUO](https://www.ccdc.cam.ac.uk/structures/Search?Ccdcid=PORTUO&DatabaseToSearch=Published)  (Bhattacharya et al. 2020)  *P*$\bar{1}$ *a=*9.605Å  *b=*12.238Å  *c=*13.671Å  α=99.63°  β=106.04°  γ=109.05° V*=*1400.04Å^3^ Z*=*2 |  |
| *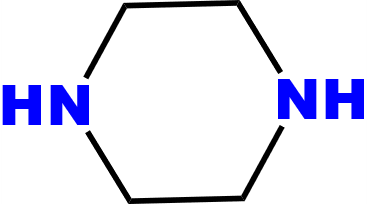*  *pip* *=* piperazine  1st p*K*a=9.56  2nd p*K*a=5.18 |  | ***(mef)^-^(*H*pip)^+^***  [SOGVIW](https://www.ccdc.cam.ac.uk/structures/Search?Ccdcid=SOGVIW&DatabaseToSearch=Published)  (Wang et al. 2019)  *P*2_1_/*c* *a=*7.536Å  *b=*8.149Å  *c=*28.44Å  β=94.93° V*=*1740.31Å^3^ Z*=*4 | ***(tol)^-^(*H*pip)^+^***  [SOGVOC](https://www.ccdc.cam.ac.uk/structures/Search?Ccdcid=SOGVOC&DatabaseToSearch=Published)  (Wang et al. 2019)  *P*2_1_/*n* *a=*10.827Å  *b=*7.502Å  *c=*21.322Å  β=97.39° V*=*1717.49Å^3^ Z*=*4 | ***(mec)^-^(*H*pip)^+^***  [SAXPOY01](https://www.ccdc.cam.ac.uk/structures/Search?Ccdcid=SAXPOY01&DatabaseToSearch=Published)  (Sanphui, Bolla, and Nangia 2012)  *P*2_1_2_1_2_1_ *a=*7.929Å  *b=*8.168Å  *c=*28.381Å  V*=*1838.07Å^3^ Z*=*4 |  | ***(nif)^-^_2_(*H_2_*pip)^2+^***  [RIQXIB](https://www.ccdc.cam.ac.uk/structures/Search?Ccdcid=RIQXIB&DatabaseToSearch=Published)  (Mittapalli et al. 2019)  *R*$\bar{3}$ *a=38.266*Å  *b=38.266*Å  *c=5.999*Å  V*=7607.64*Å^3^ Z*=9* |
|  |  | ***(mef)^-^_2_(*H_2_*pip)^2+^***  [SOFYOE](https://www.ccdc.cam.ac.uk/structures/Search?Ccdcid=SOFYOE&DatabaseToSearch=Published)  (Wang et al. 2019)  *Pbca* *a=*10.186Å  *b=*7.577Å  *c=*38.191Å  V*=*2947.59Å^3^ Z*=*4 | ***(tol)^-^_2_(*H_2_*pip)^2+^***  [SOGNOU](https://www.ccdc.cam.ac.uk/structures/Search?Ccdcid=SOGNOU&DatabaseToSearch=Published)  (Wang et al. 2019)  *Pbca* *a=*10.303Å  *b=*7.617Å  *c=*38.517Å  V*=*3022.85Å^3^ Z*=*4 | ***(mec)^-^_2_(*H_2_*pip)^2+^***  [SAXQEP](https://www.ccdc.cam.ac.uk/structures/Search?Ccdcid=SAXQEP&DatabaseToSearch=Published)  (Sanphui, Bolla, and Nangia 2012)  *P*$\bar{1}$ *a=*7.910Å  *b=*11.128Å  *c=*18.118Å  α=81.94°  β=80.42°  γ=88.81° V*=*1557.34Å^3^ Z*=*4 |  |  |
|  |  | ***(mef)^-^_2_(*H_2_*pip)^2+^·4H_2_O***  [RUVNOM](https://www.ccdc.cam.ac.uk/structures/Search?Ccdcid=RUVNOM&DatabaseToSearch=Published)  (Fonari et al. 2010)  *P*$\bar{1}$ *a=*6.929Å  *b=*7.174Å  *c=*18.621Å  α=85.99°  β=83.24°  γ=65.81° V*=*838.35Å^3^ Z*=*1 |  | ***(mec)^-^(*H*pip)^+^·H_2_O***  [SAXQAL](https://www.ccdc.cam.ac.uk/structures/Search?Ccdcid=SAXQAL&DatabaseToSearch=Published)  (Sanphui, Bolla, and Nangia 2012)  *P*2_1_/*c* *a=*16.137Å  *b=*8.488Å  *c=*15.596Å  β=114.94° V*=*1937.21Å^3^ Z*=*4 |  |  |
| *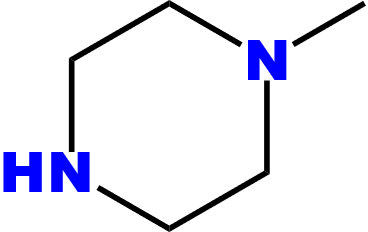*  *mep* = 4-methylpiperazine  1st p*K*a=9.33  2nd p*K*a=4.86 |  | ***(mef)^-^(*H*mep)^+^***  [BINYAB](https://www.ccdc.cam.ac.uk/structures/Search?Ccdcid=BINYAB&DatabaseToSearch=Published)  (Bouanga Boudiombo and Jacobs 2018)  *P*2_1_/c *a=*7.593Å  *b=*15.389Å  *c=* 15.791Å  β=91.96° V*=*1844.87Å^3^ Z*=*4 |  |  |  |  |
| *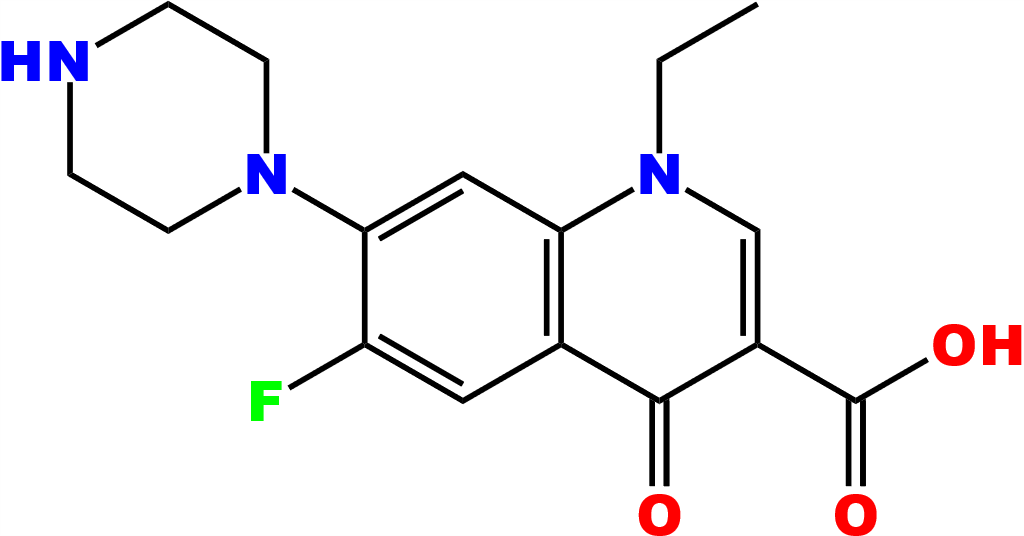*  *cefo* = 4-(3-carboxy-1-ethyl-6-fluoro-4-oxo-1,4-dihydroquinolin-7-yl) piperazine  p*K*a=8.77 |  | ***(mef)^-^(*H*cefo)^+^***  [GIMXIM](https://www.ccdc.cam.ac.uk/structures/Search?Ccdcid=GIMXIM&DatabaseToSearch=Published)  (Bhattacharya et al. 2018)  *P*$\bar{1}$ *a=*7.062Å  *b=*13.631Å  *c=*15.304Å  α=73.35°  β=77.59°  γ=80.51° V*=*1369.71Å^3^ Z*=*2 |  |  |  |  |
| *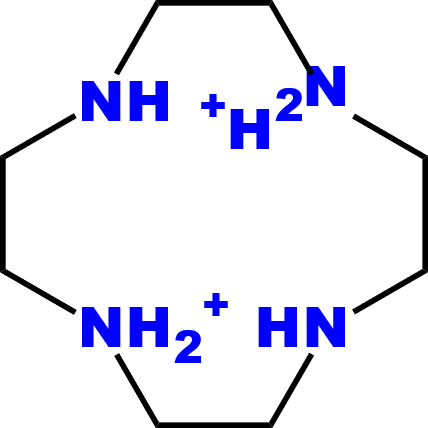*  *aacd* = 4,10-Diaza-1,7-diazoniacyclododecane  1st p*K*a=10.17  2nd p*K*a=9.27 |  | ***(mef) ^-^_2_ (aacd)^2+^·2H_2_O***  [RUVNIG](https://www.ccdc.cam.ac.uk/structures/Search?Ccdcid=RUVNIG&DatabaseToSearch=Published)  (Fonari et al. 2010)  (2:1)  *C*2/*c* *a=*32.809Å  *b=*7.659Å  *c=*29.684Å  β=104.45° V*=*7223.31Å^3^ Z*=*8 |  |  |  |  |
| *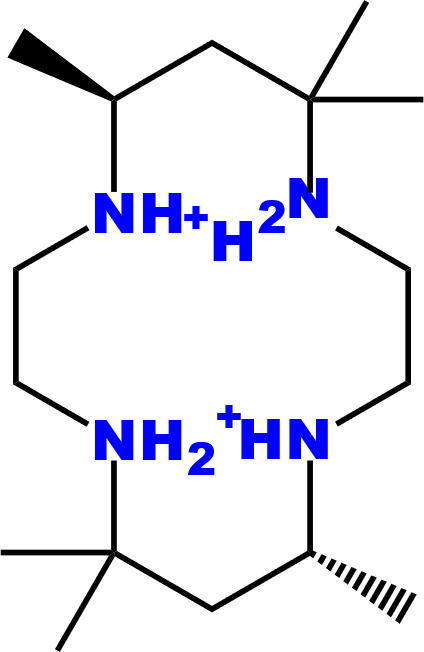*  *maacd* = (5R,12S)-5,7,7,12,14,14-Hexamethyl-4,11-diaza-1,8-diazoniacyclotetradecane  1st p*K*a=10.16  2nd p*K*a=9.27 |  | ***(mef_2_)^-^(*H_2_*maacd)^2+^·2H_2_O***  [RUVNUS](https://www.ccdc.cam.ac.uk/structures/Search?Ccdcid=RUVNUS&DatabaseToSearch=Published)  (Fonari et al. 2010)  (2:1)  *P*$\bar{1}$ *a=*8.565Å  *b=*8.718Å  *c=*16.021Å  α=102.38°  β=91.41°  γ=112.49° V*=*1071.97Å^3^ Z*=*1 |  |  |  |  |
| *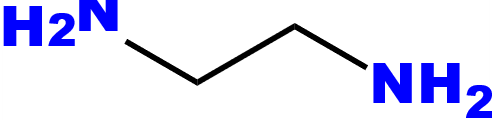*  *eta* = ethane-1,2-diamine  p*K*a= 9.55 |  | ***(mef)_2_^-^(*H_2_*eta)^2+^***  [BINXOO](https://www.ccdc.cam.ac.uk/structures/Search?Ccdcid=BINXOO&DatabaseToSearch=Published)  (Bouanga Boudiombo and Jacobs 2018)  (2:1)  *P*$\bar{1}$ *a=*6.795Å  *b=*7.108Å  *c=*16.056Å  α=93.56°  β=100.86°  γ=112.63° V*=*695.12Å^3^ Z*=*1 |  |  |  |  |
| *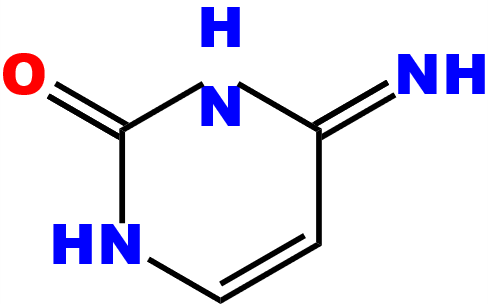*  *ohpi* = 2-Oxo-2,3-dihydropyrimidin-4-imine  p*K*a=3.16 |  | ***(mef)^-^(*H*ohpi)^+^ (ohpi)***  [ZAZGEO](https://www.ccdc.cam.ac.uk/structures/Search?Ccdcid=ZAZGEO&DatabaseToSearch=Published)  (SeethaLekshmi and Guru Row 2012)  (2:1)  *P*$\bar{1}$ *a=*6.966Å  *b=*7.321Å  *c=*23.801Å  α=86.95°  β=83.94°  γ=67.85° V*=*1117.82Å^3^ Z*=*2 |  |  |  |  |
| *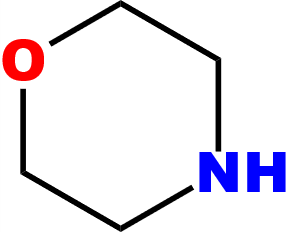*  *mor* = morpholinium  p*K*a=8.51 |  | ***(mef)^-^(*H*mor)^+^***  [BINYEF](https://www.ccdc.cam.ac.uk/structures/Search?Ccdcid=BINYEF&DatabaseToSearch=Published)  (Bouanga Boudiombo and Jacobs 2018)  *P*$\bar{1}$ *a=*7.825Å  *b=*9.889Å  *c=*12.291Å  α=79.1°  β=73.65°  γ=74.14° V*=*871.38Å^3^ Z*=*2 |  |  |  |  |
| *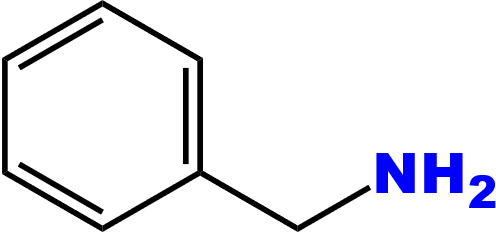*  *phm* = phenylmethanamine  p*K*a=*9.30* |  |  |  |  |  | ***(nif)^-^(Hphm*)^+^**  [RIQXUN](https://www.ccdc.cam.ac.uk/structures/Search?Ccdcid=RIQXUN&DatabaseToSearch=Published)  [RIQXUN01](https://www.ccdc.cam.ac.uk/structures/Search?Ccdcid=RIQXUN&DatabaseToSearch=Published)  (Mittapalli et al. 2019)  *P*$\bar{1}$ *a=4.731*Å  *b=10.913*Å  *c=18.451*Å  α=75.255°  β=85.065°  γ=82.971° V*=912.75*Å^3^ Z*=2* |
| *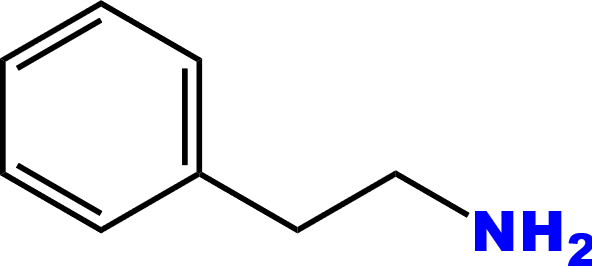*  *phe* = phenylethanamine p*K*a=9.79 |  |  | ***(tol)^-^(*H*phe)^+^***  [YAWLAM](https://www.ccdc.cam.ac.uk/structures/Search?Ccdcid=YAWLAM&DatabaseToSearch=Published)  (Parveen, Sravanthi, and Dastidar 2017)  *Pcc*2 *a=*15.389Å  *b=*17.224Å  *c=*7.555Å  V*=*2002.17Å^3^ Z*=*4 |  |  |  |
| *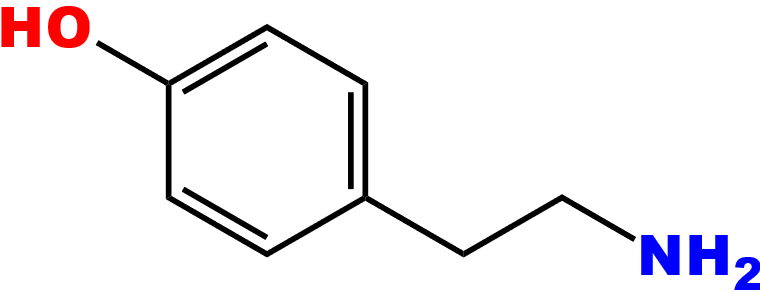*  *het* = 2-(4-hydroxyphenyl)ethylamine  p*K*a=9.66 |  |  | ***(tol)^-^(*H*het)^+^***  [YAWLEQ](https://www.ccdc.cam.ac.uk/structures/Search?Ccdcid=YAWLEQ&DatabaseToSearch=Published)  (Parveen, Sravanthi, and Dastidar 2017)  *I*2/*a* *a=*18.332Å  *b=*7.701Å  *c=*28.664Å  β=102.35° V*=*3957.59Å^3^ Z*=*8 |  |  | ***(nif)^-^(*H*het)^+^***  [RIQXOH](https://www.ccdc.cam.ac.uk/structures/Search?Ccdcid=RIQXOH&DatabaseToSearch=Published)  (Mittapalli et al. 2019)  *P*2_1_/*c* *a=13.806*Å  *b=8.090*Å  *c=17.873*Å  β=93.66°  V*=1992.17*Å^3^ Z*=4* |
| *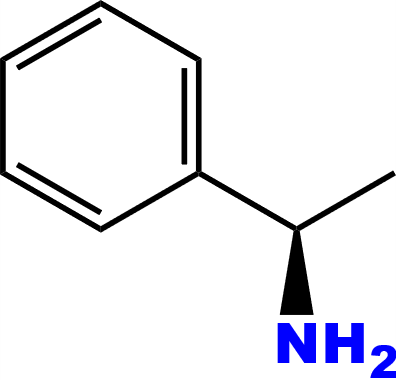*  *pea* = (R)-1-Phenylethylammine  p*K*a=*9.52* |  |  |  |  |  | ***(nif)^-^(H*pea)^+^**  [VAKVOU](https://www.ccdc.cam.ac.uk/structures/Search?Ccdcid=VAKVOU&DatabaseToSearch=Published)  (Lemmerer et al. 2010)  *P*2_1_  *a=12.416*Å  *b=5.554*Å  *c=28.162*Å  β=94.21° V*=1936.57*Å^3^ Z*=2* |
| *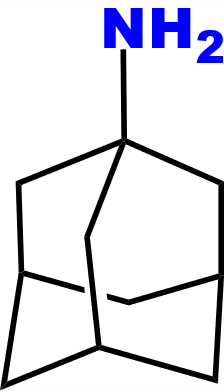*  *ada* = adamantan-1-amine  p*K*a=10.71 |  | ***(mef)^-^(*H*ada)^+^***  [PUHVAR](https://www.ccdc.cam.ac.uk/structures/Search?Ccdcid=PUHVAR&DatabaseToSearch=Published)  (Roy et al. 2014)  *P*$\bar{1}$ *a=*13.007Å  *b=*14.017Å  *c=*14.409Å  α=62.98°  β=80.33°  γ=66.67° V*=*2149.26Å^3^ Z*=*4 | ***(tol)^-^(*H*ada)^+^·sol***  [PUHTAP](https://www.ccdc.cam.ac.uk/structures/Search?Ccdcid=PUHTAP&DatabaseToSearch=Published)  (Roy et al. 2014)  *P*2_1_/*c* *a=*6.629Å  *b=*19.332Å  *c=*18.807Å  β=95.45° V*=*2399.40Å^3^ Z*=*4 | ***(mec)^-^(*H*ada)^+^***  [PUHTUJ](https://www.ccdc.cam.ac.uk/structures/Search?Ccdcid=PUHTUJ&DatabaseToSearch=Published)  (Roy et al. 2014)  *P*$\bar{1}$ *a=*12.545Å  *b=*13.937Å  *c=*15.612Å  α=111.47°  β=111.53°  γ=94.37° V*=*2291.91Å^3^ Z*=*4 | ***(flu)^-^(*H*ada)^+^***  [SOZGAR](https://www.ccdc.cam.ac.uk/structures/Search?Ccdcid=SOZGAR&DatabaseToSearch=Published)  (Roy et al. 2014)  *P*2_1_/*c* *a=*11.337Å  *b=*6.526Å  *c=*29.981Å  β=98.849° V*=*2191.972Å^3^ Z*=*4 |  |
| *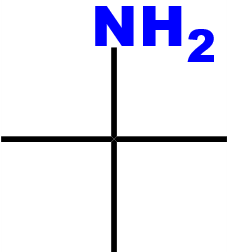*  *mpa* = 2-methylpropan-2-amine  p*K*a=10.65 |  | ***(mef)^-^(*H*mpa)^+^***  [TAMCIW01](https://www.ccdc.cam.ac.uk/structures/Search?Ccdcid=TAMCIW01&DatabaseToSearch=Published)  (Nechipadappu and Trivedi 2017; Ranjan et al. 2020)  *P*$\bar{1}$ *a=*7.8149Å  *b=*8.1357Å  *c=*16.3674Å  α=79.58°  β=81.33°  γ=89.95° V*=*1011.43Å^3^ Z*=*2 |  | ***(mec)^-^(*H*mpa)^+^***  [TEQCOK](https://www.ccdc.cam.ac.uk/structures/Search?Ccdcid=TEQCOK&DatabaseToSearch=Published)  (Roy and Dastidar 2017)  *C*2/*c* *a=*27.003Å  *b=*10.628Å  *c=*28.498Å  β=106.73° V*=*7831.86Å^3^ Z*=*16 | ***(flu)^-^(*H*mpa)^+^***  [TEQCUQ](https://www.ccdc.cam.ac.uk/structures/Search?Ccdcid=TEQCUQ&DatabaseToSearch=Published)  (Roy and Dastidar 2017)  *P*2_1_/c *a=*14.752Å  *b=*6.492Å  *c=*19.455Å  β=103.58° V*=*1811.08Å^3^ Z*=*4 |  |
| *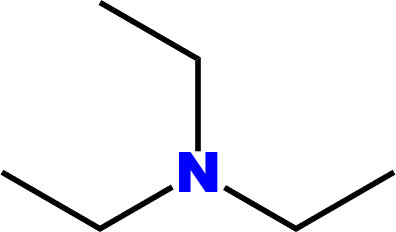*  *tea* = triethylamine |  | ***(mef)^-^(*H*tea)^+^***  [BINXUU](https://www.ccdc.cam.ac.uk/structures/Search?Ccdcid=BINXUU&DatabaseToSearch=Published)  (Bouanga Boudiombo and Jacobs 2018)  *Pbca* *a=*11.201Å  *b=*15.206Å  *c=*22.491Å  V*=*3830.72Å^3^ Z*=*8 |  |  |  |  |
| *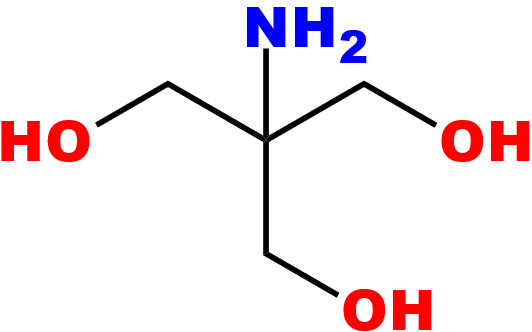*  *dhpa* = 1,3-dihydroxy-2-(hydroxymethyl) propan-2-amine  p*K*a=8.95 |  | ***(mef)^-^(*H*dhpa)^+^·H_2_O***  [RUVNEC01](https://www.ccdc.cam.ac.uk/structures/Search?Ccdcid=RUVNEC01&DatabaseToSearch=Published)  (Roy and Dastidar 2017)  *P*$\bar{1}$ *a=*7.796Å  *b=*8.461Å  *c=*15.224Å  α=90.29°  β=98.03°  γ=97.29° V*=*985.91Å^3^ Z*=*2 | ***(tol)^-^(*H*dhpa)^+^***  [TERLIO](https://www.ccdc.cam.ac.uk/structures/Search?Ccdcid=TERLIO&DatabaseToSearch=Published)  (Roy and Dastidar 2017)  *P*$\bar{1}$ *a=*10.811Å  *b=*12.625Å  *c=*14.418Å  α=92.07°  β=91.31°  γ=109.01° V*=*1858.068Å^3^ Z*=*4 | ***(mec)^-^(*H*dhpa)^+^·CH_3_OH***  [TERLEK](https://www.ccdc.cam.ac.uk/structures/Search?Ccdcid=TERLEK&DatabaseToSearch=Published)  (Roy and Dastidar 2017)  *Cc* *a=*10.828Å  *b=*12.782Å  *c=*32.954Å  β=99.26° V*=*4501.52Å^3^ Z*=*8 | ***(flu)^-^(*Hd*hpa)^+^·H_2_O***  [TEQYEW](https://www.ccdc.cam.ac.uk/structures/Search?Ccdcid=TEQYEW&DatabaseToSearch=Published)  (Roy and Dastidar 2017)  *P*$\bar{1}$ *a=*9.063Å  *b=*10.821Å  *c=*20.661Å  α=97.36°  β=96.44°  γ=92.68° V*=*1992.98Å^3^ Z*=*4 |  |
| *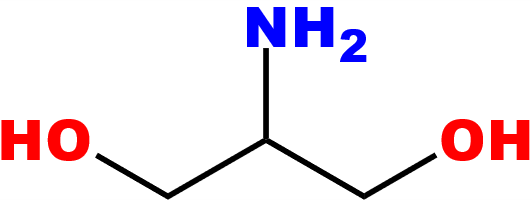*  *hpa* = 1,3-dihydroxypropan-2-amine  p*K*a=9.23 |  |  | ***(tol)^-^(*H*hpa)^+^***  [YAWLIU](https://www.ccdc.cam.ac.uk/structures/Search?Ccdcid=YAWLIU&DatabaseToSearch=Published)  (Parveen, Sravanthi, and Dastidar 2017)  *P*2_1_/*c* *a=*17.927Å  *b=*7.772Å  *c=*12.562Å  β=104.181° V*=*1696.47Å^3^  Z*=*4 |  |  |  |
| *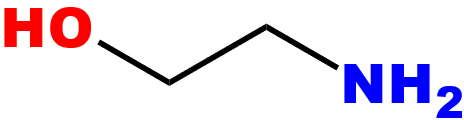*  *eth = ethanolamine*  p*K*a=9.55 |  |  |  | ***(mec)^-^(*H*eth)^+^***  [FOPMIG](https://www.ccdc.cam.ac.uk/structures/Search?Ccdcid=FOPMIG&DatabaseToSearch=Published)  (Dhanaraj and Vijayan 1987)  *P*$\bar{1}$ *a=*9.232Å  *b=*12.287Å  *c=*17.033Å  α=70.21°  β=76.72°  γ=68.21° V*=*1676.09Å^3^ Z*=*4 |  | ***(nif)^-^(*H*het)^+^· H_2_O***  [CAGXEN](https://www.ccdc.cam.ac.uk/structures/Search?Ccdcid=CAGXEN&DatabaseToSearch=Published)  (V. Dhanaraj and Vijayan 1983)  *P*$\bar{1}$ *a=6.239*Å  *b=11.280*Å  *c=12.451*Å  α=101.21°  β=92.32°  γ=99.93° V*=844.31*Å^3^ Z*=2* |
| *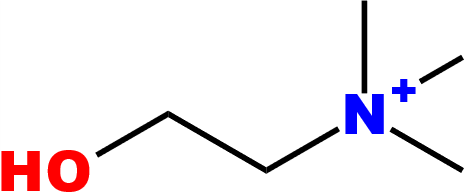*  *cho* ***=* c**holine |  |  |  | ***(mec)^-^(cho)^+^·H_2_O***  [FOPMOM](https://www.ccdc.cam.ac.uk/structures/Search?Ccdcid=FOPMOM&DatabaseToSearch=Published)  (Dhanaraj and Vijayan 1987)  *Pna*2_1_ *a=*9.637Å  *b=*12.962Å  *c=*33.099Å  V*=*4134.55Å^3^ Z*=*8 |  |  |
| *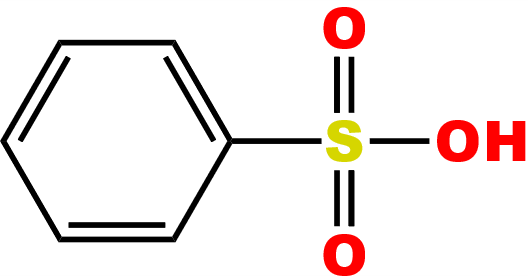*  *bsa* = benzenesulfonic acid  p*K*a=*-2.36* |  |  |  |  |  | ***(H_2_nif)^+^(bsa*)^‑^**  [RIQWUM](https://www.ccdc.cam.ac.uk/structures/Search?Ccdcid=RIQWUM&DatabaseToSearch=Published)  (Mittapalli et al. 2019)  *P*$\bar{1}$  *a=7.889*Å  *b=8.074*Å  *c=16.868*Å  α=84.05°  β=87.49°  γ=61.69° V*=940.79*Å^3^ Z*=2* |
| *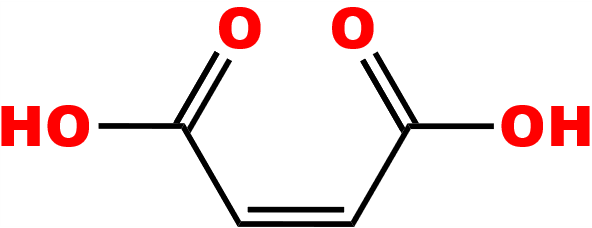*  H*mal* = maleic acid  p*K*a=*2.85* |  |  |  |  |  | ***(H_2_nif)^+^(mal*)^‑^**  [KIGMIX](https://www.ccdc.cam.ac.uk/structures/Search?Ccdcid=KIGMIX&DatabaseToSearch=Published)  (Childs, Stahly, and Park 2007)  *P*2_1_/*c*  *a=8.143*Å  *b=20.756*Å  *c=10.651*Å  β=100.276° V*=1688.73*Å^3^ Z*=4* |


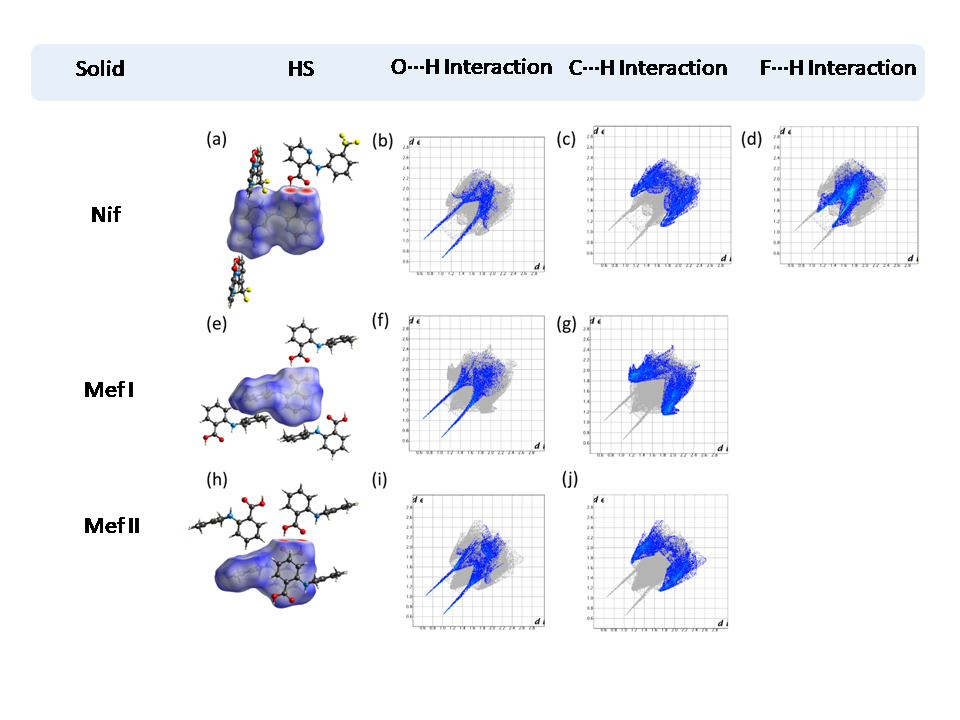


**Fig. S1.** (a, e and h) Hirshfeld surface analysis and structural environment of Nif, Mef I, and Mef II. (b-d) O∙∙∙H/H∙∙∙O, C∙∙∙H/H∙∙∙C, and F∙∙∙H/H∙∙∙F interactions resolved fingerprint plots of Nif. (f-g, and i-j) O∙∙∙H/H∙∙∙O and C∙∙∙H/H∙∙∙C interactions resolved fingerprint plots of both forms of Mef. The two spikes present in the solids are characteristic of the carboxylic acid dimer.


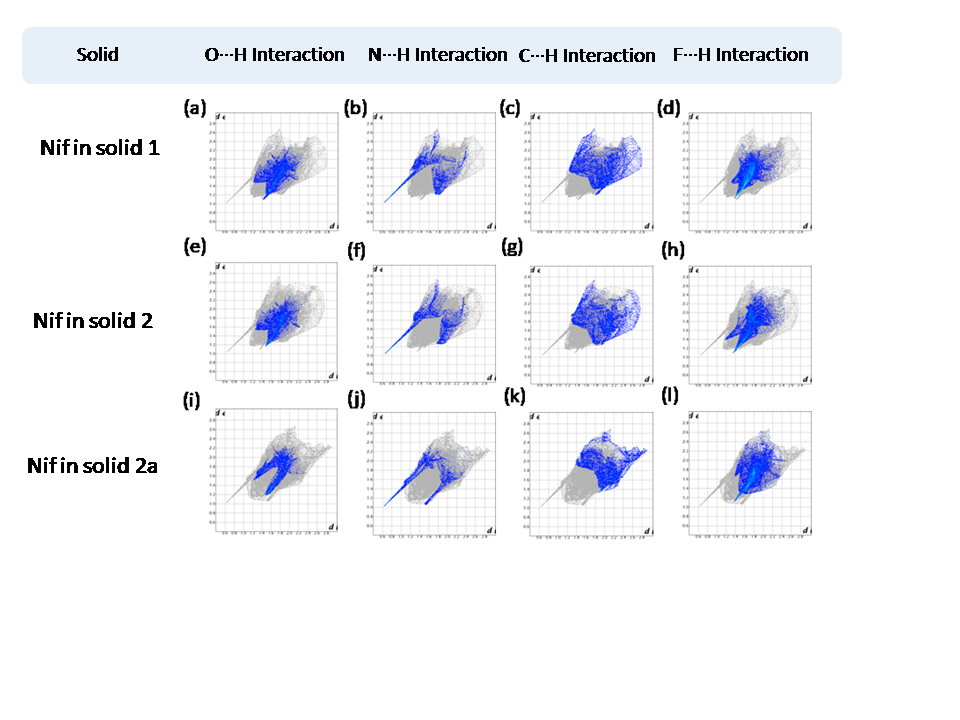


**Fig. S2.** (a-d) O∙∙∙H/H∙∙∙O N∙∙∙H/H∙∙∙N, C∙∙∙H/H∙∙∙C, and F∙∙∙H/H∙∙∙F interactions Resolved fingerprint plots of Nif in solid **1**, respectively. (e-l) O∙∙∙H/ H∙∙∙O and N∙∙∙H/H∙∙∙N interactions resolved fingerprint plots of Nif of solid **2** and **2a**, respectively.


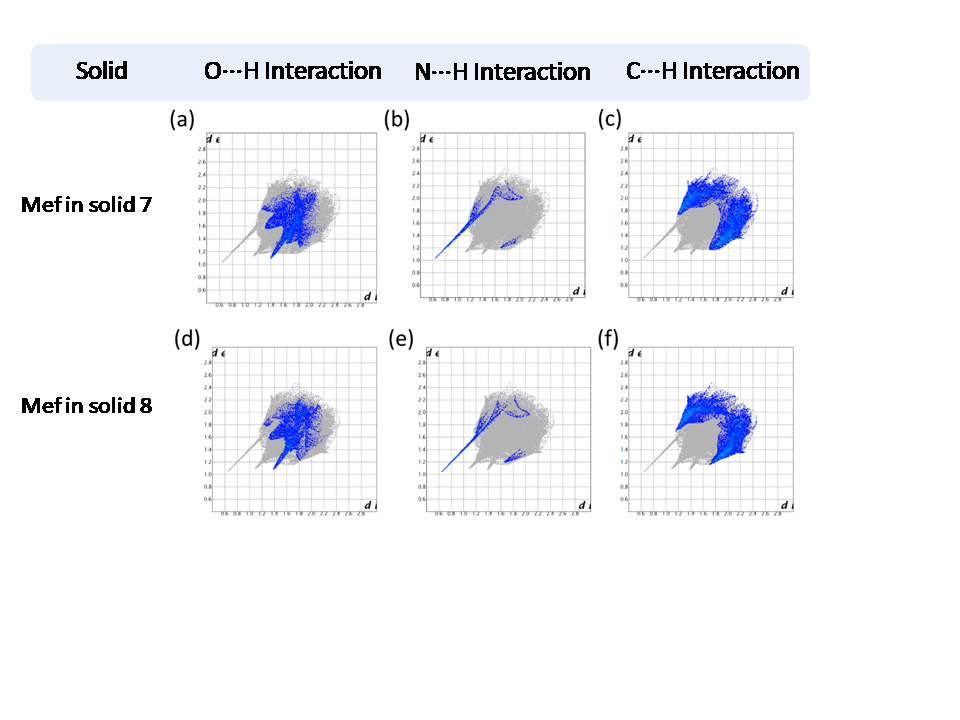


**Fig.S3.** (a-f) Resolved fingerprint plots of Mef of **7** and **8** in O∙∙∙H/ H∙∙∙O, N∙∙∙H/H∙∙∙N, and C∙∙∙H/ H∙∙∙C interactions, respectively.


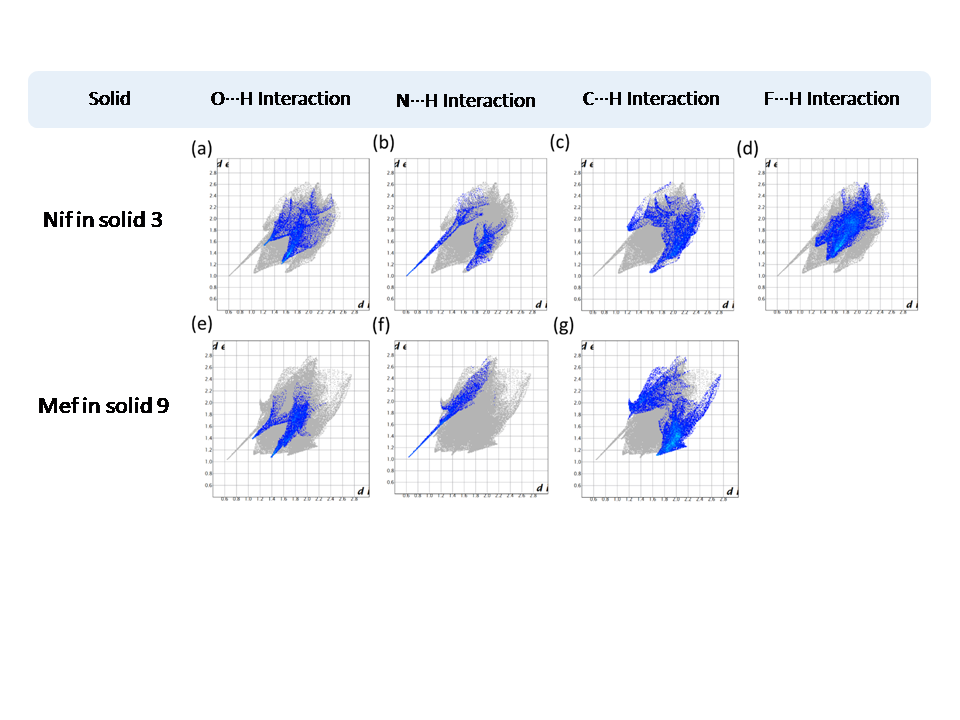


**Fig.S4.** (a-d) Resolved fingerprint plots of Nif of solid **3** in O∙∙∙H/ H∙∙∙O, N∙∙∙H/H∙∙∙N, C∙∙∙H/ H∙∙∙C, and F∙∙∙H/ H∙∙∙F interactions, respectively. (e-g) Resolved fingerprint plots of Mef of solid **9** in O∙∙∙H/ H∙∙∙O, N∙∙∙H/H∙∙∙N, and C∙∙∙H/H∙∙∙C interactions, respectively.


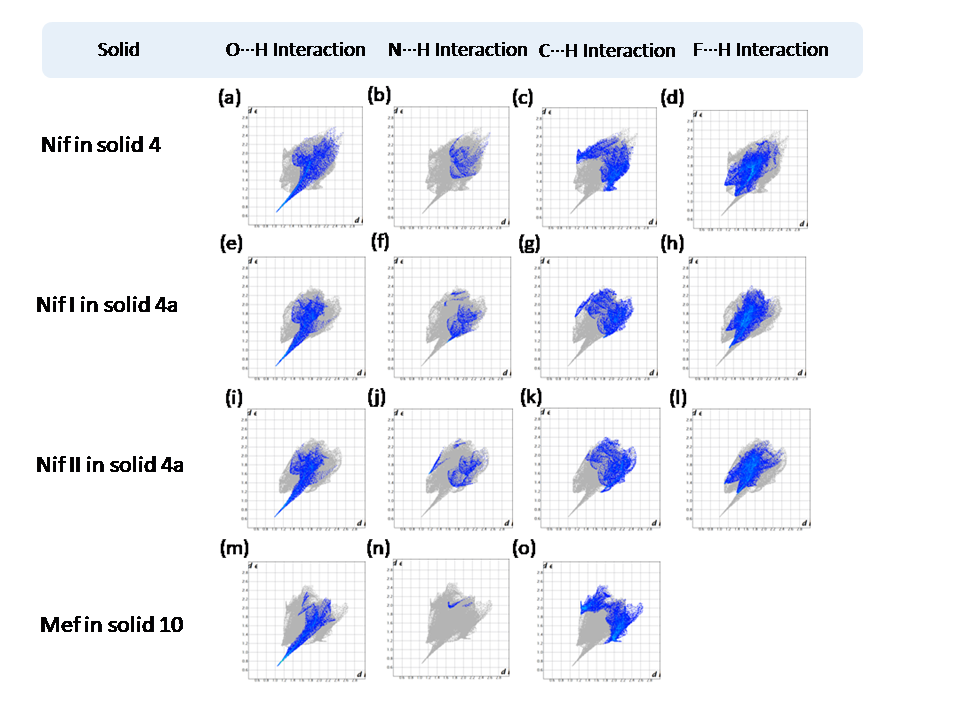


**Fig.S5.** (a-l) Resolved fingerprint plots of Nif in **4** and Nif I &Nif II in **4a** in O∙∙∙H/ H∙∙∙O, N∙∙∙H/H∙∙∙N, C∙∙∙H/H∙∙∙C, and F∙∙∙H/H∙∙∙F interactions, respectively. (m-o) Resolved fingerprint plotsofMefof**10** in O∙∙∙H/H∙∙∙O, N∙∙∙H/H∙∙∙N, and C∙∙∙H/H∙∙∙C interactions, respectively.


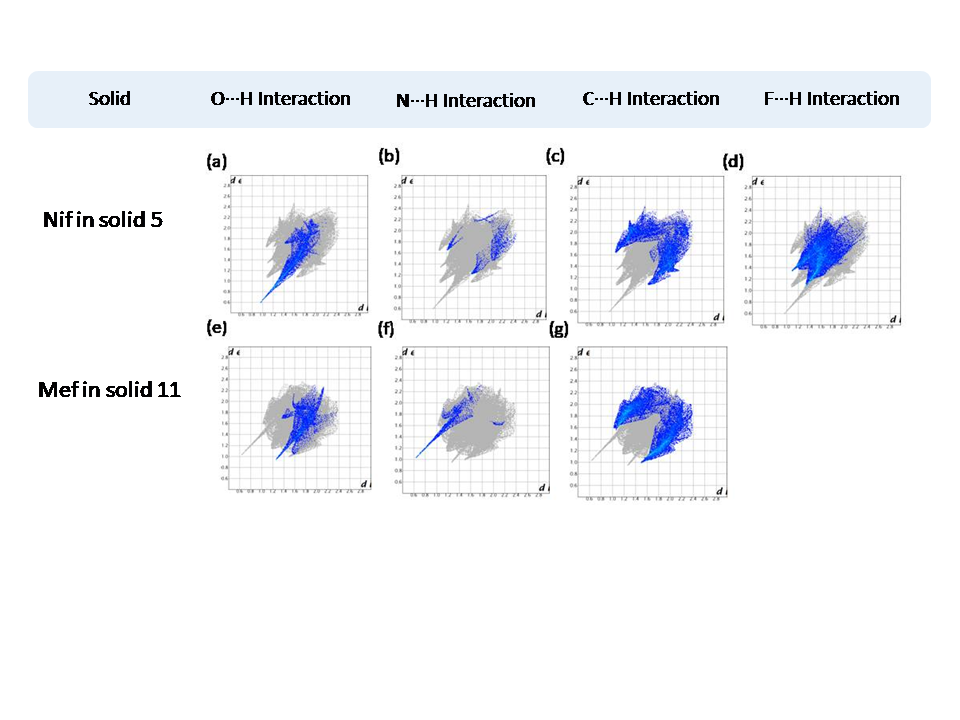


**Fig. S6.** (a-d) Resolved fingerprint plots of Nif in **5** in O∙∙∙H/H∙∙∙O, N∙∙∙H/H∙∙∙N, C∙∙∙H/H∙∙∙C, and F∙∙∙H/H∙∙∙F interactions, respectively. (e-g) Resolved fingerprint plots of Mef in **11** in O∙∙∙H/ H∙∙∙O, N∙∙∙H/H∙∙∙N, and C∙∙∙H/ H∙∙∙C interactions, respectively.


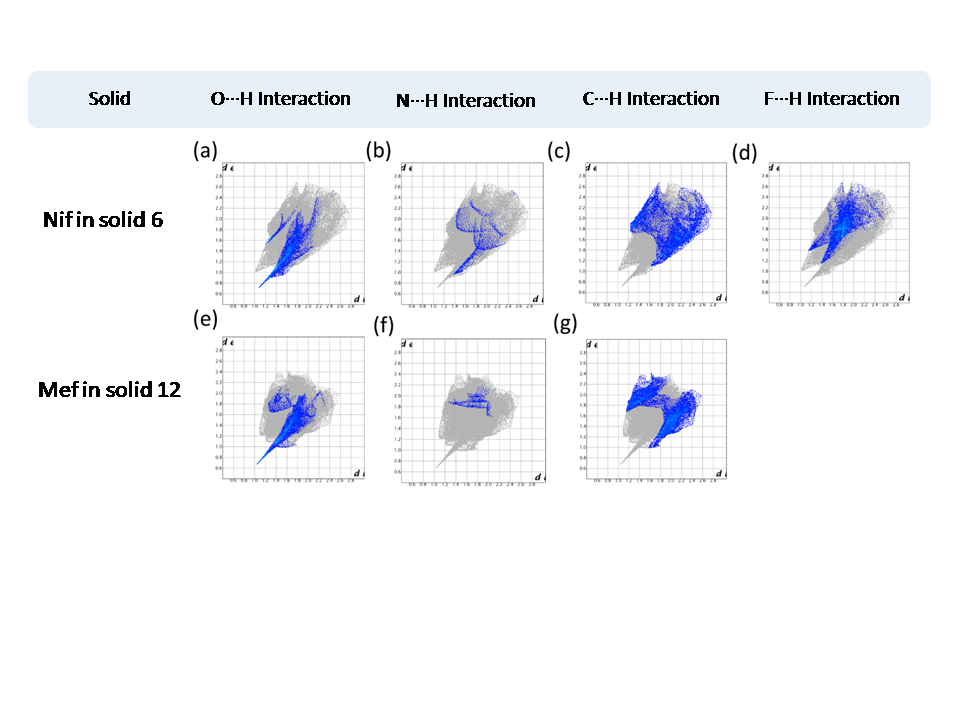


**Fig. S7.** (a-d) Resolved fingerprint plots of Nif in **6** in O∙∙∙H/H∙∙∙O, N∙∙∙H/H∙∙∙N, C∙∙∙H/H∙∙∙C, and F∙∙∙H/H∙∙∙F interactions, respectively. (e-g) Resolved fingerprint plots of Mef in **12** in O∙∙∙H/ H∙∙∙O, N∙∙∙H/H∙∙∙N, and C∙∙∙H/ H∙∙∙C interactions, respectively.


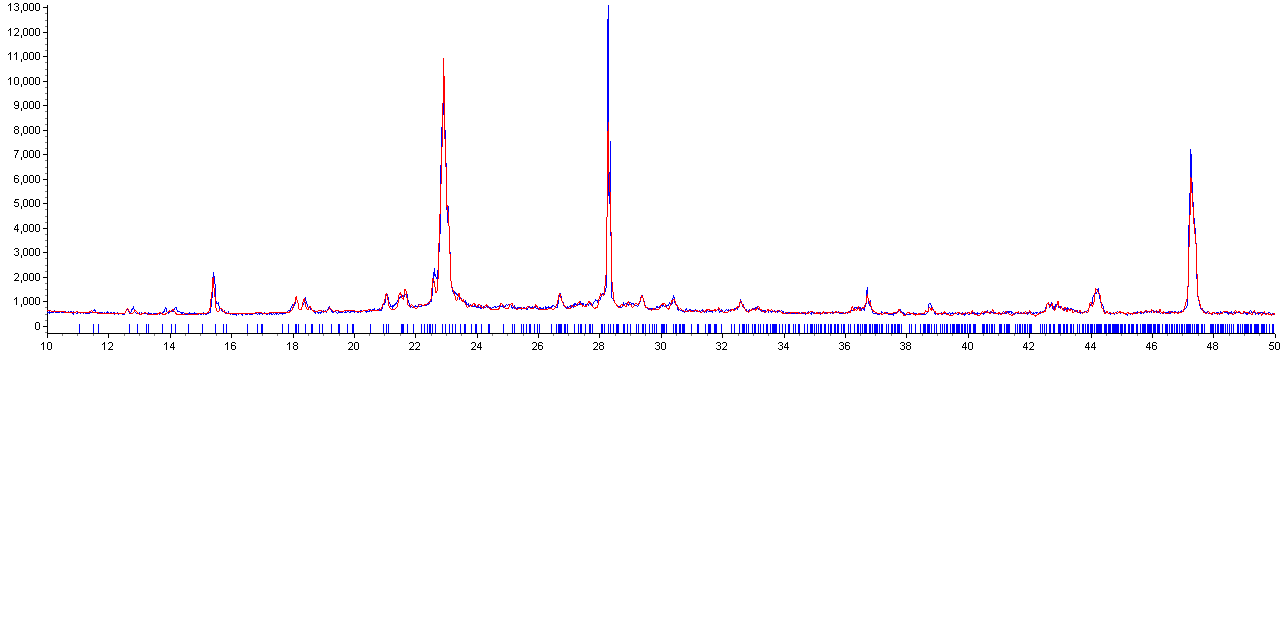


Solid 1


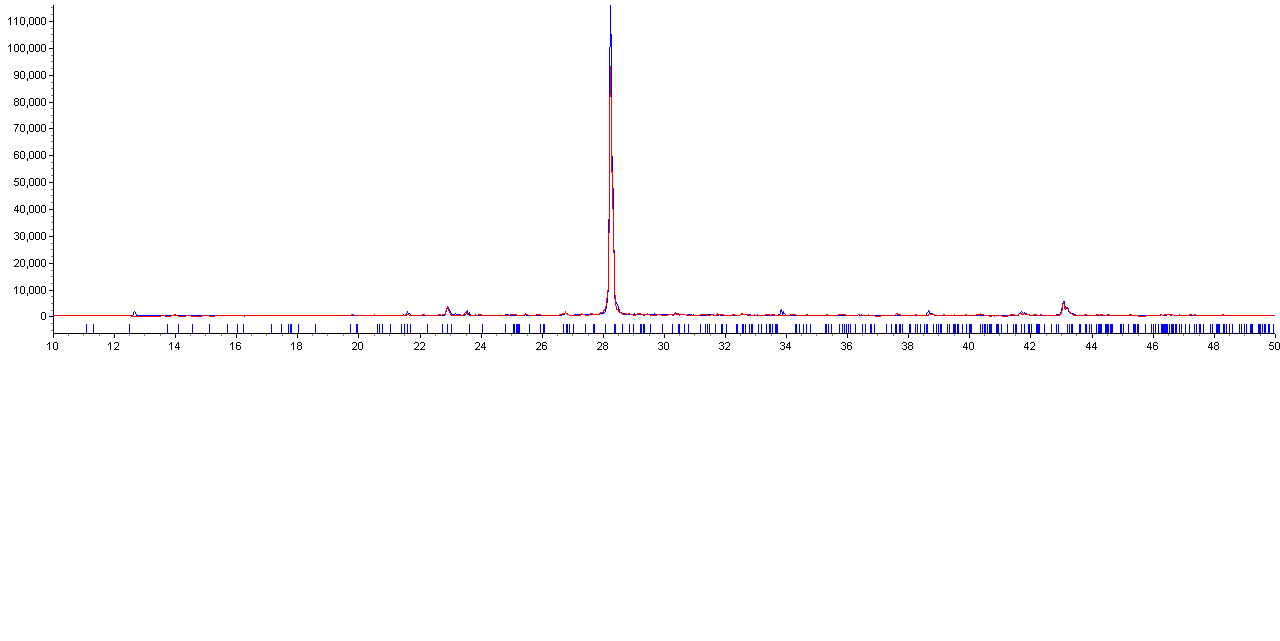


Solid 2


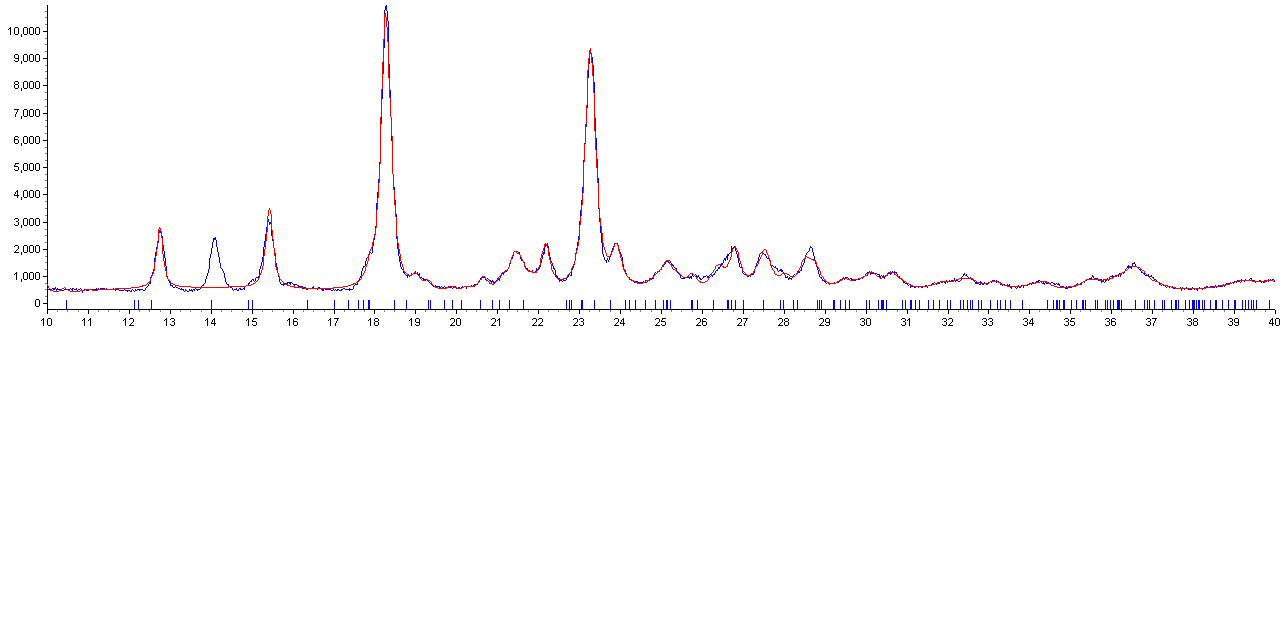


Solid 2a


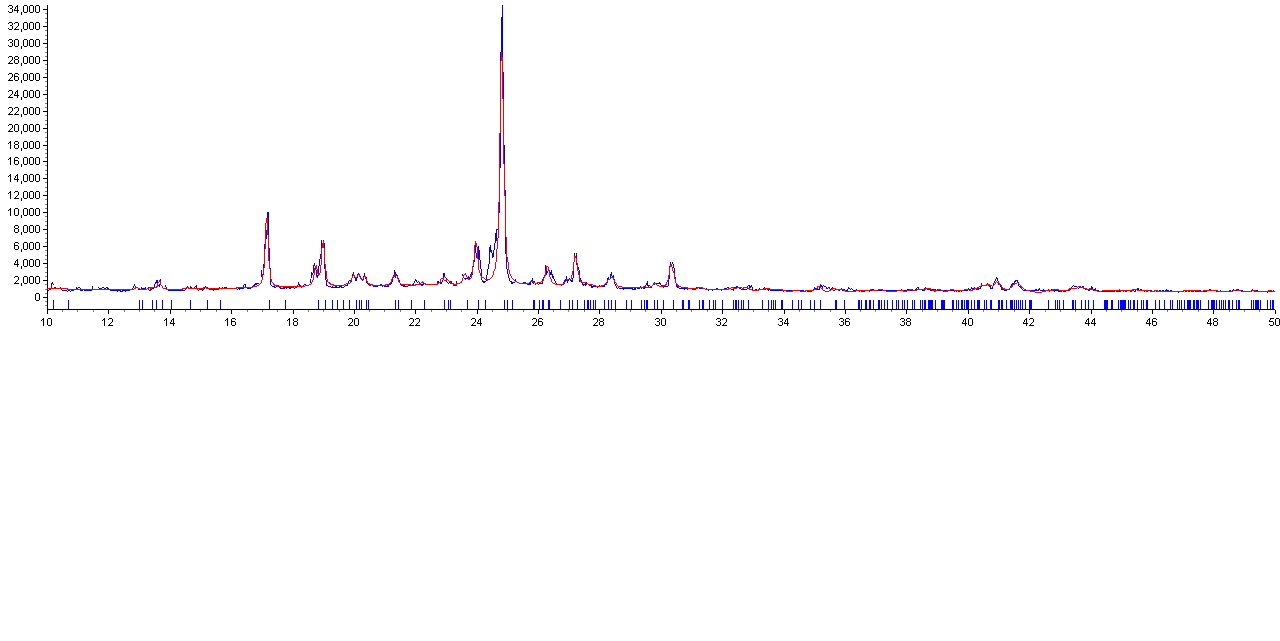


Solid 3


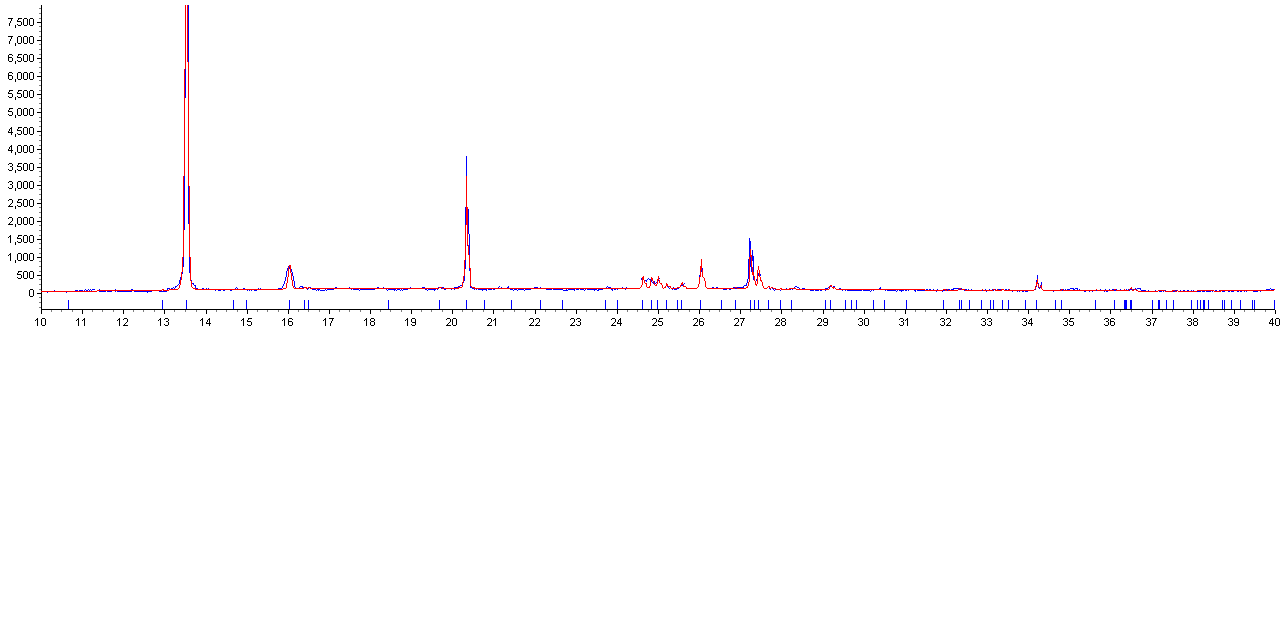


Solid 4


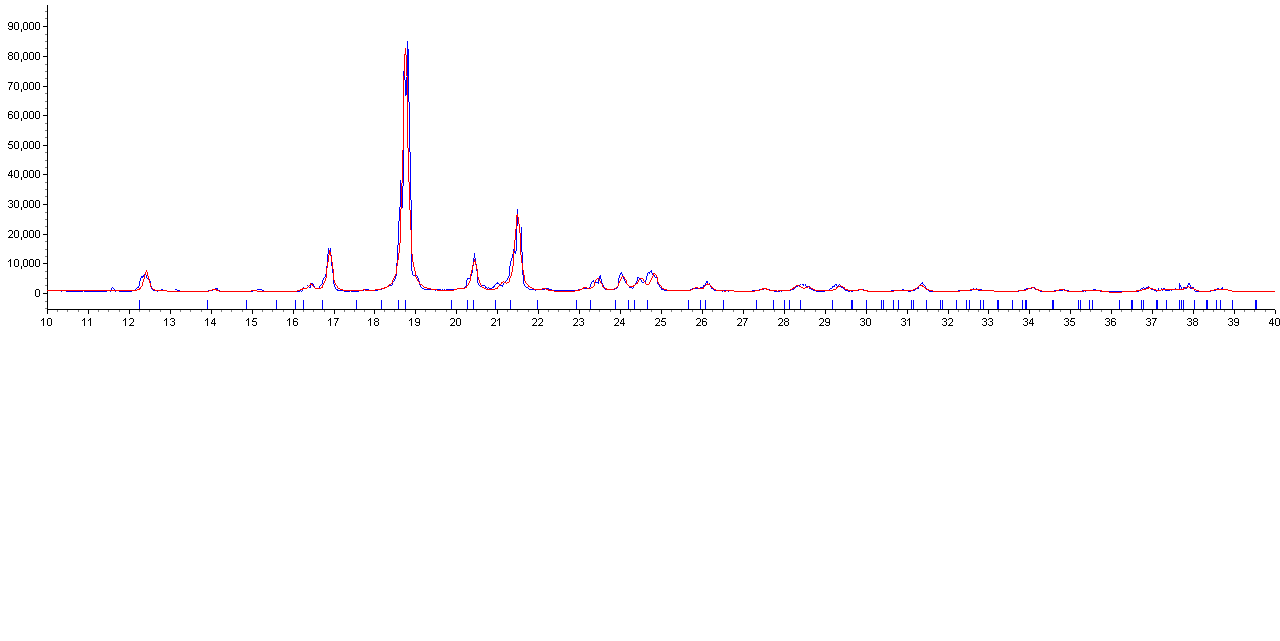


Solid 4a


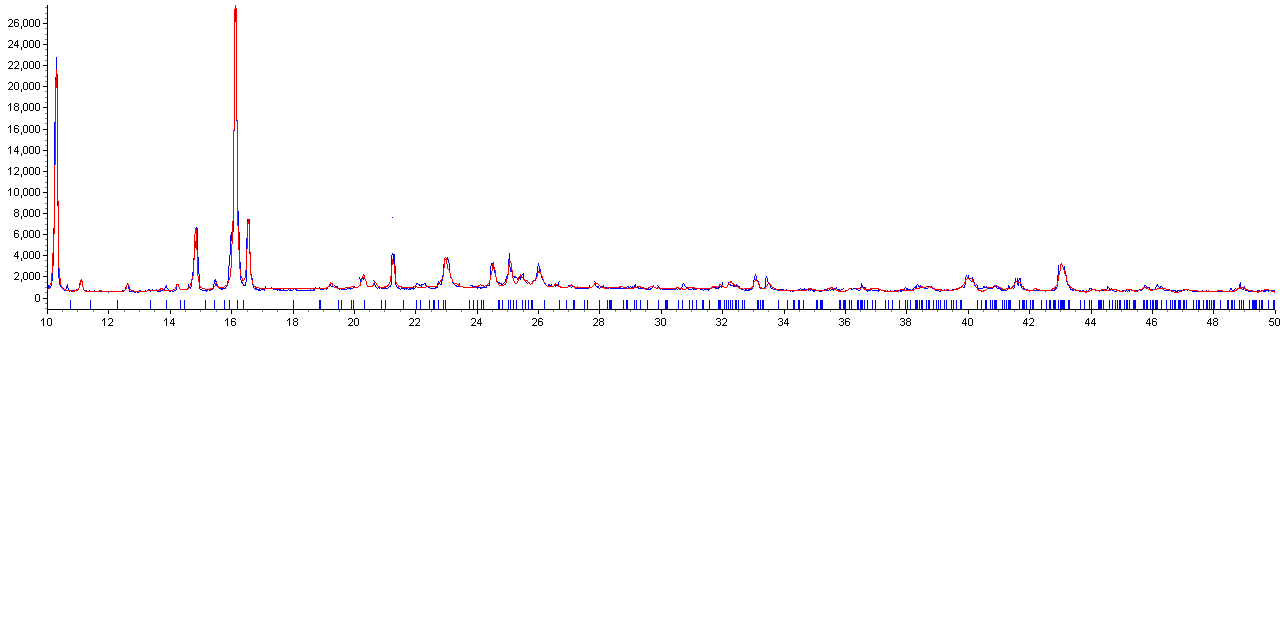


Solid 7


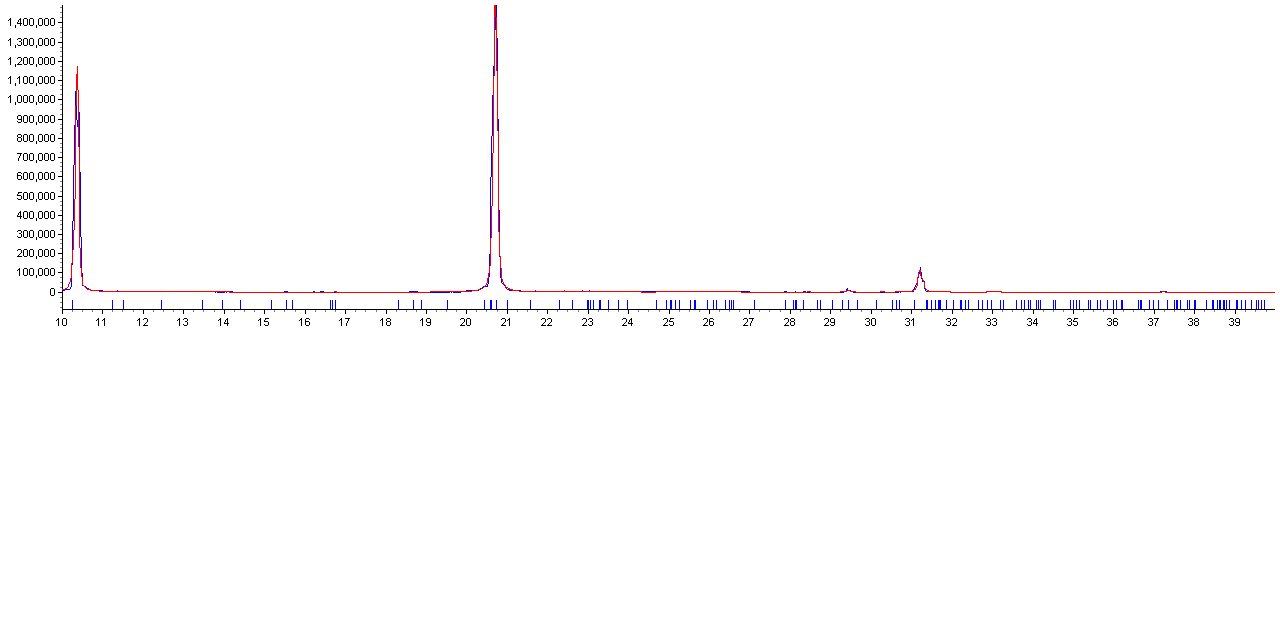


Solid 8


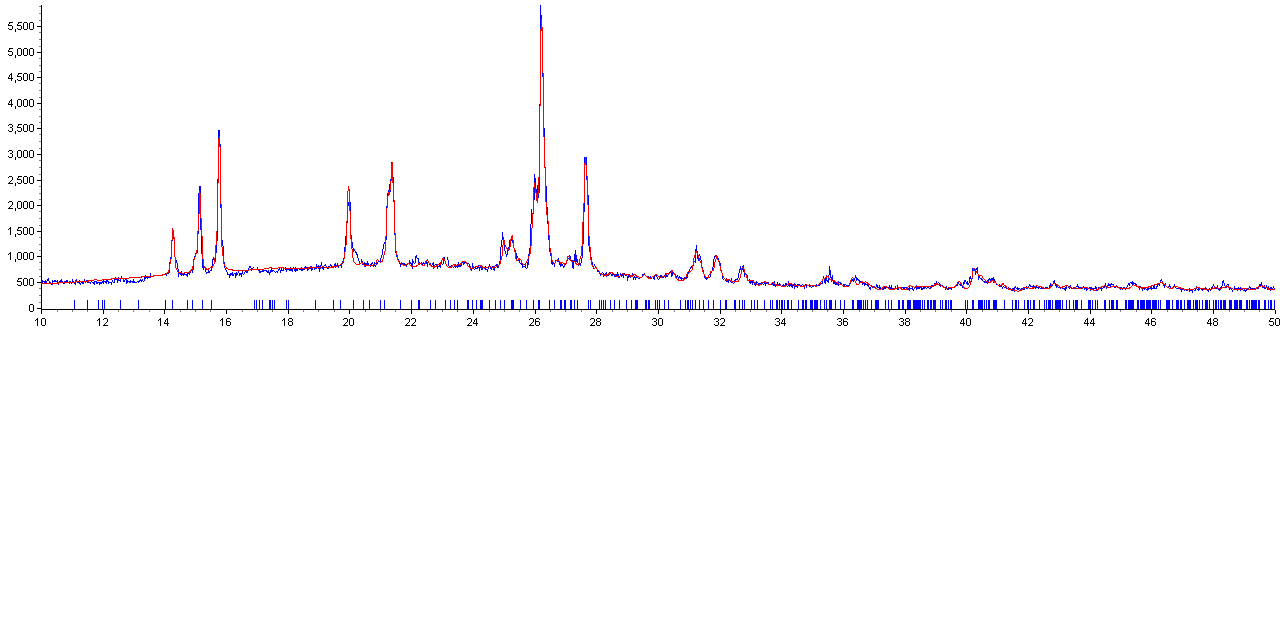


Solid 9


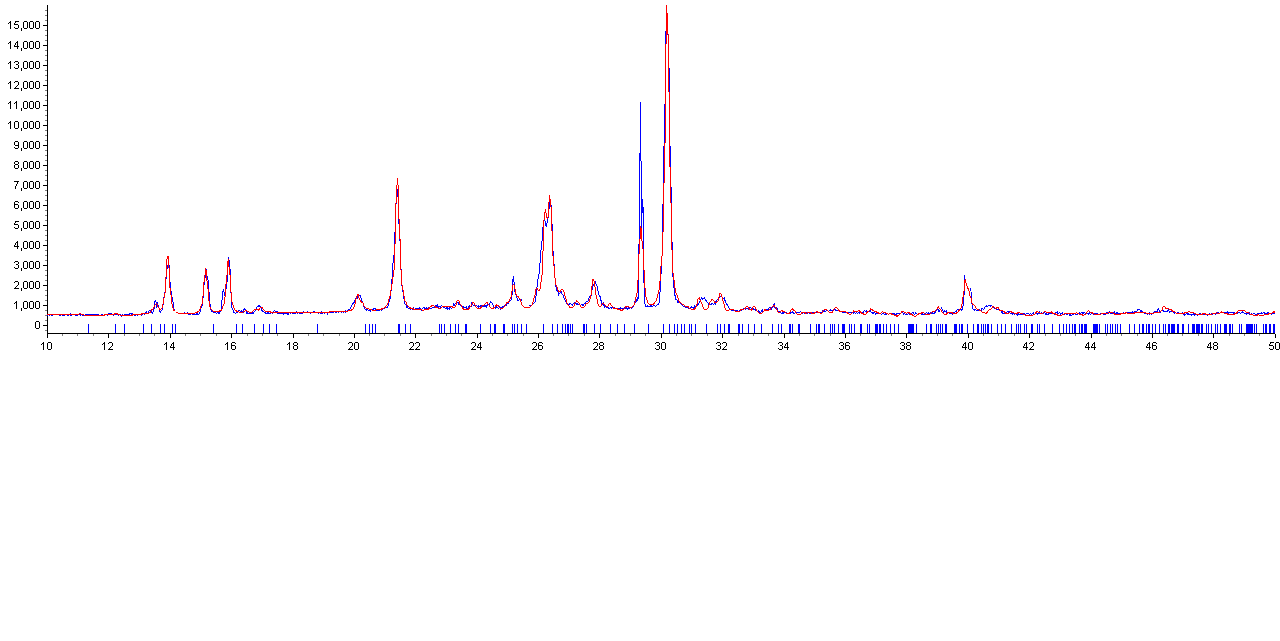


Solid 10


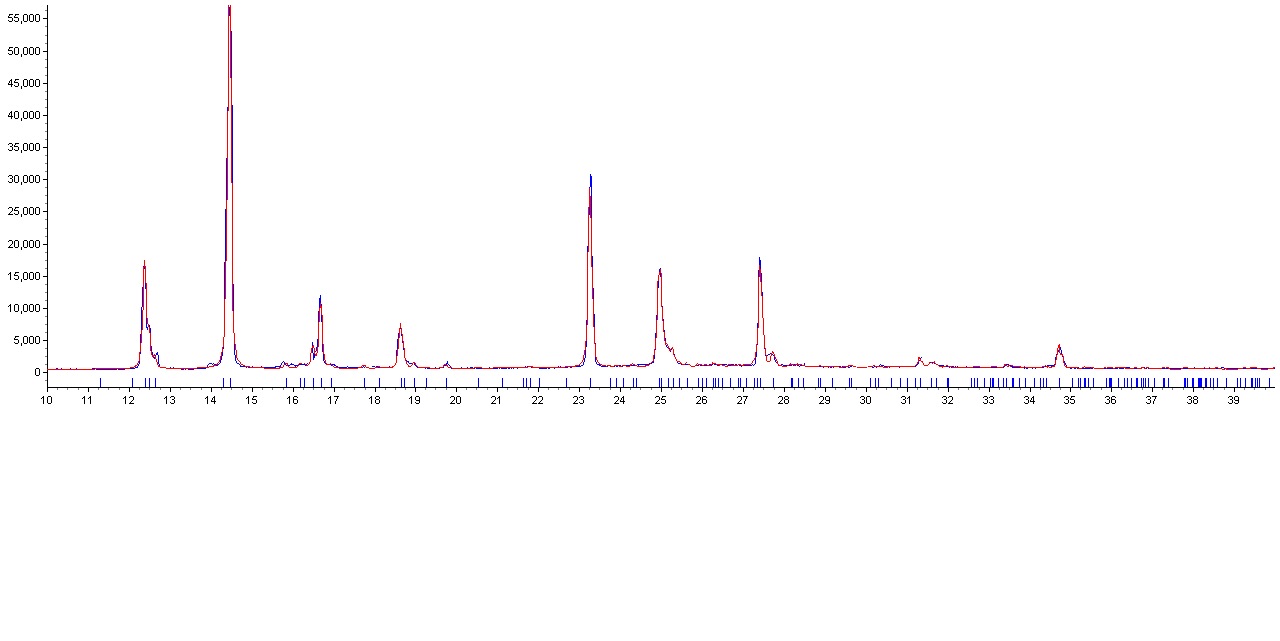


Solid 12

**Fig.S8.**  Rietveld refinement plots for **1‒12**, indicating the homogeneity of the bulk samples. The plot shows the experimental powder XRD profile (blue line), the calculated (red line), and blue tick lines show Bragg positions at the bottom.

Footnote: *Peak(s) marked with star corresponds to an unidentified phase.


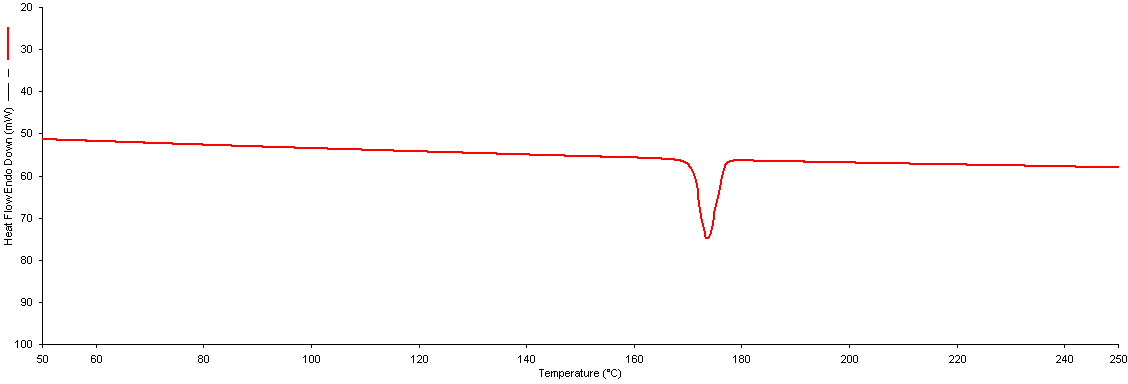


Solid 1

Peak = 177 °C


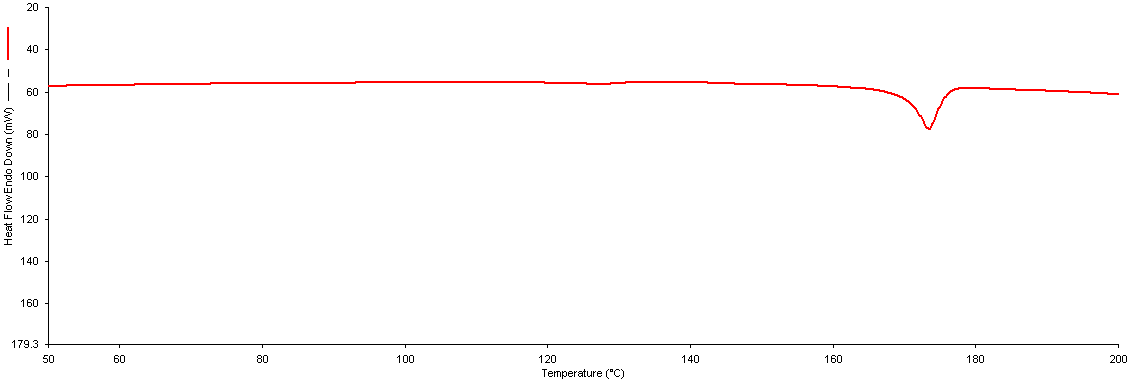


Solid 2

Peak = 179 °C


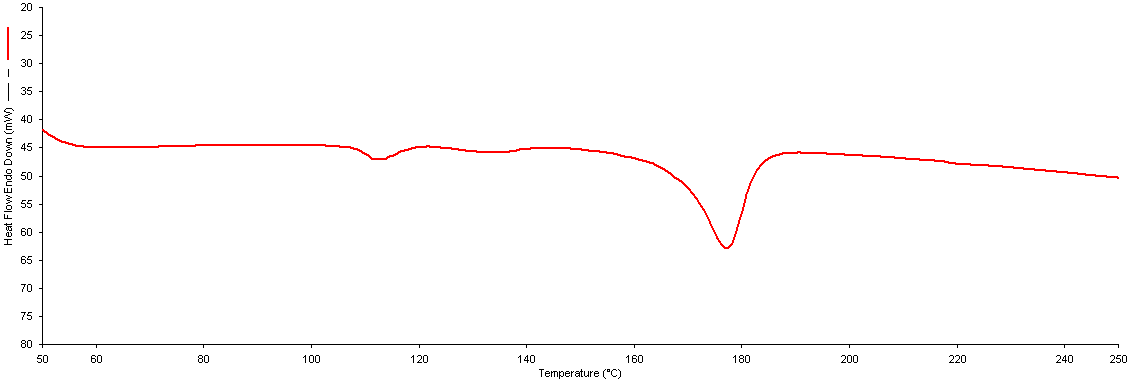


Solid 2a

Peak = 117 °C

Peak = 178 °C


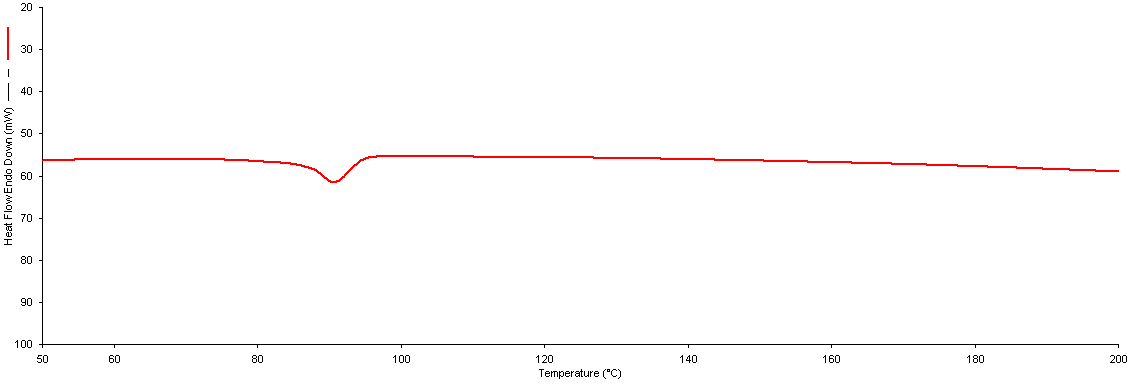


Solid 3

Peak = 92 °C


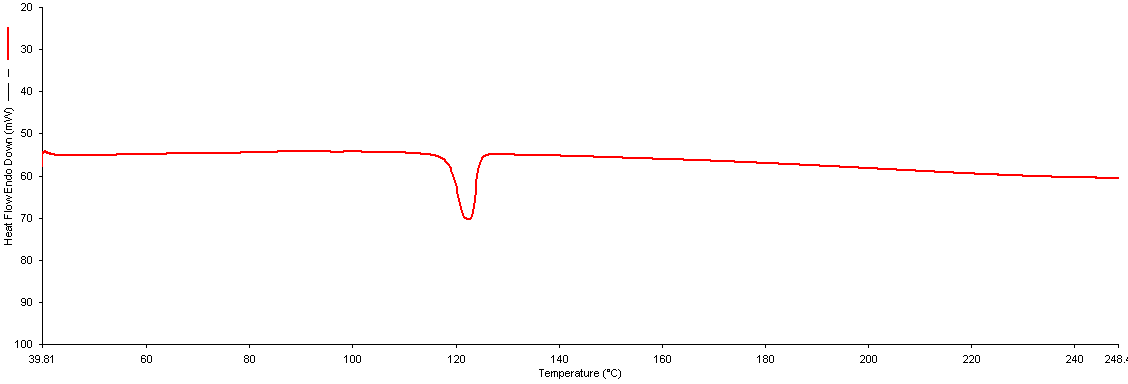


Solid 4

Peak = 123 °C


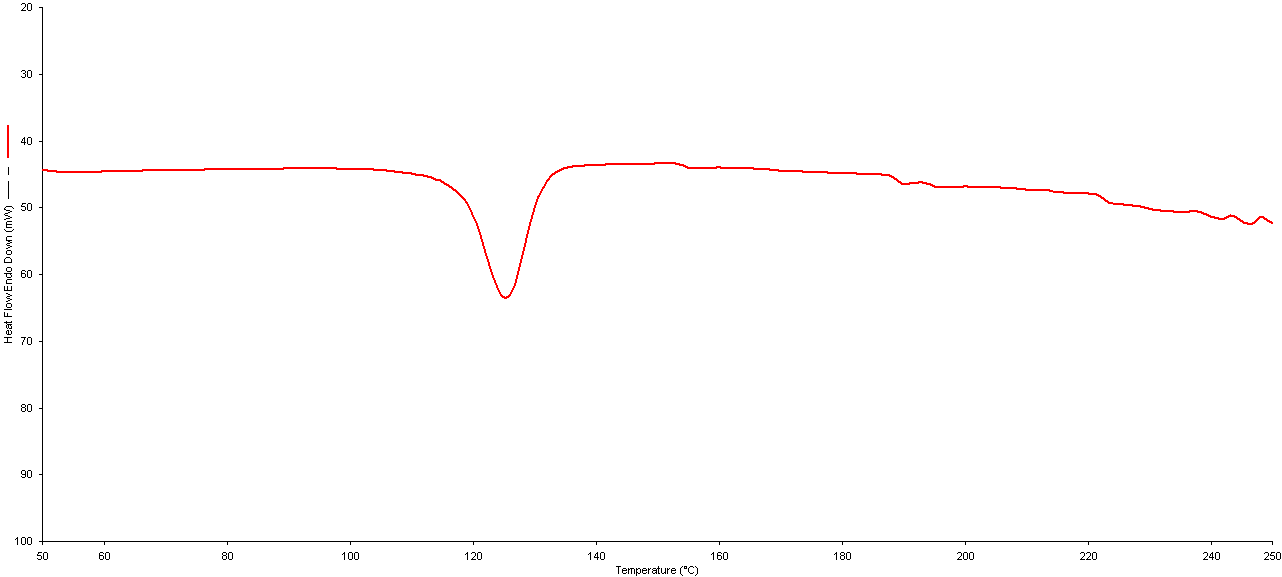


Solid 4a

Peak = 124 °C


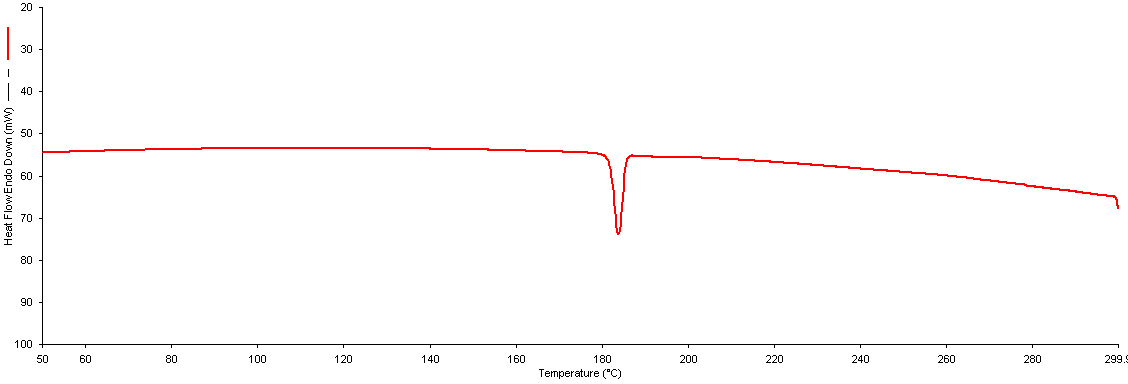


Solid 7

Peak = 188 °C


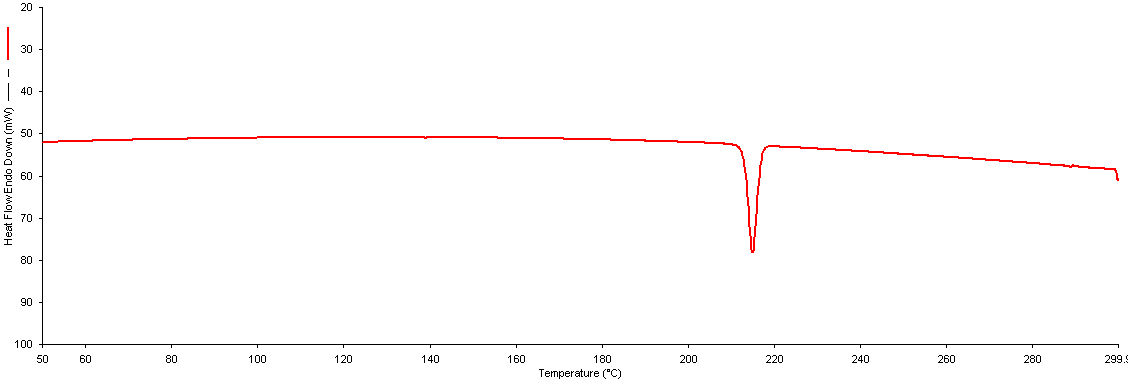


Solid 8

Peak = 216 °C


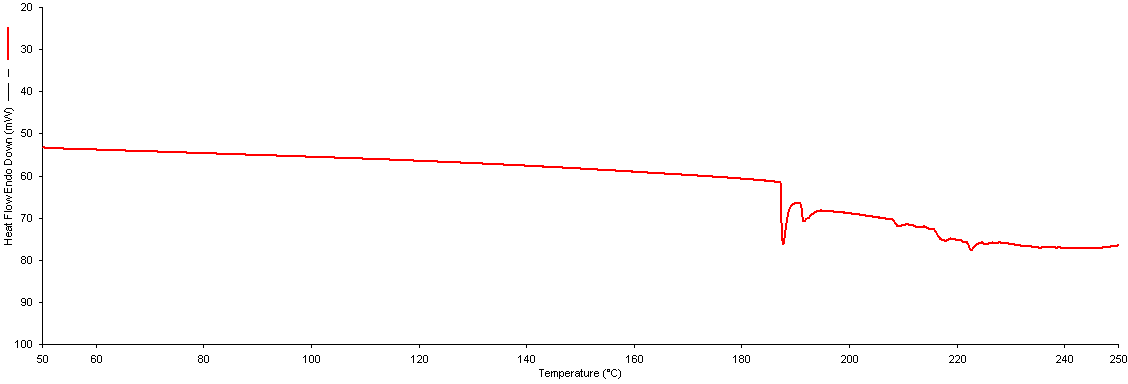


Solid 9

Peak = 192 °C


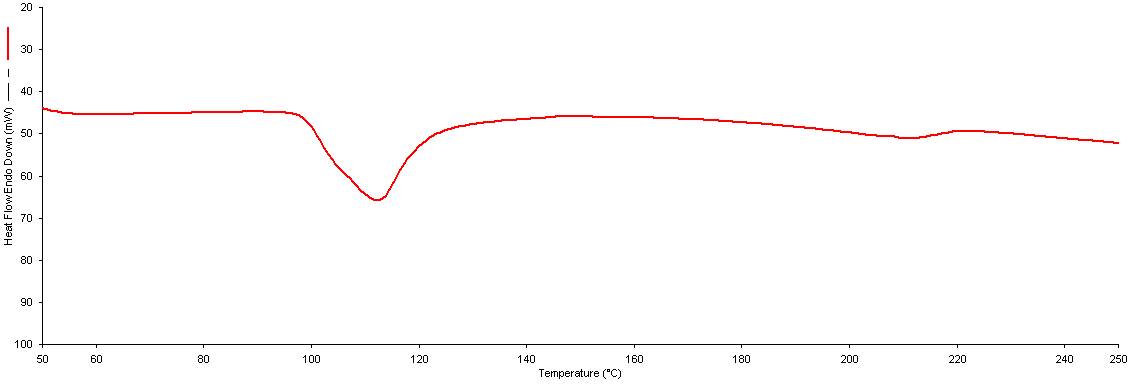


Solid 10

Peak = 118 °C

**
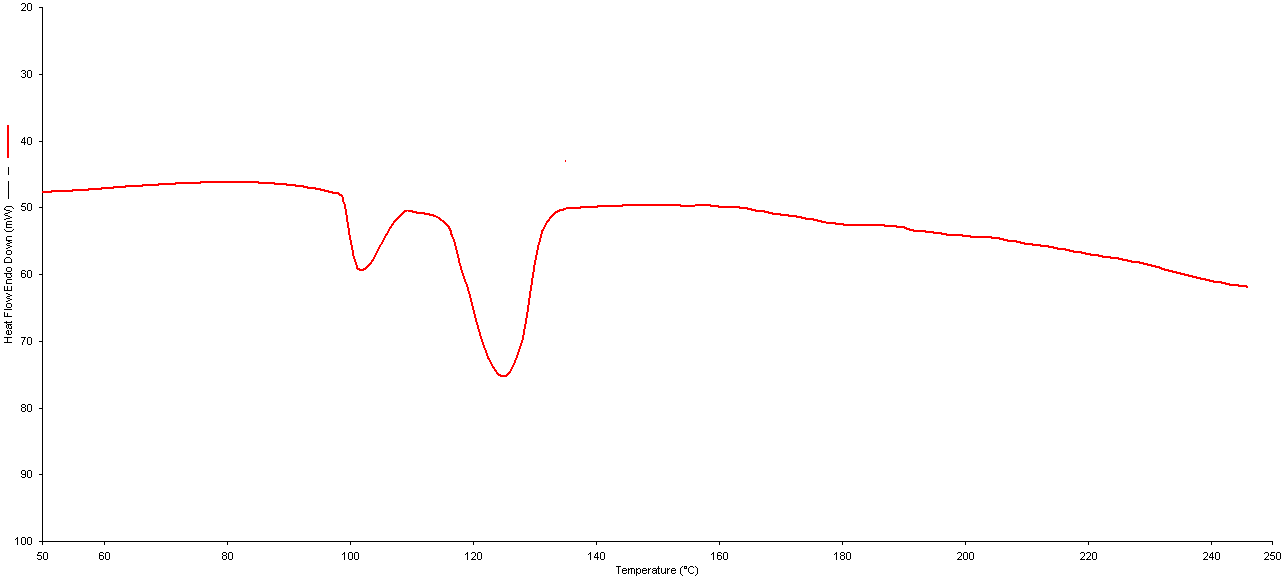
**

Solid 12

Peak = 125 °C

**Fig.S9.**DSC thermograms of solids **1** to**12**showing endothermic peaks corresponding to their melting point except for solids **2a,** which showed two endothermic peaks, first corresponding to removal of solvent and the second corresponding to the melting point.


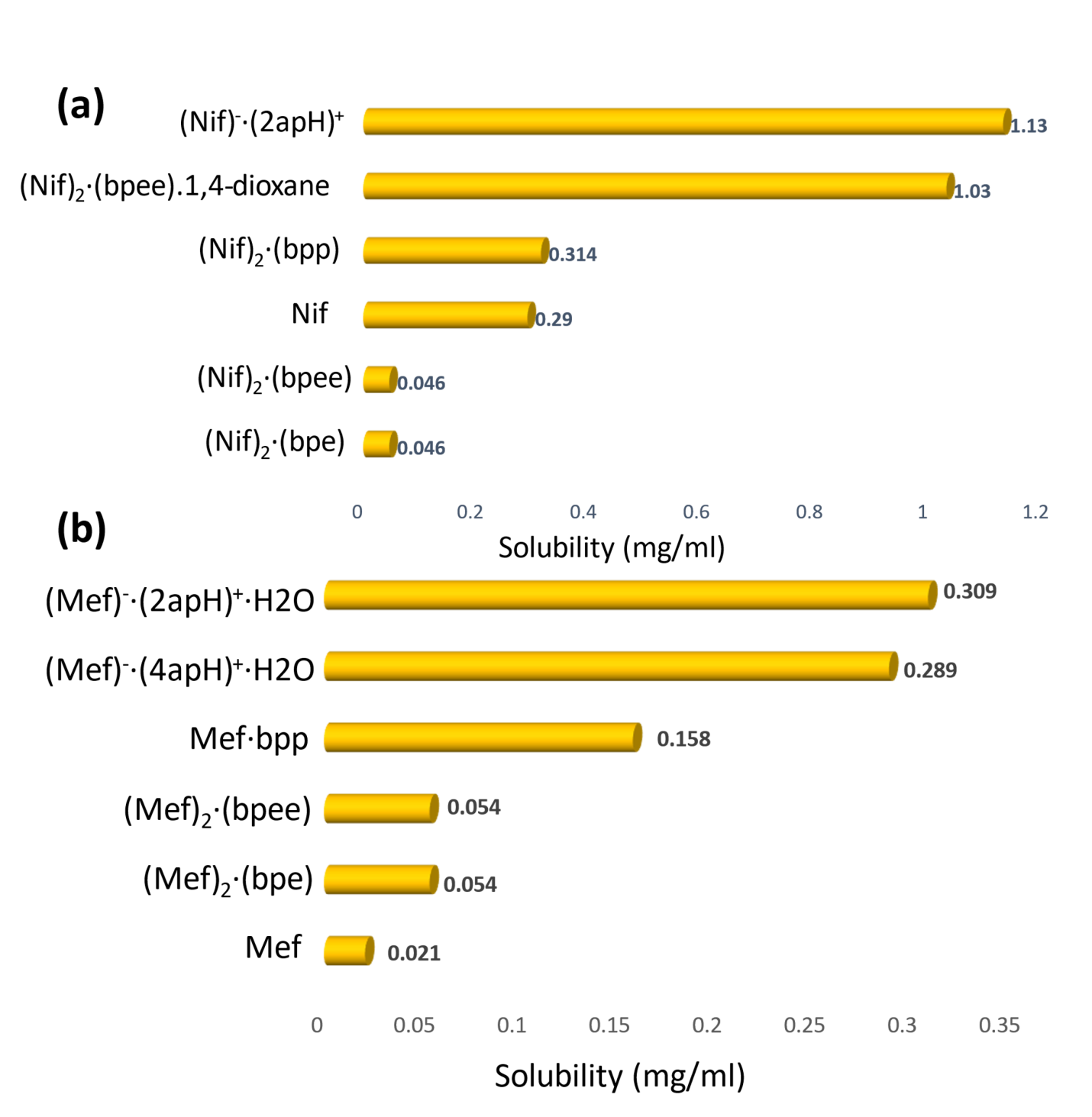


**Fig. S12.** Trends in (a) Nif and (b) Mef based multicomponent solids.

| Niflumic acid 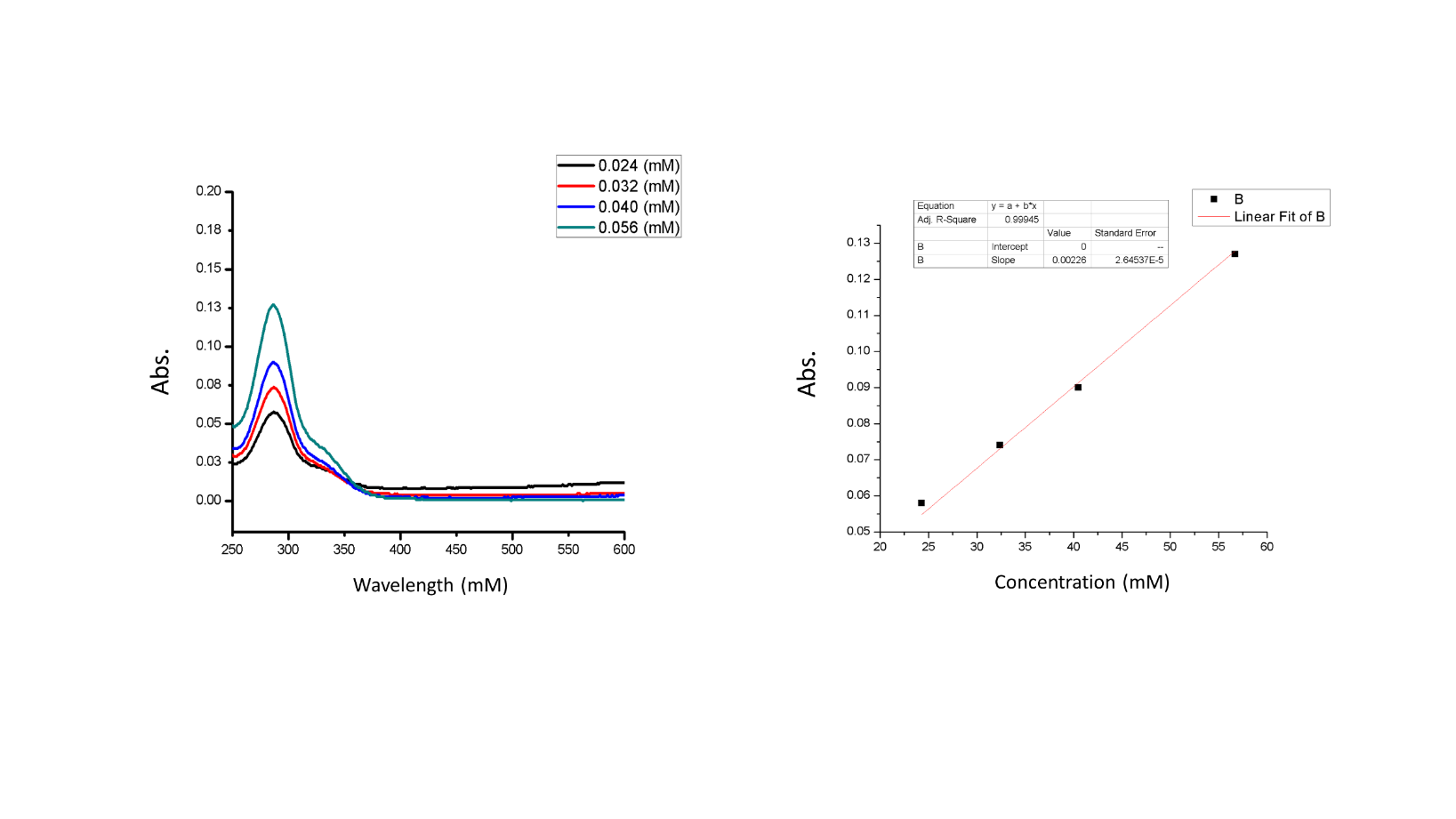 | 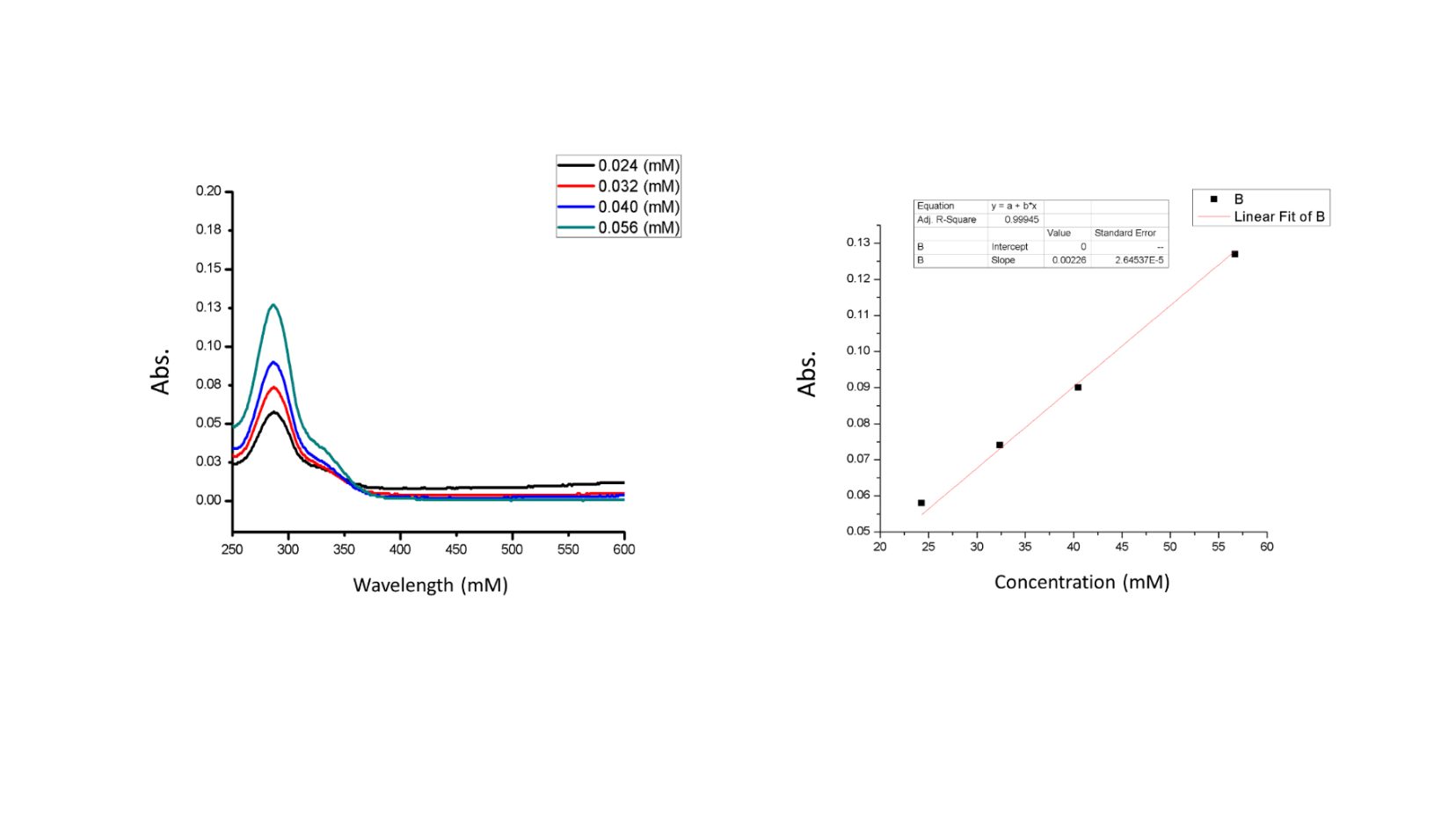 |
| --- | --- |
| Mefenamic acid 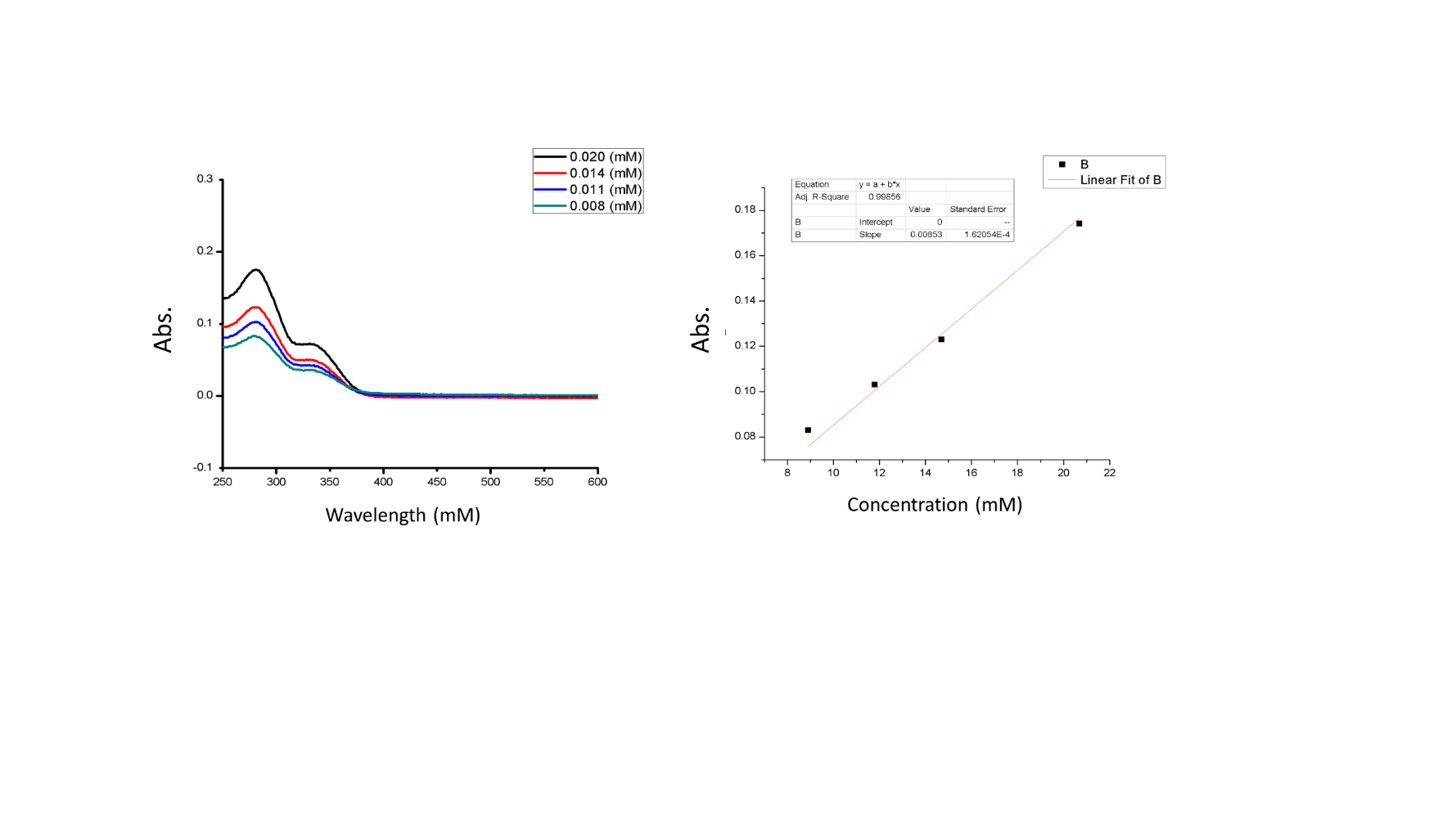 | 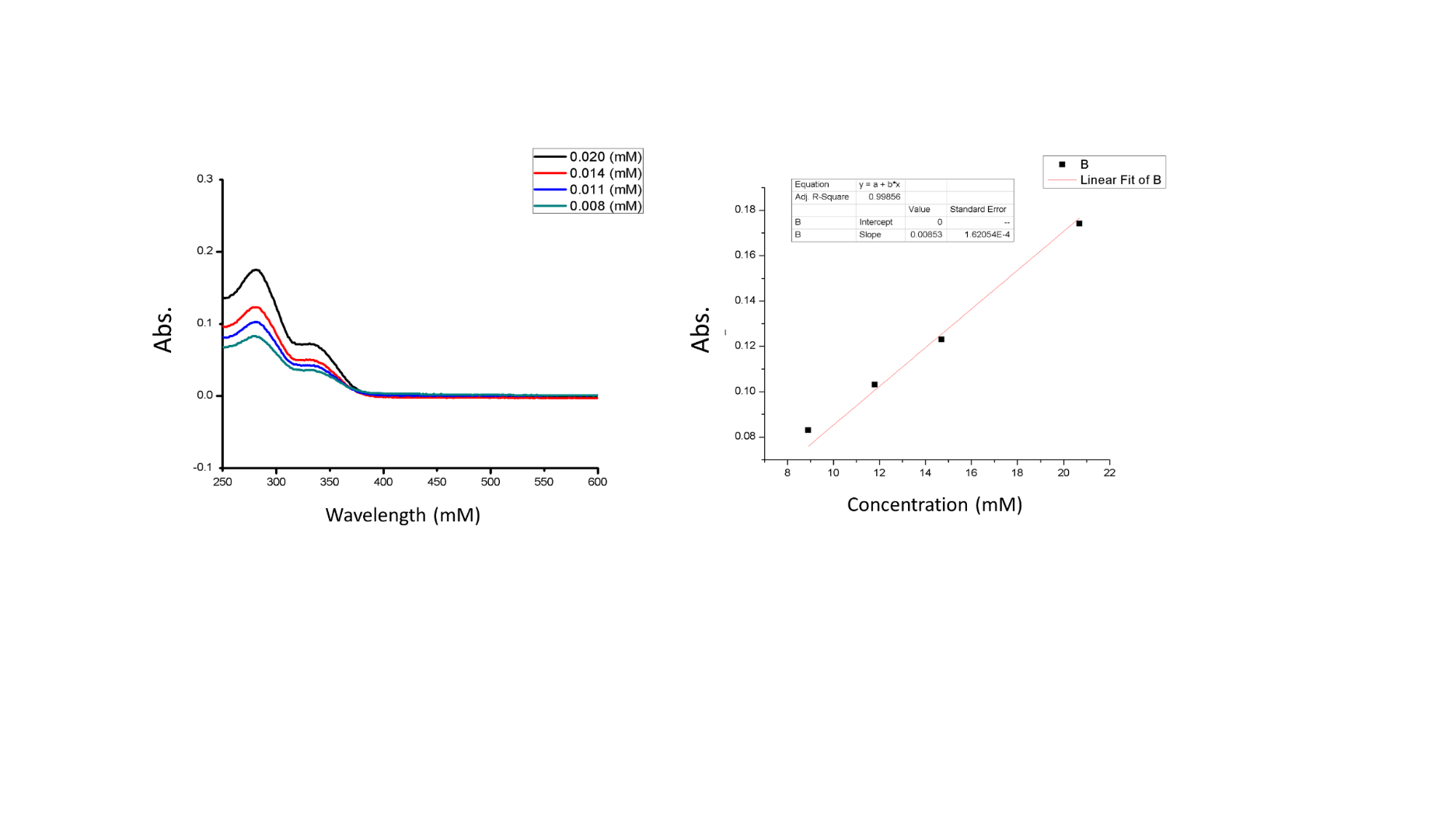 |
| Solid 1  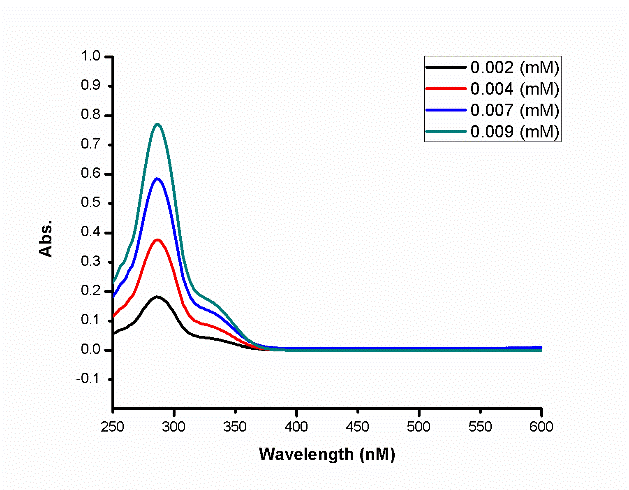 | 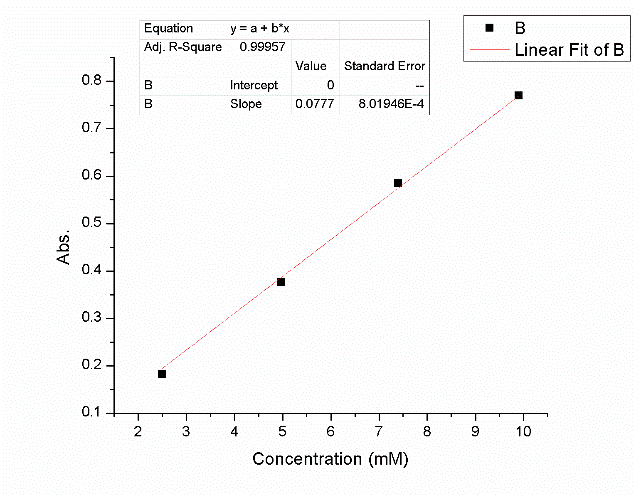 |
| Solid 2  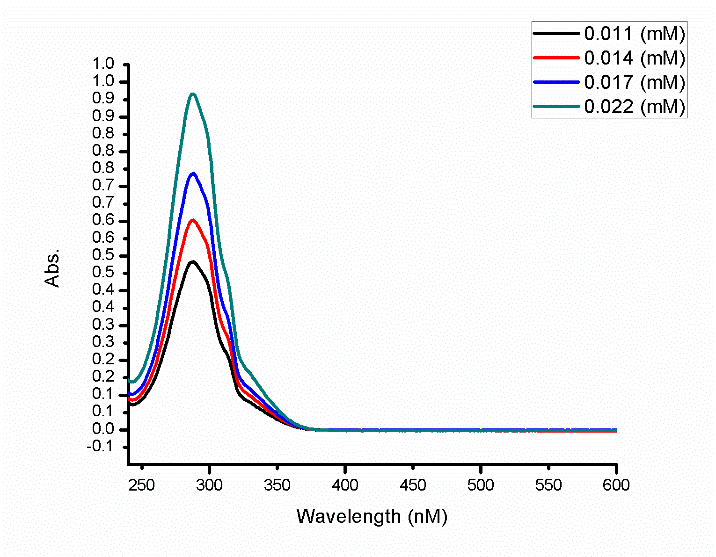 | 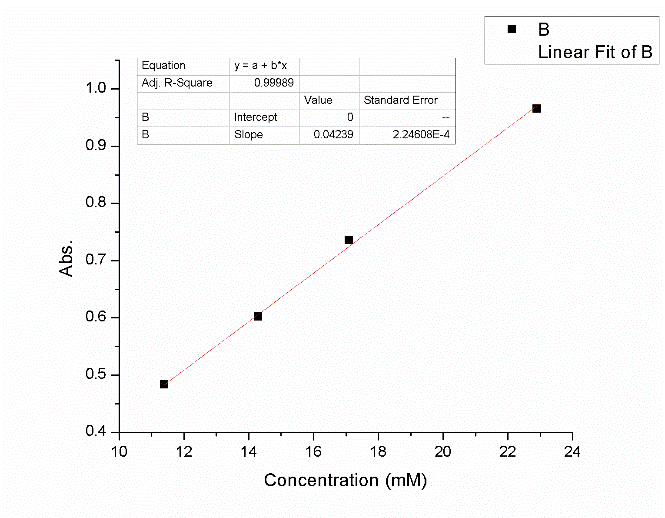 |
| Solid 2a  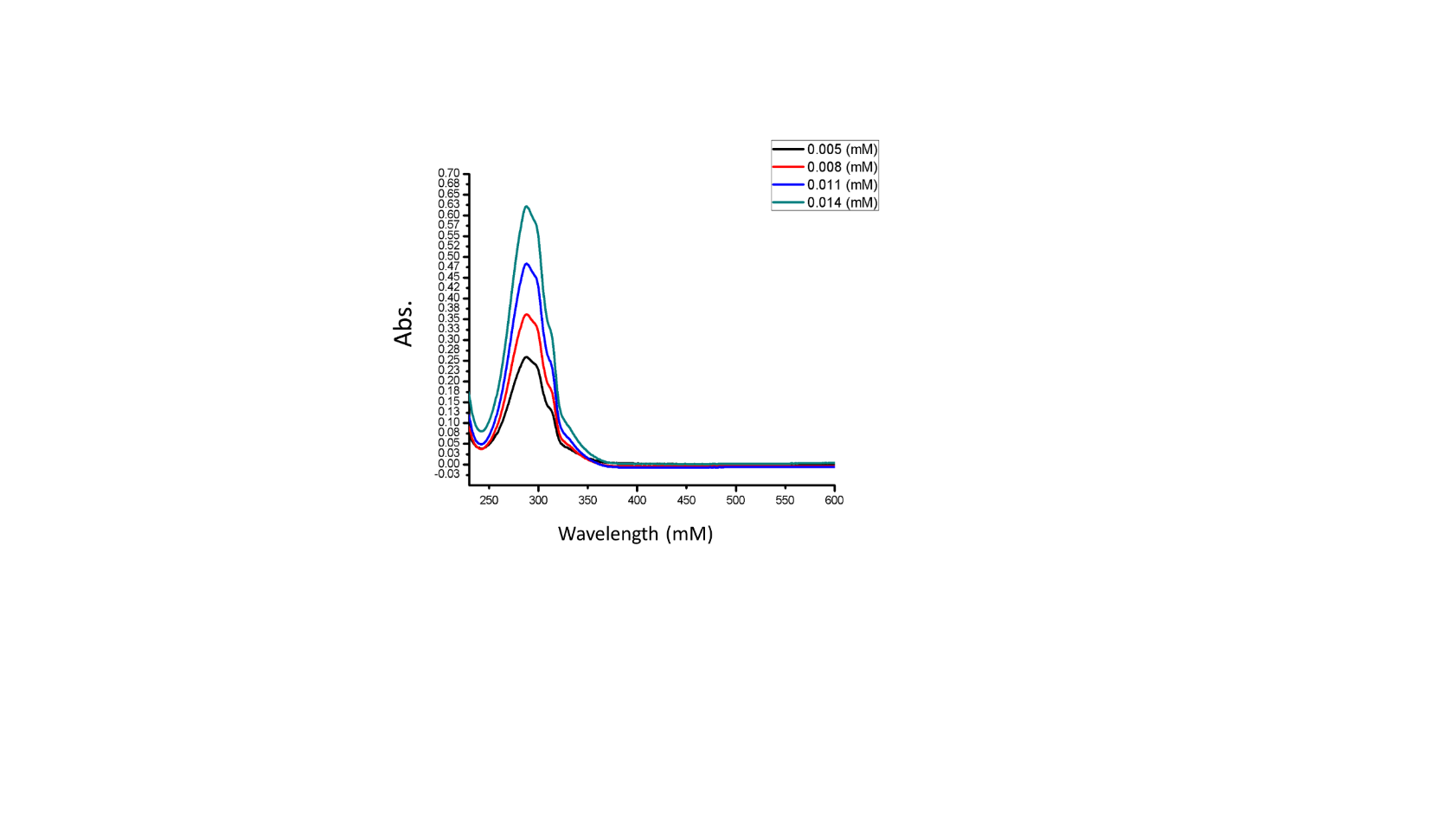 | 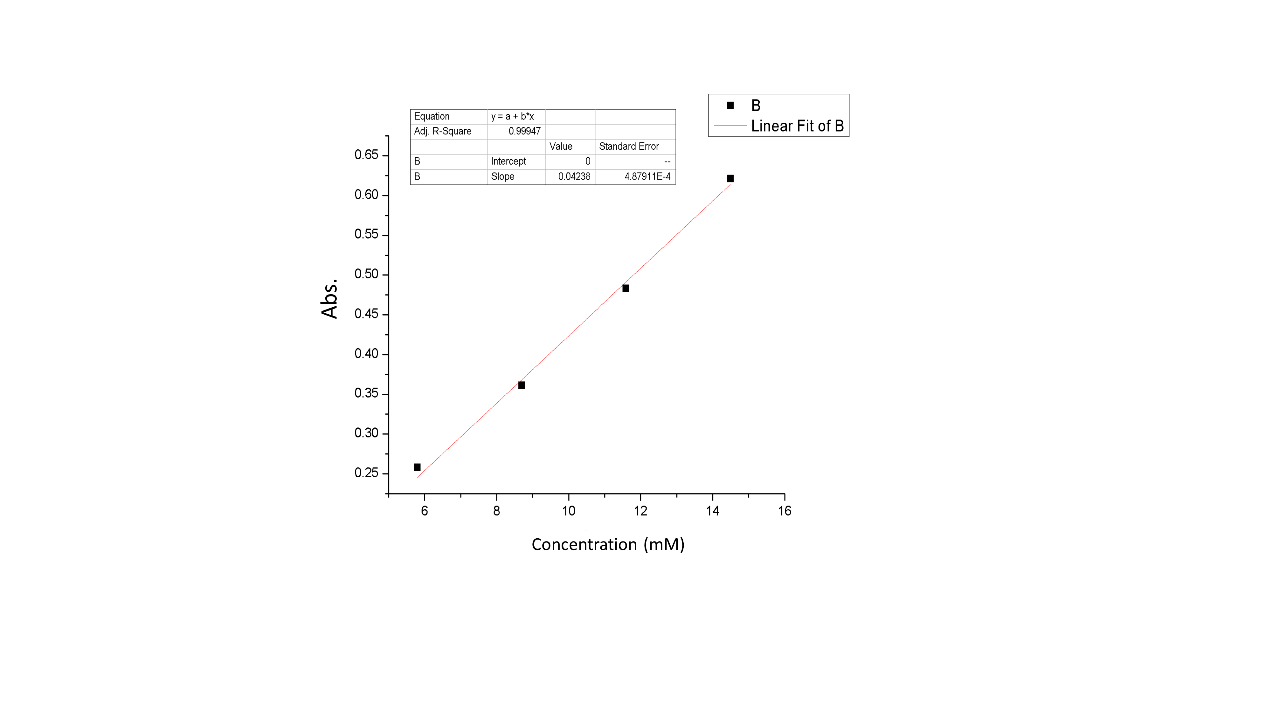 |
| Solid 3  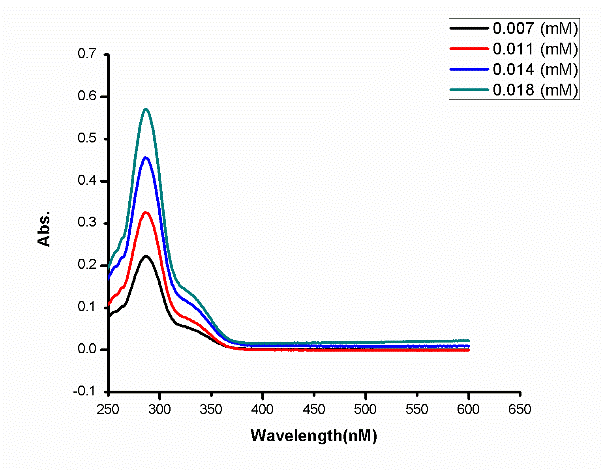 | 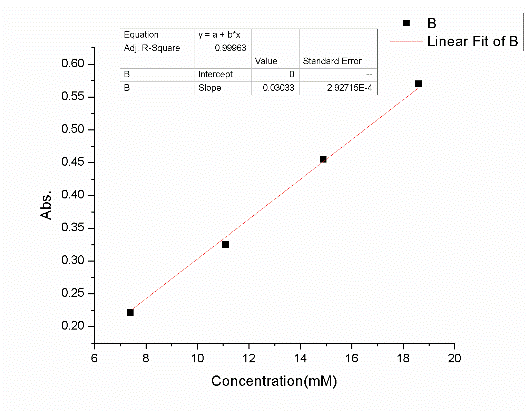 |
| Solid 4  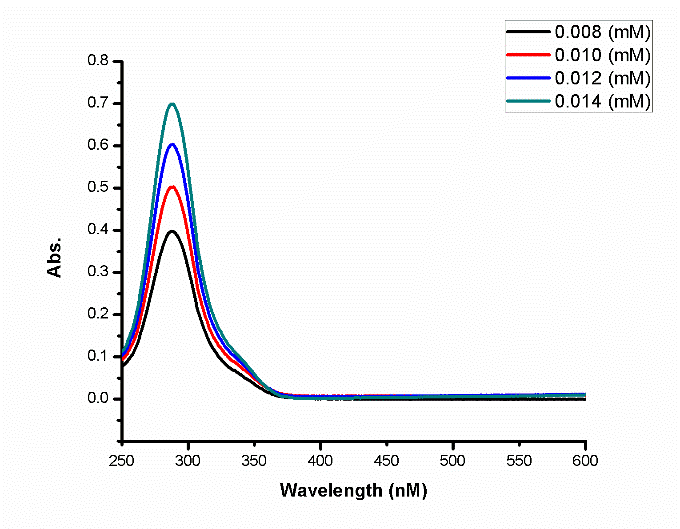 | 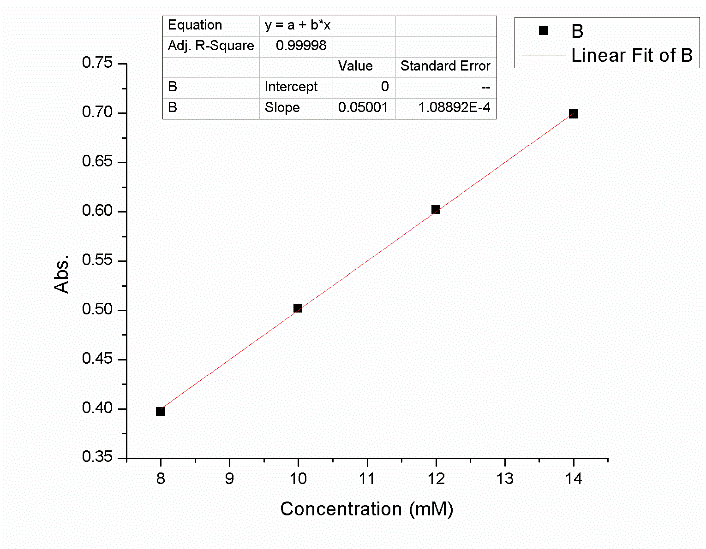 |
| Solid 7  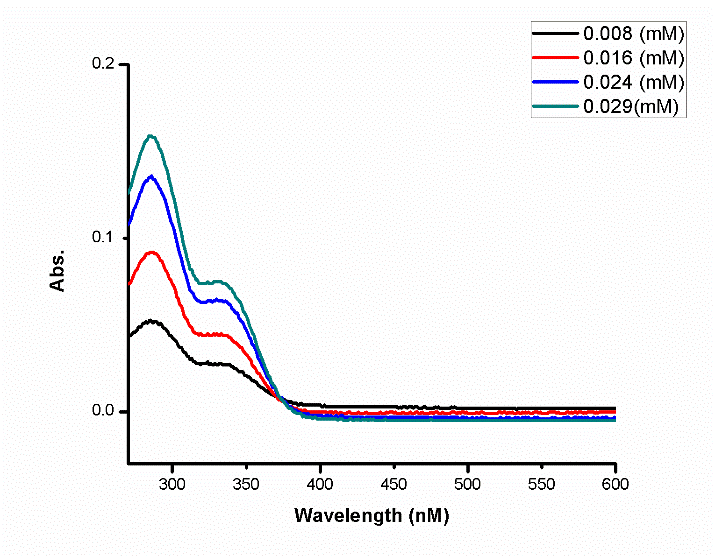 | 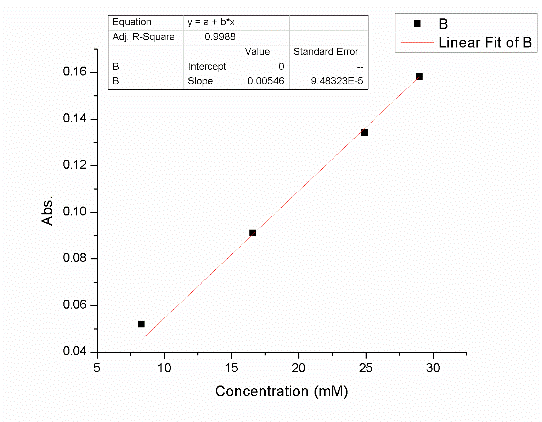 |
| Solid 8  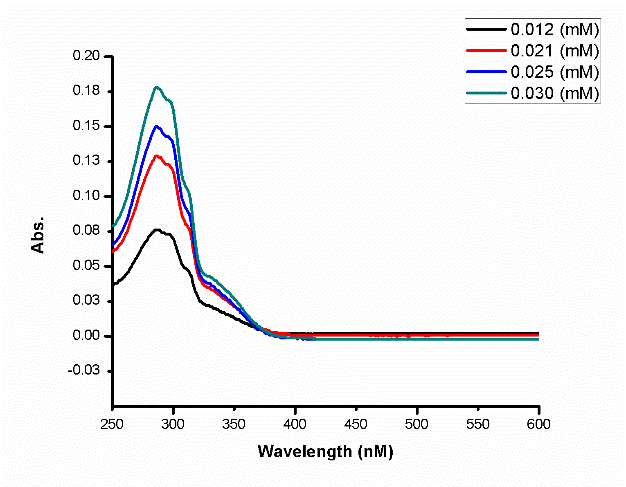 | 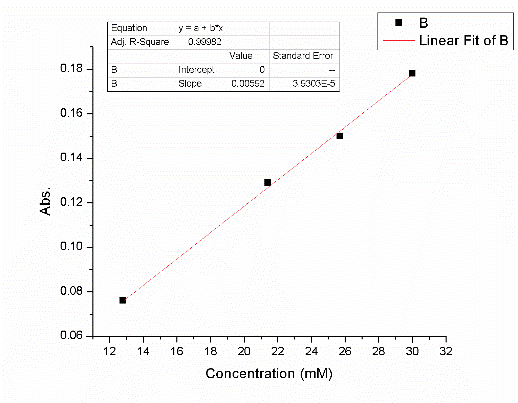 |
| Solid 9  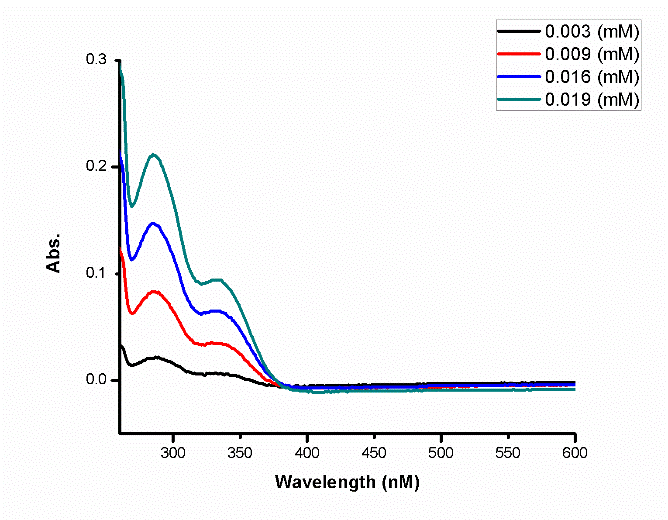 | 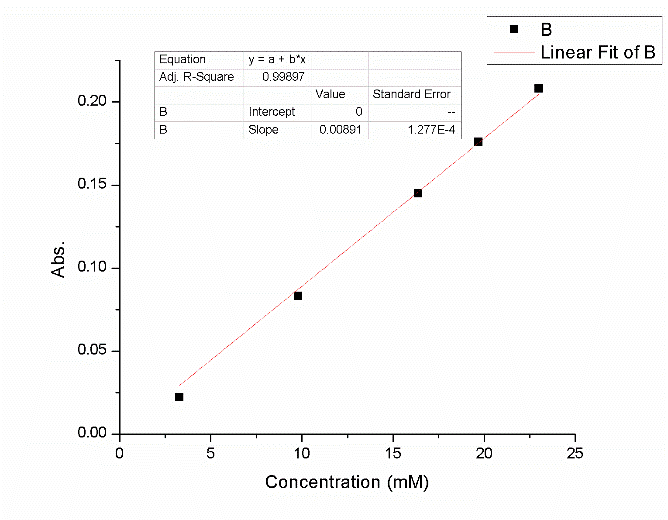 |
| Solid 10  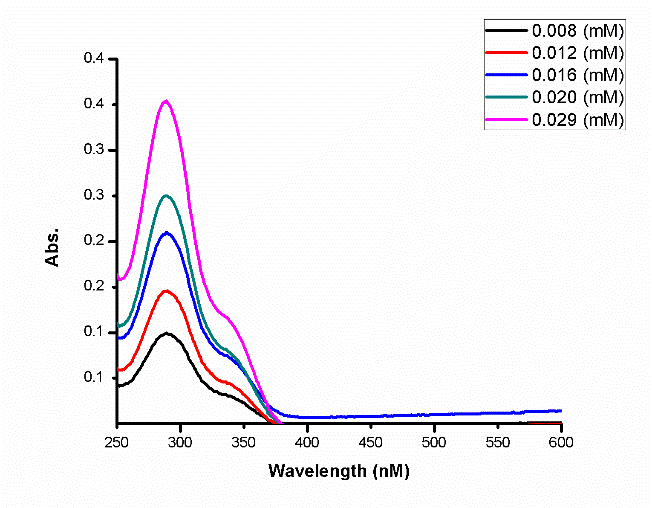 |  |
| Solid 12 |  |

**Fig. S13.** Absorbance vs. wavelength (left) and absorbance vs. concentration curves (right) for the standard solutions of solids reported in this study. Similar studies on solids 5, 6, and 11 could not be performed due to lack of purity and on solid 4a due to lack of stability in the samples.

**Table. S2.** Crystallization method used for the synthesis of Nif and Mef based multicomponent solids in the present study and their physical properties. Solids **5**, **6** and **11** could not be isolated as pure phases.

| **composition in the solid** | **Method and solvent of crystallization** | **color and morphology** | **m. p. (ºC) (from DSC)** |
| --- | --- | --- | --- |
| Nif | Used as received | Greenish | 203 |
| Mef | Used as received | colorless | 230 |
| (Nif)_2_·(bpe) (**1**) | Neat grinding, methanol assisted grinding, methanol | rod, colorless | 177 |
| (Nif)_2_·(bpee) (**2**) | Neat grinding, methanol assisted grinding, methanol | block, green | 179 |
| (Nif)_2_·(bpee).1,4-dioxane (**2a**) | 1,4- dioxane assisted grinding, 1,4- dioxane | rod, green | 178 |
| (Nif)_2_·(bpp) (**3**) | Neat grinding, methanol assisted grinding, methanol | rod, colorless | 92 |
| (Nif)^-^·(2apH)^+^(**4**) | Neat grinding, methanol assisted grinding, methanol | rod, yellow | 123 |
| (Nif)^-^·(2apH)^+^(**4a**) | Neat grinding, acetone assisted grinding, acetone | rod, yellow | 124 |
| (Nif)^-^·(3apH)^+^(**5**) | Neat grinding, methanol assisted grinding, methanol | rod, yellow |  |
| (Nif)^-^·(4apH)^+^(**6**) | Neat grinding, methanol assisted grinding, methanol | rod, colorless |  |
| (Mef)_2_·(bpe) (**7**) | Neat grinding, methanol assisted grinding, methanol | rod, colorless | 188 |
| (Mef)_2_·(bpee) (**8**) | Neat grinding, methanol assisted grinding, methanol | rod, green | 216 |
| Mef·bpp (**9**) | Neat grinding, methanol assisted grinding, methanol | block, colorless | 192 |
| (Mef)·(2apH)^+^·H_2_O (**10**) | Neat grinding, methanol assisted grinding, methanol | block, red | 118 |
| Mef·*3ap* (**11**) | Neat grinding, methanol assisted grinding, methanol | block, colorless |  |
| (Mef)^-^·(4apH)^+^·H_2_O (**12**) | Neat grinding, methanol assisted grinding, methanol | block, colorless | 125 |

**Table.S3.** The outcome of solids by p*K*a difference between Nif, Mef, and coformers.

| S. No. | NSAID | Coformer | Δp*K*a = [p*K*a(base) – p*K*a(acid)] | Solid form type | |
| --- | --- | --- | --- | --- | --- |
|  |  |  |  | Salt | Cocrystal |
| 1 | Mef | *bpee* | 4.99– 3.89=1.1 |  | **8** |
| 2 | Mef | *bpe* | 5.32–3.89=1.43 |  | **7** |
| 3 | Mef | *bpp* | 5.42–3.89=1.53 |  | **9** |
| 4 | Mef | *3ap* | 5.75– 3.89=1.86 |  | **11** |
| 5 | Mef | *2ap* | 6.82–3.89=2.93 | **10** |  |
| 6 | Nif | *bpee* | 4.99– 1.89=3.1 |  | **2, 2a** |
| 7 | Nif | *bpe* | 5.32–1.89=3.43 |  | **1** |
| 8 | Nif | *bpp* | 5.42– 1.89=3.53 |  | **3** |
| 9 | Nif | *3ap* | 5.75–1.89=3.86 | **5** |  |
| 10 | Nif | *2ap* | 6.82– 1.89=4.93 | **4, 4a** |  |
| 11 | Mef | *4ap* | 8.95–3.89=5.06 | **12** |  |
| 12 | Nif | *4ap* | 8.95–1.89=7.06 | **6** |  |

**Table.S4.** Torsional angles of Nif and Mef in the salt/cocrystal form and in the free state.

| Nif based solids | Nif | **1** | **2** | **2a** | **3** | **4** | **4a** | **5** | **6** |
| --- | --- | --- | --- | --- | --- | --- | --- | --- | --- |
| τ | –4.62 | 17.66,  –18.70 | 17.98 | 33.03 | 11.90 | –5.92 | 24.76 |  | 9.02,  –7.45 |
| Mef based solids | Mef I | Mef II | **7** | **8** | **9** | **10** | **11** | **12** |  |
| τ | –119.98 | -80.82 | 90.35 | -91.97 | 74.66 | 76.05 |  | 94.73 |  |

Table S5. Literature analysis of multicomponent solids based on fenamates in CCDC version 5.43 (November 2021) from the NSAID perspective. Compositions depicted in blue are reported as cocrystals while that in red are reported as salts. The CCDC code mentioned is hyperlinked to the [CCDC webpage](https://www.ccdc.cam.ac.uk/structures/?ccdc-check=2dbbdb1af729caf6f19d64fd625c52ed) for the corresponding structure. Solids with a composition apart from 1:1 are writtem with a yellow colour.

| **CCDC Code**  **Reference** | **Structure** | **Composition and**  **Lattice Parameters** |
| --- | --- | --- |
| **Fenamic Acid *(*H*fen)*** | | |
| [OYEBAX](https://www.ccdc.cam.ac.uk/structures/Search?Ccdcid=OYEBAX&DatabaseToSearch=Published)  (Jerzykiewicz, Sroka, and Majerz 2016) |  | **(H*fen)·(acd)***  *acd* = acridine  *P*2_1_/*c*  *a*=9.184Å *b*=17.224Å *c*=13.116Å β=106.08° V*=*1993.58Å^3^, Z*=*4 |
| [PEFCAG01](https://www.ccdc.cam.ac.uk/structures/Search?Ccdcid=PEFCAG01&DatabaseToSearch=Published)  (Surov et al. 2015)  (2:1) |  | **(H*fen)_2_·(bipy)***  *bipy* = 4,4'-bipyridine  *P*$\bar{1}$ *a*=7.760Å *b*=9.757Å *c*=20.063Å α*=*98.29° β=92.42° γ*=*104.59° V=1450.01Å^3^ Z*=*2 |
| [BEBGOH](https://www.ccdc.cam.ac.uk/structures/Search?Ccdcid=BEBGOH&DatabaseToSearch=Published)  (Sangeetha et al. 2017)  (2:1) |  | ***(fen)^-^(*H*trz)^+^ (trz)***  *trz =* 3-amino-5,6-dimethyl-1,2,4-triazine  *P*$\bar{1}$ *a*=7.501Å *b*=7.666Å *c*=21.433Å α*=*93.74° β=94.77° γ*=*106.19°  V=1174.52Å^3^, Z=2 |
| [OZEXAU](https://www.ccdc.cam.ac.uk/structures/Search?Ccdcid=OZEXAU&DatabaseToSearch=Published)  (Farhadikoutenaei and Ibrahim 2016) |  | ***(fen)^-^(*H*mpd)^+^***  *mpd* = 2-amino-5-methylpyridine  *P*2_1_/*c* *a*=17.379Å *b*=6.672Å *c*=14.387Å β=92.15° V*=*1667.32Å^3^ Z*=*4 |
| **Mefenamic Acid *(*H*mef)*** | | |
| [AFONIV](https://www.ccdc.cam.ac.uk/structures/Search?Ccdcid=AFONIV&DatabaseToSearch=Published)  (Zheng et al. 2018)  (2:1) |  | ***(*H*mef)_2_·(bpee)***  *bpee* = 1,2-bis(4-pyridyl)ethylene  *P*$\bar{1}$ *a=*7.819Å *b=*8.615Å *c=*12.912Å  α=98.21° β=99.51° γ=90.02° V*=*848.06Å^3^ Z*=*1 |
| [AFONOB](https://www.ccdc.cam.ac.uk/structures/Search?Ccdcid=AFONOB&DatabaseToSearch=Published)  (Zheng et al. 2018)  (2:1) |  | ***(*H*mef)_2_·(bpe)***  *bpe* = 1,2-bis(4-pyridyl)ethane  *P*$\bar{1}$ *a=*7.451Å *b=*7.835Å *c=*16.895Å  α=94.3° β=93.16° γ=117.89° V*=*864.61Å^3^ Z*=*1 |
| [AFONUH](https://www.ccdc.cam.ac.uk/structures/Search?Ccdcid=AFONUH&DatabaseToSearch=Published)  (Zheng et al. 2018)  (2:1) |  | ***(*H*mef)_2_·(azo)***  *azo* = 4,4'-azopyridine  *P*$\bar{1}$ *a=*7.549Å *b=*8.736Å *c=* 13.095Å  α=100.62° β=99.73° γ=93.92° V*=*832.17Å^3^ Z*=*1 |
| [AFOPAP](https://www.ccdc.cam.ac.uk/structures/Search?Ccdcid=AFOPAP&DatabaseToSearch=Published)  (Zheng et al. 2018) |  | ***(*H*mef)·(bpp)***  *bpp* = 4,4'-(propane-1,3-diyl)dipyridine  *P*$\bar{1}$ *a=*7.53Å *b=*7.84Å *c=*20.38Å  α=88.77° β=85.38° γ=79.99° V*=*1176.22Å^3^ Z*=*2 |
| [EXAQOK](https://www.ccdc.cam.ac.uk/structures/Search?Ccdcid=EXAQOK&DatabaseToSearch=Published)  (Fábián et al. 2011; Vaksler et al. 2021)  (1:2) |  | ***(*H*mef)·(nic)·(nic)***  *nic* = nicotinamide  *P*$\bar{1}$ *a=*4.064Å *b=*12.509Å *c=*24.088Å  α=99.89° β=90.73° γ=92.43° V*=*1205.15Å^3^ Z*=*2 |
| [MUPNEQ01](https://www.ccdc.cam.ac.uk/structures/Search?Ccdcid=MUPNEQ&DatabaseToSearch=Published)  (Pop et al. 2002) |  | ***(*H*mef)·(cyd)***  *cyd* = β-Cyclodextrin  *P*2_1_ *a=*15.479Å *b=*25.589Å *c=*9.297Å  β=98.97° V*=*3637.68Å^3^ Z*=*2 |
| [PORVEA](https://www.ccdc.cam.ac.uk/structures/Search?Ccdcid=PORVEA&DatabaseToSearch=Published)  (Bhattacharya et al. 2020) |  | ***(*H*mef) ^-^·(tmp) ^+^·H_2_O***  *tmp* = trimethoprim  *P*$\bar{1}$ *a=*9.360Å *b=*12.251Å *c=*13.876Å  α=98.25° β=107.41° γ=108.56° V*=*1388.40Å^3^ Z*=*2 |
| [UZUYIZ01](https://www.ccdc.cam.ac.uk/structures/Search?Ccdcid=UZUYIZ01&DatabaseToSearch=Published)  (Bouanga Boudiombo and Jacobs 2016) |  | ***(*H*mef)·(2mp)***  *2mp* = 2-methylpyridine  *P*2_1_/*n* *a=*7.785Å *b=*8.185Å *c=*27.953Å  β=96.87° V*=*1768.73Å^3^ Z*=*4 |
| [UZUYUL](https://www.ccdc.cam.ac.uk/structures/Search?Ccdcid=UZUYUL&DatabaseToSearch=Published)  (Bouanga Boudiombo and Jacobs 2016) |  | ***(*H*mef)·(3mp)***  *3mp* = 3-methylpyridine  *P*$\bar{1}$ *a=*7.673Å *b=*7.759Å *c=*16.272Å  α=78.95° β=83.67° γ=65.09° V*=*862.11Å^3^ Z*=*2 |
| [UZUZAS](https://www.ccdc.cam.ac.uk/structures/Search?Ccdcid=UZUZAS&DatabaseToSearch=Published)  (Bouanga Boudiombo and Jacobs 2016) |  | ***(*H*mef)·(3chp)***  *3chp* = 3-chloropyridine  *P*$\bar{1}$ *a=*7.671Å *b=*7.744Å *c=*16.056Å  α=80.06° β=84.88° γ=65.52° V*=*854.82Å^3^ Z*=*2 |
| [UZUZEW](https://www.ccdc.cam.ac.uk/structures/Search?Ccdcid=UZUZEW&DatabaseToSearch=Published)  (Bouanga Boudiombo and Jacobs 2016) |  | ***(*H*mef)·(4mp)***  *4mp* = 4-methylpyridine  *P*$\bar{1}$ *a=*7.527Å *b=*7.570Å *c=*15.661Å  α=88.11° β=78.41° γ=89.21° V*=*873.86Å^3^ Z*=*2 |
| [XOWKEB](https://www.ccdc.cam.ac.uk/structures/Search?Ccdcid=XOWKEB&DatabaseToSearch=Published)  (Surov et al. 2015; Wittering et al. 2015)  (2:1) |  | ***(*H*mef)_2_·(bipy)***  *bipy* = 4,4'-bipyridine  *P*$\bar{1}$ *a=*7.3086Å *b=*8.6692Å *c=*13.632Å  α=106.13° β=99.42° γ=98.75° V*=*800.46Å^3^ Z*=*1 |
| [ZAZGAK](https://www.ccdc.cam.ac.uk/structures/Search?Ccdcid=ZAZGAK&DatabaseToSearch=Published)  (SeethaLekshmi and Guru Row 2012) |  | ***(*H*mef)·(dmf)***  *dmf* = N,N-dimethylformamide  *P*$\bar{1}$ *a=*7.473Å *b=*9.559Å *c=*13.306Å  α=105.07° β=103.78° γ=103.41° V*=*846.51Å^3^ Z*=*2 |
| [JUDPIK](https://www.ccdc.cam.ac.uk/structures/Search?Ccdcid=JUDPIK&DatabaseToSearch=Published)  (Ranjan et al. 2020) |  | ***(mef)^-^(*H*map)^+^***  *map* = 4-(dimethylamino)pyridine  *P*$\bar{1}$ *a=*7.757Å *b=*9.472Å *c=*13.307Å  α=87.51° β=78.59° γ=74.17° V*=*922.20Å^3^ Z*=*2 |
| [JUDPUW](https://www.ccdc.cam.ac.uk/structures/Search?Ccdcid=JUDPUW&DatabaseToSearch=Published)  (Ranjan et al. 2020)  (2:1) |  | ***(Hmef) (mef)^-^(*H*map)^+^***  *map* = 4-(dimethylamino)pyridine  *P*$\bar{1}$ *a=*10.767Å *b=*11.967Å *c=*13.786Å  α=106.15° β=105.84° γ=103.49° V*=*1546.91Å^3^ Z*=*2 |
| [BINXOO](https://www.ccdc.cam.ac.uk/structures/Search?Ccdcid=BINXOO&DatabaseToSearch=Published)  (Bouanga Boudiombo and Jacobs 2018)  (2:1) |  | ***(mef)_2_^-^(*H_2_*eta)^2+^***  *eta* = ethane-1,2-diamine  *P*$\bar{1}$ *a=*6.795Å *b=*7.108Å *c=*16.056Å  α=93.56° β=100.86° γ=112.63° V*=*695.12Å^3^ Z*=*1 |
| [BINXUU](https://www.ccdc.cam.ac.uk/structures/Search?Ccdcid=BINXUU&DatabaseToSearch=Published)  (Bouanga Boudiombo and Jacobs 2018) |  | ***(mef)^-^(*H*tea)^+^***  *tea* = triethylamine  *Pbca* *a=*11.201Å *b=*15.206Å *c=*22.491Å  V*=*3830.72Å^3^ Z*=*8 |
| [BINYAB](https://www.ccdc.cam.ac.uk/structures/Search?Ccdcid=BINYAB&DatabaseToSearch=Published)  (Bouanga Boudiombo and Jacobs 2018) |  | ***(mef)^-^(*H*mep)^+^***  *mep* = 4-methylpiperazine  *P*21/c *a=*7.593Å *b=*15.389Å *c=* 15.791Å  β=91.96° V*=*1844.87Å^3^ Z*=*4 |
| [BINYEF](https://www.ccdc.cam.ac.uk/structures/Search?Ccdcid=BINYEF&DatabaseToSearch=Published)  (Bouanga Boudiombo and Jacobs 2018) |  | ***(mef)^-^(*H*mor)^+^***  *mor* = morpholinium  *P*$\bar{1}$ *a=*7.825Å *b=*9.889Å *c=*12.291Å  α=79.1° β=73.65° γ=74.14° V*=*871.38Å^3^ Z*=*2 |
| [GIMXIM](https://www.ccdc.cam.ac.uk/structures/Search?Ccdcid=GIMXIM&DatabaseToSearch=Published)  (Bhattacharya et al. 2018) |  | ***(mef)^-^(*H*cefo)^+^***  *cefo* = 4-(3-carboxy-1-ethyl-6-fluoro-4-oxo-1,4-dihydroquinolin-7-yl) piperazine  *P*$\bar{1}$ *a=*7.062Å *b=*13.631Å *c=*15.304Å  α=73.35° β=77.59° γ=80.51° V*=*1369.71Å^3^ Z*=*2 |
| [PUHVAR](https://www.ccdc.cam.ac.uk/structures/Search?Ccdcid=PUHVAR&DatabaseToSearch=Published)  (Roy et al. 2014) |  | ***(mef)^-^(*H*ada)^+^***  *ada* = adamantan-1-amine  *P*$\bar{1}$ *a=*13.007Å *b=*14.017Å *c=*14.409Å  α=62.98° β=80.33° γ=66.67° V*=*2149.26Å^3^ Z*=*4 |
| [RUVNEC01](https://www.ccdc.cam.ac.uk/structures/Search?Ccdcid=RUVNEC01&DatabaseToSearch=Published)  (Roy and Dastidar 2017) |  | ***(mef)^-^(*H*dhpa)^+^·H_2_O***  *dhpa* = 1,3-dihydroxy-2-(hydroxymethyl) propan-2-aminium  *P*$\bar{1}$ *a=*7.796Å *b=*8.461Å *c=*15.224Å  α=90.29° β=98.03° γ=97.29° V*=*985.91 Å^3^ Z*=*2 |
| [RUVNIG](https://www.ccdc.cam.ac.uk/structures/Search?Ccdcid=RUVNIG&DatabaseToSearch=Published)  (Fonari et al. 2010)  (2:1) |  | ***(mef) ^-^_2_ (aacd)^2+^·2H_2_O***  *aacd* = 4,10-Diaza-1,7-diazoniacyclododecane  *C*2/*c* *a=*32.809Å *b=*7.659Å *c=*29.684Å  β=104.45° V*=*7223.31Å^3^ Z*=*8 |
| [RUVNOM](https://www.ccdc.cam.ac.uk/structures/Search?Ccdcid=RUVNOM&DatabaseToSearch=Published)  (Fonari et al. 2010)  (2:1) |  | ***(mef)^-^_2_(*H_2_*pip)^2+^·4H_2_O***  *pip* = Piperazine  *P*$\bar{1}$ *a=*6.929Å *b=*7.174Å *c=*18.621Å  α=85.99° β=83.24° γ=65.81° V*=*838.35Å^3^ Z*=*1 |
| [RUVNUS](https://www.ccdc.cam.ac.uk/structures/Search?Ccdcid=RUVNUS&DatabaseToSearch=Published)  (Fonari et al. 2010)  (2:1) |  | ***(mef_2_)^-^(*H_2_*maacd)^2+^·2H_2_O***  *maacd* = (5R,12S)-5,7,7,12,14,14-Hexamethyl-4,11-diaza-1,8-diazoniacyclotetradecane  *P*$\bar{1}$ *a=*8.565Å *b=*8.718Å *c=*16.021Å  α=102.38° β=91.41° γ=112.49° V*=*1071.97Å^3^ Z*=*1 |
| [SOFYOE](https://www.ccdc.cam.ac.uk/structures/Search?Ccdcid=SOFYOE&DatabaseToSearch=Published)  (Wang et al. 2019)  (2:1) |  | ***(mef)^-^_2_(*H_2_*pip)^2+^***  *pip* = piperazine  *Pbca* *a=*10.186Å *b=*7.577Å *c=*38.191Å  V*=*2947.59Å^3^ Z*=*4 |
| [SOGVIW](https://www.ccdc.cam.ac.uk/structures/Search?Ccdcid=SOGVIW&DatabaseToSearch=Published)  (Wang et al. 2019) |  | ***(mef)^-^(*H*pip)^+^***  *pip* = piperazine  *P*2_1_/*c* *a=*7.536Å *b=*8.149Å *c=*28.44Å  β=94.93° V*=*1740.31Å^3^ Z*=*4 |
| [TAMCES](https://www.ccdc.cam.ac.uk/structures/Search?Ccdcid=TAMCES&DatabaseToSearch=Published)  (Nechipadappu and Trivedi 2017) |  | ***(mef)^-^(*H*4ap)^+^·H_2_O***  *4ap* = 4-aminopyridine  *P*2_1_/*n* *a=*7.760Å *b=*8.352Å *c=*28.623Å  β=95.31° V*=*1847.21Å^3^ Z*=*4 |
| [TAMCIW01](https://www.ccdc.cam.ac.uk/structures/Search?Ccdcid=TAMCIW01&DatabaseToSearch=Published)  (Nechipadappu and Trivedi 2017; Ranjan et al. 2020) |  | ***(mef)^-^(*H*map)^+^·H_2_O***  *map* = 4-(dimethylamino)pyridine  *P*$\bar{1}$ *a=*7.8149Å *b=*8.1357Å *c=*16.3674Å  α=79.58° β=81.33° γ=89.95° V*=*1011.43Å^3^ Z*=*2 |
| [TEQCEA](https://www.ccdc.cam.ac.uk/structures/Search?Ccdcid=TEQCEA&DatabaseToSearch=Published)  (Roy and Dastidar 2017) |  | ***(mef)^-^(*H*mpa)^+^***  *mpa* = 2-methylpropan-2-amine  *P*$\bar{1}$ *a=*13.193Å *b=*16.947Å *c=*18.601Å  α=65.29° β=79.79° γ=78.50° V*=*3681.68Å^3^ Z*=*8 |
| [ZAZGEO](https://www.ccdc.cam.ac.uk/structures/Search?Ccdcid=ZAZGEO&DatabaseToSearch=Published)  (SeethaLekshmi and Guru Row 2012)  (1:2) |  | ***(mef)^-^(*H*ohpi)^+^ (*H*ohpi)***  *ohpi* = 2-Oxo-2,3-dihydropyrimidin-4-imine  *P*$\bar{1}$ *a=*6.966Å *b=*7.321Å *c=*23.801Å  α=86.95° β=83.94° γ=67.85° V*=*1117.82Å^3^ Z*=*2 |
| **Tolfenamic Acid *(*H*tol)*** | | |
| [JUDNUU](https://www.ccdc.cam.ac.uk/structures/Search?Ccdcid=JUDNUU&DatabaseToSearch=Published)  (Ranjan et al. 2020)  (2:1) |  | ***(*H*tol)_2_·(map)***  *map* = 4-(dimethylamino)pyridine  *P*$\bar{1}$ *a=*10.886Å *b=*12.271Å *c=*13.781Å  α=106.97° β=105.78° γ=103.32° V*=*1595.53Å^3^ Z*=*2 |
| [JUDPEG](https://www.ccdc.cam.ac.uk/structures/Search?Ccdcid=JUDPEG&DatabaseToSearch=Published)  (Ranjan et al. 2020) |  | ***(*H*tol)·(dmf)***  *dmf* = N, N-dimethylformamide  *P*$\bar{1}$ *a=*10.480Å *b=*11.842Å *c=*13.331Å  α=94.33° β=95.88° γ=102.87° V*=*1596.16Å^3^ Z*=*4 |
| [EXAQIE](https://www.ccdc.cam.ac.uk/structures/Search?Ccdcid=EXAQIE&DatabaseToSearch=Published)  (Fábián et al. 2011)  (1:2) |  | ***(*H*tol)·(nic).(nic)***  *nic =* nicotinamide  *P*$\bar{1}$ *a=*4.006Å *b=*12.554Å *c=*24.126Å  α=100.32° β=90.40° γ=92.53° V*=*1192.55Å^3^ Z*=*2 |
| [PORVAW](https://www.ccdc.cam.ac.uk/structures/Search?Ccdcid=PORVAW&DatabaseToSearch=Published)  (Bhattacharya et al. 2020) |  | ***(tol)^-^(*H*tmp)^+^·H_2_O***  *tmp =* trimethoprim  *P*$\bar{1}$ *a=*9.351Å *b=*12.216Å *c=*13.934Å  α=98.43° β=108.31° γ=108.26° V*=*1381.14Å^3^ Z*=*2 |
| [UZUZIA](https://www.ccdc.cam.ac.uk/structures/Search?Ccdcid=UZUZIA&DatabaseToSearch=Published)  (Bouanga Boudiombo and Jacobs 2016) |  | ***(*H*tol)·(2mp)***  *2mp* = 2-methylpyridine  *P*2_1_/*n* *a=*7.758Å *b=*8.069Å *c=*28.056Å  β=97.291° V*=*1742.39Å^3^ Z*=*4 |
| [UZUZOG](https://www.ccdc.cam.ac.uk/structures/Search?Ccdcid=UZUZOG&DatabaseToSearch=Published)  (Bouanga Boudiombo and Jacobs 2016) |  | ***(*H*tol)·(3mp)***  *3mp* = 3-methylpyridine  *Pbca* *a=*7.806Å *b=*13.987Å *c=*32.185Å  V*=*3514.44Å^3^ Z*=*8 |
| [XOWKAX01](https://www.ccdc.cam.ac.uk/structures/Search?Ccdcid=XOWKAX01&DatabaseToSearch=Published)  (Wittering et al. 2015)  (2:1) |  | ***(*H*tol)_2_·(bipy)***  *bipy =* 4,4'-bipyridine  *P*2_1_/*c* *a=*4.708Å *b=*45.219Å *c=*7.918Å  β=106.89° V*=*1613.12Å^3^ Z*=*2 |
| TAMCUI  (Nechipadappu and Trivedi 2017; Ranjan et al. 2020) |  | ***(tol)^-^(*H*map)^+^·H_2_O***  *map* = 4-(dimethylamino)pyridinium  *P*$\bar{1}$ *a=*7.848Å *b=*8.103Å *c=*16.309Å  α=100.58° β=98.03° γ=90.38° V*=*1008.98Å^3^ Z*=*2 |
| [JUDNII](https://www.ccdc.cam.ac.uk/structures/Search?Ccdcid=JUDNII&DatabaseToSearch=Published)  (Ranjan et al. 2020) |  | ***(tol)^-^(*H*map)^+^***  *map* = 4-(dimethylamino)pyridine  *P*$\bar{1}$ *a=*7.929Å *b=*9.322Å *c=*13.586Å  α=87.77° β=76.93° γ=76.03° V*=*949.23Å^3^ Z*=*2 |
| [PUHTAP](https://www.ccdc.cam.ac.uk/structures/Search?Ccdcid=PUHTAP&DatabaseToSearch=Published)  (Roy et al. 2014) |  | ***(tol)^-^(*H*ada)^+^·sol***  *ada* = adamantan-1-amine  *P*2_1_/*c* *a=*6.629Å *b=*19.332Å *c=*18.807Å  β=95.45° V*=*2399.40Å^3^ Z*=*4 |
| [SOGNOU](https://www.ccdc.cam.ac.uk/structures/Search?Ccdcid=SOGNOU&DatabaseToSearch=Published)  (Wang et al. 2019)  (2:1) |  | ***(tol)^-^_2_(*H_2_*pip)^2+^***  *pip* = piperazine  *Pbca* *a=*10.303Å *b=*7.617Å *c=*38.517Å  V*=*3022.85Å^3^ Z*=*4 |
| [SOGVOC](https://www.ccdc.cam.ac.uk/structures/Search?Ccdcid=SOGVOC&DatabaseToSearch=Published)  (Wang et al. 2019) |  | ***(tol)^-^(*H*pip)^+^***  *pip* = piperazine  *P*2_1_/*n* *a=*10.827Å *b=*7.502Å *c=*21.322Å  β=97.39° V*=*1717.49Å^3^ Z*=*4 |
| [TAMCOC](https://www.ccdc.cam.ac.uk/structures/Search?Ccdcid=TAMCOC&DatabaseToSearch=Published)  (Nechipadappu and Trivedi 2017) |  | ***(tol)^-^(*H*4ap)^+^·H_2_O***  *4ap* = 4-aminopyridine  *P*2_1_/*n* *a=*7.774Å *b=*8.326Å *c=*28.369Å  β=95.17° V*=*1829.04Å^3^ Z*=*4 |
| [TERLIO](https://www.ccdc.cam.ac.uk/structures/Search?Ccdcid=TERLIO&DatabaseToSearch=Published)  (Roy and Dastidar 2017) |  | ***(tol)^-^(*H*dhpa)^+^***  *dhpa* = 1,3-dihydroxy-2-(hydroxymethyl) propan-2-amine  *P*$\bar{1}$ *a=*10.811Å *b=*12.625Å *c=*14.418Å  α=92.07° β=91.31° γ=109.01° V*=*1858.068Å^3^ Z*=*4 |
| [YAWLAM](https://www.ccdc.cam.ac.uk/structures/Search?Ccdcid=YAWLAM&DatabaseToSearch=Published)  (Parveen, Sravanthi, and Dastidar 2017) |  | ***(tol)^-^(*H*phe)^+^***  *phe* = phenylethanamine  *Pcc*2 *a=*15.389Å *b=*17.224Å *c=*7.555Å  V*=*2002.17Å^3^ Z*=*4 |
| [YAWLEQ](https://www.ccdc.cam.ac.uk/structures/Search?Ccdcid=YAWLEQ&DatabaseToSearch=Published)  (Parveen, Sravanthi, and Dastidar 2017) |  | ***(tol)^-^(*H*het)^+^***  *het* = 2-(4-hydroxyphenyl)ethylamine  *I*2/*a* *a=*18.332Å *b=*7.701Å *c=*28.664Å  β=102.35° V*=*3957.59Å^3^ Z*=*8 |
| [YAWLIU](https://www.ccdc.cam.ac.uk/structures/Search?Ccdcid=YAWLIU&DatabaseToSearch=Published)  (Parveen, Sravanthi, and Dastidar 2017) |  | ***(tol)^-^(*H*hpa)^+^***  *hpa* = 1,3-dihydroxypropan-2-amine  *P*2_1_/*c* *a=*17.927Å *b=*7.772Å *c=*12.562Å  β=104.181° V*=*1696.47Å^3^ Z*=*4 |
| **Meclofenamic Acid *(*H*mec)*** | | |
| [SAXPAK](https://www.ccdc.cam.ac.uk/structures/Search?Ccdcid=SAXPAK&DatabaseToSearch=Published)  (Sanphui, Bolla, and Nangia 2012) |  | ***(*H*mec)·(inic)***  *inic* = isonicotinamide  *P*2_1_/*c* *a=*7.532Å *b=*31.313Å *c=*9.601Å  α=90° β=121.44° γ=90° V*=*1932.14Å^3^ Z*=*4 |
| [SAXPEO](https://www.ccdc.cam.ac.uk/structures/Search?Ccdcid=SAXPEO&DatabaseToSearch=Published)  (Sanphui, Bolla, and Nangia 2012)  (2:1) |  | ***(*H*mec)_2_·(bipy)***  *bipy* = 4,4'-bipyridine  *P*2_1_/*c* *a=*7.418Å *b=*8.214Å *c=*28.771Å  α=90° β=97.09° γ=90° V*=*1739.64Å^3^ Z*=*4 |
| [FOPMIG](https://www.ccdc.cam.ac.uk/structures/Search?Ccdcid=FOPMIG&DatabaseToSearch=Published)  (Venugopal Dhanaraj and Vijayan 1987) |  | ***(mec)^-^(*H*eth)^+^***  *eth = ethanolamine*  *P*$\bar{1}$ *a=*9.232Å *b=*12.287Å *c=*17.033Å  α=70.21° β=76.72° γ=68.21° V*=*1676.09Å^3^ Z*=*4 |
| [FOPMOM](https://www.ccdc.cam.ac.uk/structures/Search?Ccdcid=FOPMOM&DatabaseToSearch=Published)  (Venugopal Dhanaraj and Vijayan 1987) |  | ***(mec)^-^(*H*cho)^+^·H_2_O***  *cho* ***=* c**holine  *Pna*2_1_ *a=*9.637Å *b=*12.962Å *c=*33.099Å  V*=*4134.55Å^3^ Z*=*8 |
| [PUHTUJ](https://www.ccdc.cam.ac.uk/structures/Search?Ccdcid=PUHTUJ&DatabaseToSearch=Published)  (Roy et al. 2014) |  | ***(mec)^-^(*H*ada)^+^***  *ada* ***=*** adamantan-1-amine  *P*$\bar{1}$ *a=*12.545Å *b=*13.937Å *c=*15.612Å  α=111.47° β=111.53° γ=94.37° V*=*2291.91Å^3^ Z*=*4 |
| [SAXPOY01](https://www.ccdc.cam.ac.uk/structures/Search?Ccdcid=SAXPOY01&DatabaseToSearch=Published)  (Sanphui, Bolla, and Nangia 2012) |  | ***(mec)^-^(*H*pip)^+^***  *pip* *=* piperazine  *P*2_1_2_1_2_1_ *a=*7.929Å *b=*8.168Å *c=*28.381Å  V*=*1838.07Å^3^ Z*=*4 |
| [SAXQAL](https://www.ccdc.cam.ac.uk/structures/Search?Ccdcid=SAXQAL&DatabaseToSearch=Published)  (Sanphui, Bolla, and Nangia 2012) |  | ***(mec)^-^(*H*pip)^+^·H_2_O***  *pip* ***=*** piperazine  *P*2_1_/*c* *a=*16.137Å *b=*8.488Å *c=*15.596Å  β=114.94° V*=*1937.21Å^3^ Z*=*4 |
| [SAXQEP](https://www.ccdc.cam.ac.uk/structures/Search?Ccdcid=SAXQEP&DatabaseToSearch=Published)  (Sanphui, Bolla, and Nangia 2012)  (2:1) |  | ***(mec)^-^_2_(*H_2_*pip)^2+^***  *pip* ***=*** piperazine  *P*$\bar{1}$ *a=*7.910Å *b=*11.128Å *c=*18.118Å  α=81.94° β=80.42° γ=88.81° V*=*1557.34Å^3^ Z*=*4 |
| [SAXQIT](https://www.ccdc.cam.ac.uk/structures/Search?Ccdcid=SAXQIT&DatabaseToSearch=Published)  (Sanphui, Bolla, and Nangia 2012) |  | ***(mec)^-^(*H*2ap)^+^***  *2ap* ***=*** 2-aminopyridine  *P*2_1_/*n* *a=*14.675Å *b=*7.132Å *c=*18.605Å  α=90° β=110.73° γ=90° V*=*1821.12Å^3^ Z*=*4 |
| [SAXQOZ](https://www.ccdc.cam.ac.uk/structures/Search?Ccdcid=SAXQOZ&DatabaseToSearch=Published)  (Sanphui, Bolla, and Nangia 2012) |  | ***(mec)^-^(*H*4ap)^+^·H_2_O***  *4ap* = 4-aminopyridine  *P*2_1_/*c* *a=*16.227Å *b=*8.477Å *c=*15.686Å  α=90° β=116.18° γ=90° V*=*1936.35Å^3^ Z*=*4 |
| [TEQCOK](https://www.ccdc.cam.ac.uk/structures/Search?Ccdcid=TEQCOK&DatabaseToSearch=Published)  (Roy and Dastidar 2017) |  | ***(mec)^-^(*H*mpa)^+^***  *mpa* = 2-methylpropan-2-amine  *C*2/*c* *a=*27.003Å *b=*10.628Å *c=*28.498Å  β=106.73° V*=*7831.86Å^3^ Z*=*16 |
| [TERLEK](https://www.ccdc.cam.ac.uk/structures/Search?Ccdcid=TERLEK&DatabaseToSearch=Published)  (Roy and Dastidar 2017) |  | ***(mec)^-^(*H*dhpa)^+^·CH_3_OH***  *dhpa* = 1,3-dihydroxy-2-(hydroxymethyl) propan-2-amine  *Cc* *a=*10.828Å *b=*12.782Å *c=*32.954Å  β=99.26° V*=*4501.52Å^3^ Z*=*8 |
| **Flufenamic Acid *(*H*flu)*** | | |
| [DARNOC01](https://www.ccdc.cam.ac.uk/structures/Search?Ccdcid=DARNOC01&DatabaseToSearch=Published)  (Bhattacharya et al. 2020) |  | ***(*H*flu)·(smz)***  smz = sulfamethazine  *Pbca* *a=*15.931Å *b=*14.710Å *c=*21.885Å V*=*5129.08Å^3^ Z*=*8 |
| [EXAQAW](https://www.ccdc.cam.ac.uk/structures/Search?Ccdcid=EXAQAW&DatabaseToSearch=Published)  (Fábián et al. 2011) |  | ***(*H*flu)·(nic)***  *nic* = nicotinamide  *P*2_1_/*c* *a=*5.105Å *b=*15.961Å *c=*22.119Å  β=90.47° V*=*1802.35Å^3^ Z*=*4 |
| [FAQWUS](https://www.ccdc.cam.ac.uk/structures/Search?Ccdcid=FAQWUS&DatabaseToSearch=Published)  (Nechipadappu, Tekuri, and Trivedi 2017) |  | ***(*H*flu)·(cnba)***  *cnba* = 2-chloro-4-nitrobenzoic acid  *P*2_1_/*c* *a=*7.633Å *b=*32.137Å *c=*8.440Å  β=95.17° V*=*2062.16Å^3^ Z*=*4 |
| [FAQXAZ](https://www.ccdc.cam.ac.uk/structures/Search?Ccdcid=FAQXAZ&DatabaseToSearch=Published)  (Nechipadappu, Tekuri, and Trivedi 2017) |  | ***(*H*flu)·(eth)***  *eth* = 2-ethoxybenzamide  *P*$\bar{1}$ *a=*7.628Å *b=*8.807Å *c=*17.427Å  α=103.78° β=94.74° γ=102.94° V*=*1096.74Å^3^ Z*=*2 |
| [PORTUO](https://www.ccdc.cam.ac.uk/structures/Search?Ccdcid=PORTUO&DatabaseToSearch=Published)  (Bhattacharya et al. 2020) |  | ***(flu)^-^(*H*tmp)^+.^H_2_O***  *tmp* ***=*** trimethoprim  *P*$\bar{1}$ *a=*9.605Å *b=*12.238Å *c=*13.671Å  α=99.63° β=106.04° γ=109.05° V*=*1400.04Å^3^ Z*=*2 |
| [ZIQDUA](https://www.ccdc.cam.ac.uk/structures/Search?Ccdcid=ZIQDUA&DatabaseToSearch=Published)  (Aitipamula et al. 2014) |  | ***(*H*flu)·(mhp)***  *mhp* = 1,3-dimethyl-3,7-dihydro-1H-purine-2,6-dione  *P*$\bar{1}$ *a=*6.993Å *b=*10.127Å *c=*15.112Å  α=95.46° β=96.7° γ=101.17° V*=*1035.13Å^3^ Z*=*2 |
| [ZIQFAI](https://www.ccdc.cam.ac.uk/structures/Search?Ccdcid=ZIQFAI&DatabaseToSearch=Published)  (Aitipamula et al. 2014) |  | ***(*H*flu)·(pyo)***  *pyo* = pyridin-2-one  *P*$\bar{1}$ *a=*7.562Å *b=*7.645Å *c=*14.552Å  α=90.45° β=92.14° γ=95.81° V*=*836.39Å^3^ Z*=*2 |
| [ZIQFEM02](https://www.ccdc.cam.ac.uk/structures/Search?Ccdcid=ZIQFEM02&DatabaseToSearch=Published)  (Wittering et al. 2015)  (2:1) |  | ***(*H*flu)_2_·(bipy)***  *bipy* = 4,4'-bipyridine  *P*$\bar{1}$ *a=*9.829Å *b=*10.383Å *c=*24.990Å  α=84.68° β=81.33° γ=71.77° V*=*2392.05Å^3^ Z*=*3 |
| [SOZGAR](https://www.ccdc.cam.ac.uk/structures/Search?Ccdcid=SOZGAR&DatabaseToSearch=Published)  (Roy et al. 2014) |  | ***(flu)^-^(*H*ada)^+^***  *ada* = adamantan-1-amine  *P*2_1_/*c* *a=*11.337Å *b=*6.526Å *c=*29.981Å  β=98.849° V*=*2191.972Å^3^ Z*=*4 |
| [TEQCUQ](https://www.ccdc.cam.ac.uk/structures/Search?Ccdcid=TEQCUQ&DatabaseToSearch=Published)  (Roy and Dastidar 2017) |  | ***(flu)^-^(*H*mpa)^+^***  *mpa* = 2-methylpropan-2-amine  *P*2_1_/c *a=*14.752Å *b=*6.492Å *c=*19.455Å  β=103.58° V*=*1811.08Å^3^ Z*=*4 |
| [TEQYEW](https://www.ccdc.cam.ac.uk/structures/Search?Ccdcid=TEQYEW&DatabaseToSearch=Published)  (Roy and Dastidar 2017) |  | ***(flu)^-^(*Hd*hpa)^+^·H_2_O***  *dhpa* = 1,3-dihydroxy-2-(hydroxymethyl) propan-2-amine  *P*$\bar{1}$ *a=*9.063Å *b=*10.821Å *c=*20.661Å  α=97.36° β=96.44° γ=92.68° V*=*1992.98Å^3^ Z*=*4 |
| **Niflumic Acid *(Hnif)*** | | |
| [CAGXEN](https://www.ccdc.cam.ac.uk/structures/Search?Ccdcid=CAGXEN&DatabaseToSearch=Published)  (V. Dhanaraj and Vijayan 1983) |  | ***(nif)^-^(*H*het)^+^· H_2_O***  *eth* = ethanolamine  *P*$\bar{1}$ *a=6.239*Å *b=11.280*Å *c=12.451*Å  α=101.21° β=92.32° γ=99.93°  V*=844.31*Å^3^ Z*=2* |
| [DARGEL](https://www.ccdc.cam.ac.uk/structures/Search?Ccdcid=DARGEL&DatabaseToSearch=Published)  (Kumar et al. 2017; Bhattacharya et al. 2020) |  | ***(*H*nif)·(smz)***  *smz* = sulfamethazine  *Pbca* *a=15.840*Å *b=14.613*Å *c=21.829*Å  V*=5052.76*Å^3^ Z*=8* |
| [EXAQEA](https://www.ccdc.cam.ac.uk/structures/Search?Ccdcid=EXAQEA&DatabaseToSearch=Published)  (Fábián et al. 2011) |  | ***(*H*nif)·(nic)***  *nic* = nicotinamide  *P*2_1_/*c* *a=15.152*Å *b=5.060*Å *c=24.674*Å  β=112°  V*=1751.72*Å^3^ Z*=4* |
| [RIQWUM](https://www.ccdc.cam.ac.uk/structures/Search?Ccdcid=RIQWUM&DatabaseToSearch=Published)  (Mittapalli et al. 2019)  nif gets protonated |  | ***(H_2_nif)^+^(bsa*)^‑^**  *bsa* = Benzenesulfonic acid  *P*$\bar{1}$  *a=7.889*Å *b=8.074*Å *c=16.868*Å  α=84.05° β=87.49° γ=61.69° V*=940.79*Å^3^ Z*=2* |
| [RIQXAT](https://www.ccdc.cam.ac.uk/structures/Search?Ccdcid=RIQXAT&DatabaseToSearch=Published)  (Mittapalli et al. 2019) |  | ***(*H*nif)·(azp)***  *azp* = azepan-2-one  *P*$\bar{1}$ *a=5.451*Å *b=10.478*Å *c=16.891*Å  α=89.61° β=85.51° γ=86.69° V*=960.14*Å^3^ Z*=2* |
| [RIQXEX](https://www.ccdc.cam.ac.uk/structures/Search?Ccdcid=RIQXEX&DatabaseToSearch=Published)  (Mittapalli et al. 2019) |  | ***(*H*nif)·(pyo)***  *pyo* = pyridin-2(1H)-one *P*$\bar{1}$ *a=7.207*Å *b=10.314*Å *c=11.889*Å  α=100.34° β=101.77° γ=90.48° V*=850.17*Å^3^ Z*=2* |
| [XOWKIF](https://www.ccdc.cam.ac.uk/structures/Search?Ccdcid=XOWKIF&DatabaseToSearch=Published)  (Surov et al. 2015)  (2:1) |  | ***(*H*nif)_2_·(bipy)***  *bipy* = 4,4'-bipyridine  *C*2/*c* *a=26.001*Å *b=6.739*Å *c=19.854*Å  β=111.38°  V*=3239.37*Å^3^ Z*=4* |
| [KIGMIX](https://www.ccdc.cam.ac.uk/structures/Search?Ccdcid=KIGMIX&DatabaseToSearch=Published)  (Childs, Stahly, and Park 2007)  nif gets protonated |  | ***(H_2_nif)^+^(mal*)^‑^**  H*mal* = maleic acid  *P*2_1_/*c*  *a=8.143*Å *b=20.756*Å *c=10.651*Å  β=100.276° V*=1688.73*Å^3^ Z*=4* |
| [LIRSEN](https://www.ccdc.cam.ac.uk/structures/Search?Ccdcid=LIRSEN&DatabaseToSearch=Published)  (Surov et al. 2018) |  | ***(*H*nif)·(pro)***  *pro* = L-proline  *P*2_1_  *a=11.807*Å *b=5.812*Å *c=13.348*Å  β=97.84°  V*=907.51*Å^3^ Z*=2* |
| [RIQXIB](https://www.ccdc.cam.ac.uk/structures/Search?Ccdcid=RIQXIB&DatabaseToSearch=Published)  (Mittapalli et al. 2019)  (2:1) |  | ***(nif)^-^_2_(*H_2_*pip)^2+^***  *pip* = piperazine  *R*$\bar{3}$ *a=38.266*Å *b=38.266*Å *c=5.999*Å  V*=7607.64*Å^3^ Z*=9* |
| [RIQXOH](https://www.ccdc.cam.ac.uk/structures/Search?Ccdcid=RIQXOH&DatabaseToSearch=Published)  (Mittapalli et al. 2019) |  | ***(nif)^-^(*H*het)^+^***  *het* =2-(4-hydroxyphenyl)ethylamine  *P*2_1_/*c* *a=13.806*Å *b=8.090*Å *c=17.873*Å  β=93.66°  V*=1992.17*Å^3^ Z*=4* |
| [RIQXUN](https://www.ccdc.cam.ac.uk/structures/Search?Ccdcid=RIQXUN&DatabaseToSearch=Published)  [RIQXUN01](https://www.ccdc.cam.ac.uk/structures/Search?Ccdcid=RIQXUN&DatabaseToSearch=Published)  (Mittapalli et al. 2019) |  | ***(nif)^-^(Hphm*)^+^**  *phm* = phenylmethanamine  *P*$\bar{1}$ *a=4.731*Å *b=10.913*Å *c=18.451*Å  α=75.255° β=85.065° γ=82.971° V*=912.75*Å^3^ Z*=2* |
| [VAKVOU](https://www.ccdc.cam.ac.uk/structures/Search?Ccdcid=VAKVOU&DatabaseToSearch=Published)  (Lemmerer et al. 2010) |  | ***(nif)^-^(H*pea)^+^**  *pea* = (R)-1-Phenylethylammine  *P*2_1_  *a=12.416*Å *b=5.554*Å *c=28.162*Å  β=94.21° V*=1936.57*Å^3^ Z*=2* |

**References**

Aitipamula, Srinivasulu, Annie B H Wong, Pui Shan Chow, and Reginald B H Tan. 2014. “Cocrystallization with Flufenamic Acid: Comparison of Physicochemical Properties of Two Pharmaceutical Cocrystals.” *CrystEngComm* 16 (26): 5793. https://doi.org/10.1039/c3ce42182a.

Bhattacharya, Biswajit, Susobhan Das, Garima Lal, Saundray Raj Soni, Animesh Ghosh, C. Malla Reddy, and Soumyajit Ghosh. 2020. “Screening, Crystal Structures and Solubility Studies of a Series of Multidrug Salt Hydrates and Cocrystals of Fenamic Acids with Trimethoprim and Sulfamethazine.” *Journal of Molecular Structure* 1199 (January): 127028. https://doi.org/10.1016/j.molstruc.2019.127028.

Bhattacharya, Biswajit, Amit Mondal, Saundray Raj Soni, Susobhan Das, Surojit Bhunia, K Bal Raju, Animesh Ghosh, and C Malla Reddy. 2018. “Multidrug Salt Forms of Norfloxacin with Non-Steroidal Anti-Inflammatory Drugs: Solubility and Membrane Permeability Studies.” *CrystEngComm* 20 (41): 6420–29. https://doi.org/10.1039/C8CE00900G.

Bouanga Boudiombo, Jacky S., and Ayesha Jacobs. 2018. “Salts of Mefenamic Acid With Amines: Structure, Thermal Stability, Desolvation, and Solubility.” *Journal of Pharmaceutical Sciences* 107 (12): 3014–21. https://doi.org/10.1016/j.xphs.2018.08.003.

Bouanga Boudiombo, Jacky S, and Ayesha Jacobs. 2016. “Solvates of Selected Fenamic Acids with Substituted Pyridines: Structure, Thermal Stability and Desolvation.” *Acta Crystallographica Section B Structural Science, Crystal Engineering and Materials* 72 (6): 836–45. https://doi.org/10.1107/S2052520616014128.

Childs, Scott L., G. Patrick Stahly, and Aeri Park. 2007. “The Salt−Cocrystal Continuum: The Influence of Crystal Structure on Ionization State.” *Molecular Pharmaceutics* 4 (3): 323–38. https://doi.org/10.1021/mp0601345.

Dhanaraj, V., and M. Vijayan. 1983. “A Hydrated 1:1 Complex between Niflumic Acid and Ethanolamine, C13H8F3N2O2−.C2H8NO+.H2O.” *Acta Crystallographica Section C Crystal Structure Communications* 39 (10): 1398–1401. https://doi.org/10.1107/S0108270183008653.

Dhanaraj, Venugopal, and Mamannamana Vijayan. 1987. “Crystal Structures of 1 : 1 Complexes of Meclofenamic Acid with Choline and Ethanolamine.” *Biochimica et Biophysica Acta* 924: 135–46.

Fábián, László, Noel Hamill, Kevin S. Eccles, Humphrey A. Moynihan, Anita R. Maguire, Linda McCausland, and Simon E. Lawrence. 2011. “Cocrystals of Fenamic Acids with Nicotinamide.” *Crystal Growth and Design* 11 (8): 3522–28. https://doi.org/10.1021/cg200429j.

Farhadikoutenaei, Abbas, and Abdul Razak Ibrahim. 2016. “CSD Communication.” https://doi.org/10.5517/ccdc.csd.cc13592n.

Fonari, Marina S., Eduard V. Ganin, Anna V. Vologzhanina, Mikhail Yu. Antipin, and Victor Ch. Kravtsov. 2010. “Persistent CH···π Interactions in Mefenamic Acid Complexes with Cyclic and Acyclic Amines.” *Crystal Growth & Design* 10 (8): 3647–56. https://doi.org/10.1021/cg100518b.

Jerzykiewicz, Lucjan, Adam Sroka, and Irena Majerz. 2016. “The Crystal Structure and Behavior of Fenamic Acid-Acridine Complex Under High Pressure.” *Journal of Pharmaceutical Sciences* 105 (12): 3487–95. https://doi.org/10.1016/j.xphs.2016.08.029.

Kumar, Vineet, Ram Thaimattam, Sanjay Dutta, Parthapratim Munshi, and Arunachalam Ramanan. 2017. “Structural Landscape of Multicomponent Solids Based on Sulfa Drugs.” *CrystEngComm* 19 (21): 2914–24. https://doi.org/10.1039/C7CE00217C.

Lemmerer, Andreas, Susan A. Bourne, Mino R. Caira, Jonathan Cotton, Umraan Hendricks, Laura C. Peinke, and Lee Trollope. 2010. “Incorporating Active Pharmaceutical Ingredients into a Molecular Salt Using a Chiral Counterion.” *CrystEngComm* 12 (11): 3634. https://doi.org/10.1039/c0ce00043d.

Mittapalli, Sudhir, M. K. Chaitanya Mannava, Rasmita Sahoo, and Ashwini Nangia. 2019. “Cocrystals, Salts, and Supramolecular Gels of Nonsteroidal Anti-Inflammatory Drug Niflumic Acid.” *Crystal Growth & Design* 19 (1): 219–30. https://doi.org/10.1021/acs.cgd.8b01298.

Nechipadappu, Sunil Kumar, Venkatadri Tekuri, and Darshak R. Trivedi. 2017. “Pharmaceutical Co-Crystal of Flufenamic Acid: Synthesis and Characterization of Two Novel Drug-Drug Co-Crystal.” *Journal of Pharmaceutical Sciences* 106 (5): 1384–90. https://doi.org/10.1016/j.xphs.2017.01.033.

Nechipadappu, Sunil Kumar, and Darshak R Trivedi. 2017. “Structural and Physicochemical Characterization of Pyridine Derivative Salts of Anti-Inflammatory Drugs.” *Journal of Molecular Structure* 1141 (August): 64–74. https://doi.org/10.1016/j.molstruc.2017.03.086.

Parveen, Rumana, Bommagani Sravanthi, and Parthasarathi Dastidar. 2017. “Rationally Developed Organic Salts of Tolfenamic Acid and Its β-Alanine Derivatives for Dual Purposes as an Anti-Inflammatory Topical Gel and Anticancer Agent.” *Chemistry – An Asian Journal* 12 (7): 792–803. https://doi.org/https://doi.org/10.1002/asia.201700049.

Pop, Mihaela M, Kees Goubitz, Gheorghe Borodi, Mircea Bogdan, Dirk J A De Ridder, Rene Peschar, and Henk Schenk. 2002. “Crystal Structure of the Inclusion Complex of {$β$}-Cyclodextrin with Mefenamic Acid from High-Resolution Synchrotron Powder-Diffraction Data in Combination with Molecular-Mechanics Calculations.” *Acta Crystallographica Section B* 58 (6): 1036–43. https://doi.org/10.1107/S010876810201947X.

Ranjan, Subham, Ramesh Devarapalli, Sudeshna Kundu, Subhankar Saha, Shubham Deolka, Venu R. Vangala, and C. Malla Reddy. 2020. “Isomorphism: `molecular Similarity to Crystal Structure Similarity’ in Multicomponent Forms of Analgesic Drugs Tolfenamic and Mefenamic Acid.” *IUCrJ* 7 (2): 173–83. https://doi.org/10.1107/S205225251901604X.

Roy, Rajdip, and Parthasarathi Dastidar. 2017. “Supramolecular Synthon Approach in Developing Anti-Inflammatory Topical Gels for In Vivo Self-Delivery.” *Chemistry - A European Journal* 23 (62): 15623–27. https://doi.org/10.1002/chem.201703850.

Roy, Rajdip, Jolly Deb, Siddhartha Sankar Jana, and Parthasarathi Dastidar. 2014. “Exploiting Supramolecular Synthons in Designing Gelators Derived from Multiple Drugs.” *Chemistry - A European Journal* 20 (47): 15320–24. https://doi.org/10.1002/chem.201404965.

Sangeetha, Ramalingam, Kasthuri Balasubramani, Kaliyaperumal Thanigaimani, and Ibrahim Abdul Razak. 2017. “6-Amino-3,4-Dimethyl-1,2,4-Triazin-1-Ium 2-Anilinobenzoate–3-Amino-5,6-Dimethyl-1,2,4-Triazine (1/1).” *IUCrData* 2 (6). https://doi.org/10.1107/s241431461700829x.

Sanphui, Palash, Geetha Bolla, and Ashwini Nangia. 2012. “High Solubility Piperazine Salts of the Nonsteroidal Anti-Inflammatory Drug (NSAID) Meclofenamic Acid.” *Crystal Growth & Design* 12 (4): 2023–36. https://doi.org/10.1021/cg300002p.

SeethaLekshmi, Sunil, and Tayur N. Guru Row. 2012. “Conformational Polymorphism in a Non-Steroidal Anti-Inflammatory Drug, Mefenamic Acid.” *Crystal Growth & Design* 12 (8): 4283–89. https://doi.org/10.1021/cg300812v.

Surov, Artem O., Alexander P. Voronin, Mikhail V. Vener, Andrei V. Churakov, and German L. Perlovich. 2018. “Specific Features of Supramolecular Organisation and Hydrogen Bonding in Proline Cocrystals: A Case Study of Fenamates and Diclofenac.” *CrystEngComm* 20 (43): 6970–81. https://doi.org/10.1039/C8CE01458B.

Surov, Artem O, Anna A Simagina, Nikolay G Manin, Lyudmila G Kuzmina, Andrei V Churakov, and German L Perlovich. 2015. “Fenamate Cocrystals with 4,4′-Bipyridine: Structural and Thermodynamic Aspects.” *Crystal Growth & Design* 15 (1): 228–38. https://doi.org/10.1021/cg5012633.

Toffoli, P., M. Coquillay, N. Rodier, R. Ceolin, J. M. Teulon, and C. Guechot. 1988. “Niflumate de Morpholinoéthyle (DCI: Morniflumate).” *Acta Crystallographica Section C Crystal Structure Communications* 44 (3): 547–50. https://doi.org/10.1107/S0108270187011156.

Vaksler, Ye.A., D Benedis, A.A. Dyshin, R.D. Oparin, N.T. Correia, F Capet, S.V. Shishkina, M.G. Kiselev, and A Idrissi. 2021. “Spectroscopic Characterization of Single Co-Crystal of Mefenamic Acid and Nicotinamide Using Supercritical CO2.” *Journal of Molecular Liquids* 334 (July): 116117. https://doi.org/10.1016/j.molliq.2021.116117.

Wang, Xinyi, Shijie Xu, Lina Jia, Yujia Yang, Yu Liu, Junbo Gong, and Songgu Wu. 2019. “Drug–Drug Salts of Mefenamic Acid\tolfenamic Acid and Piperazine to Improve Physicochemical Properties for Potential Veterinary Use.” *CrystEngComm* 21 (35): 5284–91. https://doi.org/10.1039/C9CE00781D.

Wittering, K. E., L. R. Agnew, A. R. Klapwijk, K. Robertson, A. J. P. Cousen, D. L. Cruickshank, and C. C. Wilson. 2015. “Crystallisation and Physicochemical Property Characterisation of Conformationally-Locked Co-Crystals of Fenamic Acid Derivatives.” *CrystEngComm* 17 (19): 3610–18. https://doi.org/10.1039/C5CE00297D.

Zheng, Qixuan, Samantha L Rood, Daniel K Unruh, and Kristin M Hutchins. 2018. “Co-Crystallization of Anti-Inflammatory Pharmaceutical Contaminants and Rare Carboxylic Acid–Pyridine Supramolecular Synthon Breakdown.” *CrystEngComm* 20 (41): 6377–81. https://doi.org/10.1039/C8CE01492B.
