# Supplementary material for: Multicomponent Solids of Niflumic and Mefenamic Acids Based on Acid-Pyridine Synthon
Source: Front Chem. 2022 Mar 31;10:729608. doi: 10.3389/fchem.2022.729608 (PMC9009247; doi:10.3389/fchem.2022.729608)
Supplement: Supplementary file 2 [file DataSheet1.PDF]

checkCIF/PLATON report

Structure factors have been supplied for datablock(s) ddmlm, glianm, glisnm, jdm19m, jdm71zm, m3a6_0m_a_a, mf2mpm, mf3mpm, mficm, mfsppm, mi6m, mi9m, nf2p_0m_a, nf3pdms_0m_a

THIS REPORT IS FOR GUIDANCE ONLY. IF USED AS PART OF A REVIEW PROCEDURE FOR PUBLICATION, IT SHOULD NOT REPLACE THE EXPERTISE OF AN EXPERIENCED CRYSTALLOGRAPHIC REFEREE.

No syntax errors found. CIF dictionary Interpreting this report

Datablock: jdm71zm

```
Bond precision:  C-C = 0.0043 A          Wavelength=0.71073

Cell:            a=10.6728(16)     b=12.4719(19)      c=25.232(4)
                 alpha=90          beta=92.169(3)     gamma=90
Temperature:     298 K
```

	Calculated	Reported
Volume	3356.2(9)	3356.3(9)
Space group	P 21/n	P 1 21/n 1
Hall group	-P 2yn	-P 2yn
Moiety formula	2(C13 H9 F3 N2 O2), C12 H12 N2	2(C13 H9 F3 N2 O2), C12 H12 N2
Sum formula	C38 H30 F6 N6 O4	C38 H30 F6 N6 O4
Mr	748.68	748.68
Dx,g cm-3	1.482	1.482
Z	4	4
Mu (mm-1)	0.121	0.121
F000	1544.0	1544.0
F000’	1544.94	
h,k,lmax	12,14,29	12,14,29
Nref	5916	5903
Tmin,Tmax	0.976,0.983	0.840,0.988
Tmin’	0.976	

```
Correction method= # Reported T Limits: Tmin=0.840 Tmax=0.988
AbsCorr = MULTI-SCAN

Data completeness= 0.998          Theta(max)= 24.997

R(reflections)= 0.0684( 4277)     wR2(reflections)= 0.1524( 5903)

S = 1.073                  Npar= 497
```

The following ALERTS were generated. Each ALERT has the format
test-name_ALERT_alert-type_alert-level.
Click on the hyperlinks for more details of the test.

Alert level C

```
PLAT241_ALERT_2_C High   'MainMol' Ueq as Compared to Neighbors of        C38 Check
PLAT340_ALERT_3_C Low Bond Precision on  C-C Bonds ...............    0.00429 Ang.
PLAT480_ALERT_4_C Long H...A H-Bond Reported H11      ..F1       .       2.59 Ang.
PLAT906_ALERT_3_C Large K Value in the Analysis of Variance ......     16.685 Check
PLAT906_ALERT_3_C Large K Value in the Analysis of Variance ......      3.083 Check
PLAT911_ALERT_3_C Missing FCF Refl Between Thmin & STh/L=    0.595         14 Report
```

Alert level G

```
PLAT007_ALERT_5_G Number of Unrefined Donor-H Atoms ..............          2 Report
PLAT242_ALERT_2_G Low    'MainMol' Ueq as Compared to Neighbors of        C26 Check
PLAT242_ALERT_2_G Low    'MainMol' Ueq as Compared to Neighbors of        C13 Check
PLAT432_ALERT_2_G Short Inter X...Y Contact  C9       ..C23              3.17 Ang.
                                          3/2-x,1/2+y,1/2-z  =      2_655 Check
PLAT883_ALERT_1_G No Info/Value for _atom_sites_solution_primary .     Please Do !
PLAT909_ALERT_3_G Percentage of I>2sig(I) Data at Theta(Max) Still        47% Note
PLAT941_ALERT_3_G Average HKL Measurement Multiplicity ...........        2.8 Low
PLAT978_ALERT_2_G Number C-C Bonds with Positive Residual Density.          4 Info
```

---

0 **ALERT level A** = Most likely a serious problem - resolve or explain
0 **ALERT level B** = A potentially serious problem, consider carefully
6 **ALERT level C** = Check. Ensure it is not caused by an omission or oversight
8 **ALERT level G** = General information/check it is not something unexpected

1 ALERT type 1 CIF construction/syntax error, inconsistent or missing data
5 ALERT type 2 Indicator that the structure model may be wrong or deficient
6 ALERT type 3 Indicator that the structure quality may be low
1 ALERT type 4 Improvement, methodology, query or suggestion
1 ALERT type 5 Informative message, check

Datablock: glisnm

Bond precision:	C-C = 0.0150 A		Wavelength=0.71073
Cell:	a=25.380(6)	b=12.495(3)	c=10.764(3)
	alpha=90	beta=92.040(4)	gamma=90
Temperature:	298 K		

	Calculated	Reported
Volume	3411.4(15)	3411.4(13)
Space group	C c	C 1 c 1
Hall group	C -2yc	C -2yc
Moiety formula	2(C13 H9 F3 N2 O2), C12 H10 N2	2(C13 H9 F3 N2 O2), C12 H10 N2
Sum formula	C38 H28 F6 N6 O4	C38 H28 F6 N6 O4
Mr	746.66	746.66
Dx,g cm-3	1.454	1.454
Z	4	4
Mu (mm-1)	0.119	0.119
F000	1536.0	1536.0
F000’	1536.94	
h,k,lmax	28,13,11	28,13,11
Nref	4894[2450]	3591
Tmin,Tmax	0.961,0.973	0.817,0.983
Tmin’	0.960	

Correction method= # Reported T Limits: Tmin=0.817 Tmax=0.983
AbsCorr = MULTI-SCAN

Data completeness= 1.47/0.73 Theta(max)= 23.271

R(reflections)= 0.0550(2337) wR2(reflections)= 0.1461(3591)

S = 1.025 Npar= 489

The following ALERTS were generated. Each ALERT has the format
test-name_ALERT_alert-type_alert-level.
Click on the hyperlinks for more details of the test.

Alert level B

```
THETM01_ALERT_3_B  The value of sine(theta_max)/wavelength is less than 0.575
            Calculated sin(theta_max)/wavelength =    0.5559
PLAT023_ALERT_3_B Resolution (too) Low [sin(theta)/Lambda < 0.6]..       0.56 Ang-1
PLAT089_ALERT_3_B Poor Data / Parameter Ratio (Zmax < 18) ........       4.99 Note
PLAT213_ALERT_2_B Atom F2              has ADP max/min Ratio .....        4.6 prolat
PLAT213_ALERT_2_B Atom F4              has ADP max/min Ratio .....        4.6 prolat
PLAT230_ALERT_2_B Hirshfeld Test Diff for    O4       --C39      .        7.1 s.u.
PLAT340_ALERT_3_B Low Bond Precision on  C-C Bonds ...............      0.015 Ang.
```

Alert level C

```
STRVA01_ALERT_4_C          Flack parameter is too small
           From the CIF: _refine_ls_abs_structure_Flack   -0.600
           From the CIF: _refine_ls_abs_structure_Flack_su    1.000
PLAT213_ALERT_2_C Atom C36             has ADP max/min Ratio .....        3.7 prolat
PLAT213_ALERT_2_C Atom C41             has ADP max/min Ratio .....        3.2 prolat
PLAT230_ALERT_2_C Hirshfeld Test Diff for    N28      --C31      .        5.5 s.u.
PLAT230_ALERT_2_C Hirshfeld Test Diff for    O1       --C2       .        6.1 s.u.
PLAT234_ALERT_4_C Large Hirshfeld Difference F1       --C36      .       0.18 Ang.
PLAT234_ALERT_4_C Large Hirshfeld Difference F3       --C36      .       0.18 Ang.
```

```
PLAT234_ALERT_4_C Large Hirshfeld Difference C32      --C33      .       0.18 Ang.
PLAT234_ALERT_4_C Large Hirshfeld Difference N6       --C13      .       0.17 Ang.
PLAT234_ALERT_4_C Large Hirshfeld Difference C15      --C22      .       0.18 Ang.
PLAT234_ALERT_4_C Large Hirshfeld Difference C15      --C41      .       0.20 Ang.
PLAT234_ALERT_4_C Large Hirshfeld Difference N2       --C14      .       0.17 Ang.
PLAT234_ALERT_4_C Large Hirshfeld Difference C4       --C25      .       0.21 Ang.
PLAT234_ALERT_4_C Large Hirshfeld Difference C7       --C9       .       0.17 Ang.
PLAT234_ALERT_4_C Large Hirshfeld Difference C7       --C24      .       0.18 Ang.
PLAT234_ALERT_4_C Large Hirshfeld Difference C19      --C25      .       0.19 Ang.
PLAT241_ALERT_2_C High   'MainMol' Ueq as Compared to Neighbors of         C8 Check
PLAT241_ALERT_2_C High   'MainMol' Ueq as Compared to Neighbors of        C25 Check
PLAT242_ALERT_2_C Low    'MainMol' Ueq as Compared to Neighbors of         C2 Check
PLAT242_ALERT_2_C Low    'MainMol' Ueq as Compared to Neighbors of        C15 Check
PLAT250_ALERT_2_C Large U3/U1 Ratio for Average U(i,j) Tensor ....        2.2 Note
PLAT250_ALERT_2_C Large U3/U1 Ratio for Average U(i,j) Tensor ....        2.8 Note
PLAT250_ALERT_2_C Large U3/U1 Ratio for Average U(i,j) Tensor ....        2.2 Note
PLAT334_ALERT_2_C Small Aver. Benzene C-C Dist C12      -C18             1.37 Ang.
PLAT790_ALERT_4_C Centre of Gravity not Within Unit Cell: Resd.  #          1 Note
              C13 H9 F3 N2 O2
PLAT906_ALERT_3_C Large K Value in the Analysis of Variance ......      2.100 Check
PLAT911_ALERT_3_C Missing FCF Refl Between Thmin & STh/L=    0.556          9 Report
```

Alert level G

```
PLAT007_ALERT_5_G Number of Unrefined Donor-H Atoms ..............          4 Report
PLAT032_ALERT_4_G Std. Uncertainty on Flack Parameter Value High .      1.000 Report
PLAT115_ALERT_5_G ADDSYM Detects Noncrystallographic Inversion ...        92% Check
PLAT152_ALERT_1_G The Supplied and Calc. Volume s.u. Differ by ...          2 Units
PLAT242_ALERT_2_G Low    'MainMol' Ueq as Compared to Neighbors of        C36 Check
PLAT242_ALERT_2_G Low    'MainMol' Ueq as Compared to Neighbors of        C41 Check
PLAT432_ALERT_2_G Short Inter X...Y Contact  C16      ..C34              3.19 Ang.
                                                    x,y,z  =      1_555 Check
PLAT790_ALERT_4_G Centre of Gravity not Within Unit Cell: Resd.  #          2 Note
              C13 H9 F3 N2 O2
PLAT790_ALERT_4_G Centre of Gravity not Within Unit Cell: Resd.  #          3 Note
              C12 H10 N2
PLAT883_ALERT_1_G No Info/Value for _atom_sites_solution_primary .     Please Do !
PLAT909_ALERT_3_G Percentage of I>2sig(I) Data at Theta(Max) Still        34% Note
PLAT910_ALERT_3_G Missing # of FCF Reflection(s) Below Theta(Min).          3 Note
PLAT915_ALERT_3_G No Flack x Check Done: Low Friedel Pair Coverage         47 %
PLAT916_ALERT_2_G Hooft y and Flack x Parameter Values Differ by .       1.20 Check
PLAT933_ALERT_2_G Number of OMIT Records in Embedded .res File ...          2 Note
PLAT941_ALERT_3_G Average HKL Measurement Multiplicity ...........        2.5 Low
PLAT978_ALERT_2_G Number C-C Bonds with Positive Residual Density.          0 Info
PLAT992_ALERT_5_G Repd & Actual _reflns_number_gt Values Differ by          2 Check
```

---

 0 **ALERT level A** = Most likely a serious problem - resolve or explain
 7 **ALERT level B** = A potentially serious problem, consider carefully
27 **ALERT level C** = Check. Ensure it is not caused by an omission or oversight
18 **ALERT level G** = General information/check it is not something unexpected

```
 2 ALERT type 1 CIF construction/syntax error, inconsistent or missing data
21 ALERT type 2 Indicator that the structure model may be wrong or deficient
10 ALERT type 3 Indicator that the structure quality may be low
16 ALERT type 4 Improvement, methodology, query or suggestion
 3 ALERT type 5 Informative message, check
```

Datablock: jdm19m

Bond precision: C-C = 0.0091 A Wavelength=0.71073

Cell:	a=9.355(5)	b=10.722(6)	c=11.110(6)
	alpha=67.844(11)	beta=79.027(12)	gamma=75.084(11)
Temperature:	273 K		

	Calculated	Reported
Volume	991.9(9)	991.9(9)
Space group	P -1	P -1
Hall group	-P 1	-P 1
Moiety formula	C13 H9 F3 N2 O2, 0.5(C12 H10 N2), 0.5(C4 H8 O2)	C13 H9 F3 N2 O2, 0.5(C12 H10 N2), 0.5(C4 H8 O2)
Sum formula	C21 H18 F3 N3 O3	C21 H18 F3 N3 O3
Mr	417.38	417.38
Dx,g cm-3	1.398	1.398
Z	2	2
Mu (mm-1)	0.114	0.114
F000	432.0	432.0
F000’	432.27	
h,k,lmax	11,12,13	11,12,13
Nref	3498	3492
Tmin,Tmax	0.968,0.986	
Tmin’	0.966	

Correction method= Not given

Data completeness= 0.998 Theta(max)= 24.988

R(reflections)= 0.0902(1358) wR2(reflections)= 0.2861(3492)

S = 0.998 Npar= 276

The following ALERTS were generated. Each ALERT has the format
test-name_ALERT_alert-type_alert-level.
Click on the hyperlinks for more details of the test.

Alert level B

```
PLAT026_ALERT_3_B Ratio Observed / Unique Reflections (too) Low ..        39% Check
PLAT360_ALERT_2_B Short  C(sp3)-C(sp3) Bond  C19      - C27_a    .       1.30 Ang.
PLAT410_ALERT_2_B Short Intra H...H Contact  H19A     ..H27B     .       1.86 Ang.
                                                 -x,2-y,1-z  =      2_576 Check
PLAT410_ALERT_2_B Short Intra H...H Contact  H19B     ..H27A     .       1.85 Ang.
                                                 -x,2-y,1-z  =      2_576 Check
```

Alert level C

```
PLAT084_ALERT_3_C High wR2 Value (i.e. > 0.25) ...................       0.29 Report
PLAT234_ALERT_4_C Large Hirshfeld Difference F5       --C12      .       0.18 Ang.
PLAT242_ALERT_2_C Low    'MainMol' Ueq as Compared to Neighbors of        C11 Check
PLAT244_ALERT_4_C Low    'Solvent' Ueq as Compared to Neighbors of         O6 Check
```

```
PLAT245_ALERT_2_C U(iso) H2        Smaller than U(eq) N2        by      0.026 Ang**2
PLAT260_ALERT_2_C Large Average Ueq of Residue Including        O6      0.184 Check
PLAT340_ALERT_3_C Low Bond Precision on  C-C Bonds ...............    0.00905 Ang.
PLAT906_ALERT_3_C Large K Value in the Analysis of Variance ......     46.832 Check
PLAT906_ALERT_3_C Large K Value in the Analysis of Variance ......      2.046 Check
PLAT906_ALERT_3_C Large K Value in the Analysis of Variance ......      8.107 Check
PLAT906_ALERT_3_C Large K Value in the Analysis of Variance ......      3.039 Check
PLAT911_ALERT_3_C Missing FCF Refl Between Thmin & STh/L=    0.594          6 Report
```

Alert level G

```
PLAT007_ALERT_5_G Number of Unrefined Donor-H Atoms ..............          1 Report
PLAT072_ALERT_2_G SHELXL First  Parameter in WGHT  Unusually Large       0.13 Report
PLAT199_ALERT_1_G Reported _cell_measurement_temperature ..... (K)        273 Check
PLAT200_ALERT_1_G Reported   _diffrn_ambient_temperature ..... (K)        273 Check
PLAT242_ALERT_2_G Low    'MainMol' Ueq as Compared to Neighbors of        C12 Check
PLAT344_ALERT_2_G Unusual sp3       Angle Range in Solvent/Ion for        C27 Check
PLAT883_ALERT_1_G No Info/Value for _atom_sites_solution_primary .     Please Do !
PLAT941_ALERT_3_G Average HKL Measurement Multiplicity ...........        2.8 Low
PLAT978_ALERT_2_G Number C-C Bonds with Positive Residual Density.          0 Info
```

---

0 **ALERT level A** = Most likely a serious problem - resolve or explain
4 **ALERT level B** = A potentially serious problem, consider carefully
12 **ALERT level C** = Check. Ensure it is not caused by an omission or oversight
9 **ALERT level G** = General information/check it is not something unexpected

3 ALERT type 1 CIF construction/syntax error, inconsistent or missing data
10 ALERT type 2 Indicator that the structure model may be wrong or deficient
9 ALERT type 3 Indicator that the structure quality may be low
2 ALERT type 4 Improvement, methodology, query or suggestion
1 ALERT type 5 Informative message, check

Datablock: glianm

Bond precision: C-C = 0.0073 A   Wavelength=0.71073

Cell:	a=17.889(4)	b=7.4942(18)	c=28.063(6)
	alpha=90	beta=104.669(5)	gamma=90
Temperature:	273 K		

	Calculated	Reported
Volume	3639.6(14)	3639.6(14)
Space group	C 2/c	C 1 2/c 1
Hall group	-C 2yc	-C 2yc
Moiety formula	2(C13 H9 F3 N2 O2), C13 H14 N2	2(C13 H9 F3 N2 O2), C13 H14 N2
Sum formula	C39 H32 F6 N6 O4	C39 H32 F6 N6 O4
Mr	762.71	762.71
Dx,g cm-3	1.392	1.392
Z	4	4
Mu (mm-1)	0.113	0.113
F000	1576.0	1576.0
F000’	1576.95	
h,k,lmax	19,8,31	19,8,31
Nref	2615	2566
Tmin,Tmax	0.976,0.983	
Tmin’	0.973	

Correction method= Not given

Data completeness= 0.981 Theta(max)= 23.260

R(reflections)= 0.0848(1731) wR2(reflections)= 0.2339(2566)

S = 1.084 Npar= 250

The following ALERTS were generated. Each ALERT has the format
test-name_ALERT_alert-type_alert-level.
Click on the hyperlinks for more details of the test.

Alert level B

```
THETM01_ALERT_3_B  The value of sine(theta_max)/wavelength is less than 0.575
            Calculated sin(theta_max)/wavelength =    0.5556
PLAT023_ALERT_3_B Resolution (too) Low [sin(theta)/Lambda < 0.6]..       0.56 Ang-1
```

Alert level C

```
PLAT230_ALERT_2_C Hirshfeld Test Diff for    C1       --C6       .        5.7 s.u.
PLAT340_ALERT_3_C Low Bond Precision on  C-C Bonds ...............    0.00728 Ang.
PLAT480_ALERT_4_C Long H...A H-Bond Reported H14      ..F3       .       2.59 Ang.
PLAT906_ALERT_3_C Large K Value in the Analysis of Variance ......     29.728 Check
PLAT906_ALERT_3_C Large K Value in the Analysis of Variance ......      5.247 Check
PLAT906_ALERT_3_C Large K Value in the Analysis of Variance ......      2.317 Check
PLAT911_ALERT_3_C Missing FCF Refl Between Thmin & STh/L=    0.556         46 Report
```

Alert level G

```
PLAT007_ALERT_5_G Number of Unrefined Donor-H Atoms ..............          2 Report
PLAT072_ALERT_2_G SHELXL First  Parameter in WGHT  Unusually Large       0.11 Report
PLAT199_ALERT_1_G Reported _cell_measurement_temperature ..... (K)        273 Check
PLAT200_ALERT_1_G Reported   _diffrn_ambient_temperature ..... (K)        273 Check
PLAT242_ALERT_2_G Low    'MainMol' Ueq as Compared to Neighbors of        C21 Check
```

```
PLAT300_ALERT_4_G Atom Site Occupancy of H18A       Constrained at        0.5 Check
PLAT300_ALERT_4_G Atom Site Occupancy of H18B       Constrained at        0.5 Check
PLAT367_ALERT_2_G Long?  C(sp?)-C(sp?) Bond  C11      - C18      .       1.52 Ang.
PLAT883_ALERT_1_G No Info/Value for _atom_sites_solution_primary .     Please Do !
PLAT910_ALERT_3_G Missing # of FCF Reflection(s) Below Theta(Min).          4 Note
PLAT933_ALERT_2_G Number of OMIT Records in Embedded .res File ...          2 Note
PLAT941_ALERT_3_G Average HKL Measurement Multiplicity ...........        2.5 Low
PLAT955_ALERT_1_G Reported (CIF) and Actual (FCF) Lmax Differ by .          1 Units
PLAT978_ALERT_2_G Number C-C Bonds with Positive Residual Density.          1 Info
```

```
   0 ALERT level A = Most likely a serious problem - resolve or explain
   2 ALERT level B = A potentially serious problem, consider carefully
   7 ALERT level C = Check. Ensure it is not caused by an omission or oversight
  14 ALERT level G = General information/check it is not something unexpected

   4 ALERT type 1 CIF construction/syntax error, inconsistent or missing data
   6 ALERT type 2 Indicator that the structure model may be wrong or deficient
   9 ALERT type 3 Indicator that the structure quality may be low
   3 ALERT type 4 Improvement, methodology, query or suggestion
   1 ALERT type 5 Informative message, check
```

Datablock: mf3mpm

Bond precision: C-C = 0.0079 A Wavelength=0.71073

Cell:	a=7.413(2)	b=33.061(10)	c=7.690(2)
	alpha=90	beta=109.745(6)	gamma=90
Temperature:	273 K		

	Calculated	Reported
Volume	1773.9(9)	1773.8(9)
Space group	C c	C 1 c 1
Hall group	C -2yc	C -2yc
Moiety formula	C13 H8 F3 N2 O2, C5 H7 N2	C13 H8 F3 N2 O2, C5 H7 N2
Sum formula	C18 H15 F3 N4 O2	C18 H15 F3 N4 O2
Mr	376.34	376.34
Dx,g cm-3	1.409	1.409
Z	4	4
Mu (mm-1)	0.116	0.116
F000	776.0	776.0
F000'	776.46	
h,k,lmax	8,36,8	8,36,8
Nref	2568[1293]	1637
Tmin,Tmax	0.971,0.984	
Tmin'	0.964	

Correction method= Not given

Data completeness= 1.27/0.64 Theta(max)= 23.289

```
R(reflections)= 0.0427( 1522)     wR2(reflections)= 0.1068( 1637)

S = 1.034                     Npar= 244
```

```
The following ALERTS were generated. Each ALERT has the format
       test-name_ALERT_alert-type_alert-level.
Click on the hyperlinks for more details of the test.
```

Alert level B

```
THETM01_ALERT_3_B  The value of sine(theta_max)/wavelength is less than 0.575
            Calculated sin(theta_max)/wavelength =    0.5563
PLAT023_ALERT_3_B Resolution (too) Low [sin(theta)/Lambda < 0.6]..       0.56 Ang-1
PLAT089_ALERT_3_B Poor Data / Parameter Ratio (Zmax < 18) ........       5.23 Note
```

Alert level C

```
STRVA01_ALERT_4_C           Flack test results are meaningless.
           From the CIF: _refine_ls_abs_structure_Flack   -0.200
           From the CIF: _refine_ls_abs_structure_Flack_su    1.000
PLAT234_ALERT_4_C Large Hirshfeld Difference F3       --C13      .       0.16 Ang.
PLAT334_ALERT_2_C Small Aver. Benzene C-C Dist C3       -C11             1.37 Ang.
PLAT340_ALERT_3_C Low Bond Precision on  C-C Bonds ...............    0.00788 Ang.
PLAT911_ALERT_3_C Missing FCF Refl Between Thmin & STh/L=    0.556         15 Report
```

Alert level G

```
PLAT007_ALERT_5_G Number of Unrefined Donor-H Atoms ..............          4 Report
PLAT032_ALERT_4_G Std. Uncertainty on Flack Parameter Value High .      1.000 Report
PLAT199_ALERT_1_G Reported _cell_measurement_temperature ..... (K)        273 Check
PLAT200_ALERT_1_G Reported   _diffrn_ambient_temperature ..... (K)        273 Check
PLAT242_ALERT_2_G Low    'MainMol' Ueq as Compared to Neighbors of        C13 Check
PLAT883_ALERT_1_G No Info/Value for _atom_sites_solution_primary .     Please Do !
PLAT909_ALERT_3_G Percentage of I>2sig(I) Data at Theta(Max) Still        65% Note
PLAT910_ALERT_3_G Missing # of FCF Reflection(s) Below Theta(Min).          3 Note
PLAT915_ALERT_3_G No Flack x Check Done: Low Friedel Pair Coverage         28 %
PLAT933_ALERT_2_G Number of OMIT Records in Embedded .res File ...          1 Note
PLAT941_ALERT_3_G Average HKL Measurement Multiplicity ...........        2.5 Low
PLAT978_ALERT_2_G Number C-C Bonds with Positive Residual Density.          0 Info
```

```
   0 ALERT level A = Most likely a serious problem - resolve or explain
   3 ALERT level B = A potentially serious problem, consider carefully
   5 ALERT level C = Check. Ensure it is not caused by an omission or oversight
  12 ALERT level G = General information/check it is not something unexpected

   3 ALERT type 1 CIF construction/syntax error, inconsistent or missing data
   4 ALERT type 2 Indicator that the structure model may be wrong or deficient
   9 ALERT type 3 Indicator that the structure quality may be low
   3 ALERT type 4 Improvement, methodology, query or suggestion
   1 ALERT type 5 Informative message, check
```

Datablock: nf2p_0m_a

```
Bond precision:   C-C = 0.0021 A          Wavelength=0.71073
```

Cell:	a=8.0525(5)	b=8.1125(5)	c=25.6798(18)
	alpha=87.750(2)	beta=83.626(2)	gamma=89.763(2)
Temperature:	298 K		

	Calculated	Reported
Volume	1665.90(19)	1665.90(19)
Space group	P -1	P -1
Hall group	-P 1	-P 1
Moiety formula	C13 H8 F3 N2 O2, C5 H7 N2	C13 H8 F3 N2 O2, C5 H7 N2
Sum formula	C18 H15 F3 N4 O2	C18 H15 F3 N4 O2
Mr	376.34	376.34
Dx,g cm-3	1.500	1.500
Z	4	4
Mu (mm-1)	0.123	0.123
F000	776.0	776.0
F000’	776.47	
h,k,lmax	9,9,30	9,9,30
Nref	5898	5861
Tmin,Tmax	0.973,0.981	0.876,0.998
Tmin’	0.973	

Correction method= # Reported T Limits: Tmin=0.876 Tmax=0.998
AbsCorr = MULTI-SCAN

Data completeness= 0.994 Theta(max)= 24.998

R(reflections)= 0.0459(5204) wR2(reflections)= 0.1277(5861)

S = 1.057 Npar= 487

The following ALERTS were generated. Each ALERT has the format
test-name_ALERT_alert-type_alert-level.
Click on the hyperlinks for more details of the test.

Alert level C

PLAT242_ALERT_2_C	Low ’MainMol’ Ueq as Compared to Neighbors of	C10	Check
PLAT911_ALERT_3_C	Missing FCF Refl Between Thmin & STh/L= 0.595	37	Report
PLAT913_ALERT_3_C	Missing # of Very Strong Reflections in FCF	9	Note
PLAT918_ALERT_3_C	Reflection(s) with I(obs) much Smaller I(calc) .	1	Check

---

Alert level G

PLAT007_ALERT_5_G	Number of Unrefined Donor-H Atoms	8	Report
PLAT154_ALERT_1_G	The s.u.’s on the Cell Angles are Equal ..(Note)	0.002	Degree
PLAT720_ALERT_4_G	Number of Unusual/Non-Standard Labels	6	Note
PLAT883_ALERT_1_G	No Info/Value for _atom_sites_solution_primary .	Please	Do !
PLAT909_ALERT_3_G	Percentage of I>2sig(I) Data at Theta(Max) Still	76%	Note
PLAT910_ALERT_3_G	Missing # of FCF Reflection(s) Below Theta(Min).	2	Note
PLAT933_ALERT_2_G	Number of OMIT Records in Embedded .res File ...	1	Note
PLAT941_ALERT_3_G	Average HKL Measurement Multiplicity	2.9	Low
PLAT978_ALERT_2_G	Number C-C Bonds with Positive Residual Density.	7	Info

PLAT992_ALERT_5_G Repd & Actual _reflns_number_gt Values Differ by 2 Check

---

0 **ALERT level A** = Most likely a serious problem - resolve or explain
0 **ALERT level B** = A potentially serious problem, consider carefully
4 **ALERT level C** = Check. Ensure it is not caused by an omission or oversight
10 **ALERT level G** = General information/check it is not something unexpected

2 ALERT type 1 CIF construction/syntax error, inconsistent or missing data
3 ALERT type 2 Indicator that the structure model may be wrong or deficient
6 ALERT type 3 Indicator that the structure quality may be low
1 ALERT type 4 Improvement, methodology, query or suggestion
2 ALERT type 5 Informative message, check

Datablock: nf3pdms_0m_a

Bond precision: C-C = 0.0023 A Wavelength=0.71073

Cell:	a=7.8043(3)	b=12.2520(5)	c=34.9035(15)
	alpha=90	beta=90	gamma=90
Temperature:	100 K		

	Calculated	Reported
Volume	3337.4(2)	3337.4(2)
Space group	P b c a	P b c a
Hall group	-P 2ac 2ab	-P 2ac 2ab
Moiety formula	C13 H8 F3 N2 O2, C5 H7 N2	C13 H8 F3 N2 O2, C5 H7 N2
Sum formula	C18 H15 F3 N4 O2	C18 H15 F3 N4 O2
Mr	376.34	376.34
Dx,g cm-3	1.498	1.498
Z	8	8
Mu (mm-1)	0.123	0.123
F000	1552.0	1552.0
F000’	1552.93	
h,k,lmax	9,14,41	9,14,41
Nref	2944	2943
Tmin,Tmax	0.969,0.988	
Tmin’	0.961	

Correction method= Not given

Data completeness= 1.000 Theta(max)= 24.988

R(reflections)= 0.0408(2420) wR2(reflections)= 0.1328(2943)

S = 0.958 Npar= 252

```
The following ALERTS were generated. Each ALERT has the format
       test-name_ALERT_alert-type_alert-level.
Click on the hyperlinks for more details of the test.
```

Alert level B

```
RINTA01_ALERT_3_B  The value of Rint is greater than 0.18
            Rint given   0.194
```

Alert level C

```
PLAT353_ALERT_3_C Long   N-H (N0.87,N1.01A)  N1       - H1       .       1.07 Ang.
```

Alert level G

```
PLAT007_ALERT_5_G Number of Unrefined Donor-H Atoms ..............         2 Report
PLAT020_ALERT_3_G The Value of Rint is Greater Than 0.12 .........     0.194 Report
PLAT720_ALERT_4_G Number of Unusual/Non-Standard Labels ..........         2 Note
PLAT883_ALERT_1_G No Info/Value for _atom_sites_solution_primary .    Please Do !
PLAT909_ALERT_3_G Percentage of I>2sig(I) Data at Theta(Max) Still       60% Note
PLAT910_ALERT_3_G Missing # of FCF Reflection(s) Below Theta(Min).         1 Note
PLAT913_ALERT_3_G Missing # of Very Strong Reflections in FCF ....         1 Note
PLAT978_ALERT_2_G Number C-C Bonds with Positive Residual Density.         1 Info
```

```
   0 ALERT level A = Most likely a serious problem - resolve or explain
   1 ALERT level B = A potentially serious problem, consider carefully
   1 ALERT level C = Check. Ensure it is not caused by an omission or oversight
   8 ALERT level G = General information/check it is not something unexpected

   1 ALERT type 1 CIF construction/syntax error, inconsistent or missing data
   1 ALERT type 2 Indicator that the structure model may be wrong or deficient
   6 ALERT type 3 Indicator that the structure quality may be low
   1 ALERT type 4 Improvement, methodology, query or suggestion
   1 ALERT type 5 Informative message, check
```

Datablock: ddmlm

```
Bond precision:   C-C = 0.0042 A            Wavelength=0.71073

Cell:              a=14.6959(16)     b=10.4708(16)      c=22.935(3)
                   alpha=90          beta=95.197(3)     gamma=90
Temperature:       298 K
```

	Calculated	Reported
Volume	3514.7(8)	3514.8(8)
Space group	C 2/c	C 1 2/c 1
Hall group	-C 2yc	-C 2yc
Moiety formula	C13 H8 F3 N2 O2, C5 H7 N2	C13 H8 F3 N2 O2, C5 H7 N2
Sum formula	C18 H15 F3 N4 O2	C18 H15 F3 N4 O2
Mr	376.34	376.34
Dx,g cm-3	1.422	1.422
Z	8	8
Mu (mm-1)	0.117	0.117
F000	1552.0	1798.0
F000’	1552.93	
h,k,lmax	17,12,27	17,12,27
Nref	3102	3100
Tmin,Tmax	0.973,0.983	0.873,0.989
Tmin’	0.973	

Correction method= # Reported T Limits: Tmin=0.873 Tmax=0.989
AbsCorr = MULTI-SCAN

Data completeness= 0.999 Theta(max)= 24.997

R(reflections)= 0.0705(2637) wR2(reflections)= 0.1820(3100)

S = 1.074 Npar= 244

The following ALERTS were generated. Each ALERT has the format
test-name_ALERT_alert-type_alert-level.
Click on the hyperlinks for more details of the test.

Alert level C

```
PLAT068_ALERT_1_C Reported F000 Differs from Calcd (or Missing)...     Please Check
PLAT340_ALERT_3_C Low Bond Precision on  C-C Bonds ...............    0.00419 Ang.
PLAT480_ALERT_4_C Long H...A H-Bond Reported H5       ..F2       .       2.61 Ang.
PLAT906_ALERT_3_C Large K Value in the Analysis of Variance ......     12.585 Check
PLAT906_ALERT_3_C Large K Value in the Analysis of Variance ......      2.738 Check
PLAT911_ALERT_3_C Missing FCF Refl Between Thmin & STh/L=    0.595          3 Report
```

Alert level G

```
PLAT007_ALERT_5_G Number of Unrefined Donor-H Atoms ..............          4 Report
PLAT083_ALERT_2_G SHELXL Second Parameter in WGHT  Unusually Large       5.34 Why ?
PLAT242_ALERT_2_G Low    'MainMol' Ueq as Compared to Neighbors of        C13 Check
PLAT883_ALERT_1_G No Info/Value for _atom_sites_solution_primary .     Please Do !
PLAT909_ALERT_3_G Percentage of I>2sig(I) Data at Theta(Max) Still        58% Note
PLAT978_ALERT_2_G Number C-C Bonds with Positive Residual Density.          3 Info
```

---

0 **ALERT level A** = Most likely a serious problem - resolve or explain
0 **ALERT level B** = A potentially serious problem, consider carefully
6 **ALERT level C** = Check. Ensure it is not caused by an omission or oversight
6 **ALERT level G** = General information/check it is not something unexpected

2 ALERT type 1 CIF construction/syntax error, inconsistent or missing data
3 ALERT type 2 Indicator that the structure model may be wrong or deficient
5 ALERT type 3 Indicator that the structure quality may be low
1 ALERT type 4 Improvement, methodology, query or suggestion
1 ALERT type 5 Informative message, check

Datablock: mficm

Bond precision: C-C = 0.0045 A Wavelength=0.71073

Cell:	a=7.783(2)	b=8.866(3)	c=13.261(4)
	alpha=101.121(5)	beta=98.179(6)	gamma=92.780(5)
Temperature:	298 K		

	Calculated	Reported
Volume	886.0(5)	886.0(5)
Space group	P -1	P -1
Hall group	-P 1	-P 1
Moiety formula	C15 H15 N O2, 0.5(C12 H12 N2)	C15 H15 N O2, 0.5(C12 H12 N2)
Sum formula	C21 H21 N2 O2	C21 H21 N2 O2
Mr	333.40	333.40
Dx,g cm-3	1.250	1.250
Z	2	2
Mu (mm-1)	0.081	0.081
F000	354.0	354.0
F000’	354.15	
h,k,lmax	9,10,15	9,10,15
Nref	3122	3113
Tmin,Tmax	0.977,0.987	0.839,0.993
Tmin’	0.975	

Correction method= # Reported T Limits: Tmin=0.839 Tmax=0.993
AbsCorr = MULTI-SCAN

Data completeness= 0.997 Theta(max)= 24.993

R(reflections)= 0.0795(2437) wR2(reflections)= 0.1892(3113)

S = 1.148 Npar= 233

The following ALERTS were generated. Each ALERT has the format
test-name_ALERT_alert-type_alert-level.
Click on the hyperlinks for more details of the test.

Alert level C

```
PLAT250_ALERT_2_C Large U3/U1 Ratio for Average U(i,j) Tensor ....        2.1 Note
PLAT340_ALERT_3_C Low Bond Precision on  C-C Bonds ...............    0.00452 Ang.
PLAT906_ALERT_3_C Large K Value in the Analysis of Variance ......     24.279 Check
PLAT906_ALERT_3_C Large K Value in the Analysis of Variance ......      3.659 Check
PLAT906_ALERT_3_C Large K Value in the Analysis of Variance ......      2.193 Check
PLAT911_ALERT_3_C Missing FCF Refl Between Thmin & STh/L=    0.594         10 Report
```

Alert level G

```
PLAT007_ALERT_5_G Number of Unrefined Donor-H Atoms ..............          1 Report
PLAT883_ALERT_1_G No Info/Value for _atom_sites_solution_primary .     Please Do !
PLAT909_ALERT_3_G Percentage of I>2sig(I) Data at Theta(Max) Still        52% Note
PLAT910_ALERT_3_G Missing # of FCF Reflection(s) Below Theta(Min).          1 Note
PLAT941_ALERT_3_G Average HKL Measurement Multiplicity ...........        2.8 Low
PLAT978_ALERT_2_G Number C-C Bonds with Positive Residual Density.          0 Info
PLAT992_ALERT_5_G Repd & Actual _reflns_number_gt Values Differ by          5 Check
```

0 **ALERT level A** = Most likely a serious problem - resolve or explain
0 **ALERT level B** = A potentially serious problem, consider carefully
6 **ALERT level C** = Check. Ensure it is not caused by an omission or oversight
7 **ALERT level G** = General information/check it is not something unexpected

1 ALERT type 1 CIF construction/syntax error, inconsistent or missing data
2 ALERT type 2 Indicator that the structure model may be wrong or deficient
8 ALERT type 3 Indicator that the structure quality may be low
0 ALERT type 4 Improvement, methodology, query or suggestion
2 ALERT type 5 Informative message, check

Datablock: mi6m

```
Bond precision:  C-C = 0.0043 A         Wavelength=0.71073

Cell:          a=7.8110(18)      b=8.790(2)        c=13.110(3)
               alpha=99.990(4)   beta=98.699(4)    gamma=92.114(5)
Temperature:   273 K
```

	Calculated	Reported
Volume	874.4(3)	874.4(3)
Space group	P -1	P -1
Hall group	-P 1	-P 1
Moiety formula	C15 H15 N O2, 0.5(C12 H10 N2)	C15 H15 N O2, 0.5(C12 H10 N2)
Sum formula	C21 H20 N2 O2	C21 H20 N2 O2
Mr	332.39	332.39
Dx,g cm-3	1.263	1.263
Z	2	2
Mu (mm-1)	0.082	0.082
F000	352.0	352.0
F000'	352.15	
h,k,lmax	9,10,15	9,10,15
Nref	3085	3080
Tmin,Tmax	0.981,0.986	0.777,0.991
Tmin'	0.981	

Correction method= # Reported T Limits: Tmin=0.777 Tmax=0.991
AbsCorr = MULTI-SCAN

Data completeness= 0.998 Theta(max)= 24.998

R(reflections)= 0.0719(2101) wR2(reflections)= 0.1817(3020)

S = 1.034 Npar= 229

The following ALERTS were generated. Each ALERT has the format
test-name_ALERT_alert-type_alert-level.
Click on the hyperlinks for more details of the test.

Alert level C

```
PLAT029_ALERT_3_C _diffrn_measured_fraction_theta_full value Low .      0.979 Why?
PLAT230_ALERT_2_C Hirshfeld Test Diff for    C4       --C6       .        7.0 s.u.
PLAT230_ALERT_2_C Hirshfeld Test Diff for    C6       --C14      .        6.7 s.u.
PLAT340_ALERT_3_C Low Bond Precision on  C-C Bonds ...............    0.00433 Ang.
PLAT906_ALERT_3_C Large K Value in the Analysis of Variance ......     18.799 Check
PLAT906_ALERT_3_C Large K Value in the Analysis of Variance ......      3.563 Check
PLAT911_ALERT_3_C Missing FCF Refl Between Thmin & STh/L=    0.595         63 Report
```

Alert level G

```
PLAT007_ALERT_5_G Number of Unrefined Donor-H Atoms ..............          2 Report
PLAT180_ALERT_4_G Check Cell Rounding: # of Values Ending with 0 =          4 Note
PLAT199_ALERT_1_G Reported _cell_measurement_temperature ..... (K)        273 Check
PLAT200_ALERT_1_G Reported   _diffrn_ambient_temperature ..... (K)        273 Check
PLAT380_ALERT_4_G Incorrectly? Oriented X(sp2)-Methyl Moiety .....        C21 Check
PLAT883_ALERT_1_G No Info/Value for _atom_sites_solution_primary .     Please Do !
PLAT909_ALERT_3_G Percentage of I>2sig(I) Data at Theta(Max) Still        39% Note
PLAT910_ALERT_3_G Missing # of FCF Reflection(s) Below Theta(Min).          1 Note
PLAT933_ALERT_2_G Number of OMIT Records in Embedded .res File ...          1 Note
PLAT941_ALERT_3_G Average HKL Measurement Multiplicity ...........        1.5 Low
```

PLAT978_ALERT_2_G Number C-C Bonds with Positive Residual Density. 1 Info

---

0 **ALERT level A** = Most likely a serious problem - resolve or explain
0 **ALERT level B** = A potentially serious problem, consider carefully
7 **ALERT level C** = Check. Ensure it is not caused by an omission or oversight
11 **ALERT level G** = General information/check it is not something unexpected

3 ALERT type 1 CIF construction/syntax error, inconsistent or missing data
4 ALERT type 2 Indicator that the structure model may be wrong or deficient
8 ALERT type 3 Indicator that the structure quality may be low
2 ALERT type 4 Improvement, methodology, query or suggestion
1 ALERT type 5 Informative message, check

Datablock: mfsppm

Bond precision: C-C = 0.0039 A Wavelength=0.71073

Cell:	a=7.675(3)	b=7.829(3)	c=20.462(7)
	alpha=88.438(7)	beta=86.370(6)	gamma=79.737(7)
Temperature:	298 K		

	Calculated	Reported
Volume	1207.3(8)	1207.2(7)
Space group	P -1	P -1
Hall group	-P 1	-P 1
Moiety formula	C15 H15 N O2, C13 H14 N2	C15 H15 N O2, C13 H14 N2
Sum formula	C28 H29 N3 O2	C28 H29 N3 O2
Mr	439.54	439.54
Dx,g cm-3	1.209	1.209
Z	2	2
Mu (mm-1)	0.077	0.077
F000	468.0	468.0
F000’	468.18	
h,k,lmax	8,8,22	8,8,22
Nref	3481	3481
Tmin,Tmax	0.982,0.989	0.739,0.996
Tmin’	0.982	

Correction method= # Reported T Limits: Tmin=0.739 Tmax=0.996
AbsCorr = MULTI-SCAN

Data completeness= 1.000 Theta(max)= 23.279

R(reflections)= 0.0480(1861) wR2(reflections)= 0.1165(3421)

S = 0.888 Npar= 301

The following ALERTS were generated. Each ALERT has the format
test-name_ALERT_alert-type_alert-level.
Click on the hyperlinks for more details of the test.

Alert level B

```
THETM01_ALERT_3_B  The value of sine(theta_max)/wavelength is less than 0.575
            Calculated sin(theta_max)/wavelength =    0.5561
PLAT023_ALERT_3_B Resolution (too) Low [sin(theta)/Lambda < 0.6]..       0.56 Ang-1
```

Alert level C

```
PLAT230_ALERT_2_C Hirshfeld Test Diff for    C1       --C8       .         5.5 s.u.
PLAT241_ALERT_2_C High   'MainMol' Ueq as Compared to Neighbors of         C27 Check
PLAT242_ALERT_2_C Low    'MainMol' Ueq as Compared to Neighbors of         C21 Check
PLAT242_ALERT_2_C Low    'MainMol' Ueq as Compared to Neighbors of          C3 Check
PLAT906_ALERT_3_C Large K Value in the Analysis of Variance ......       2.184 Check
PLAT911_ALERT_3_C Missing FCF Refl Between Thmin & STh/L=    0.556          57 Report
```

Alert level G

```
PLAT007_ALERT_5_G Number of Unrefined Donor-H Atoms ..............           2 Report
PLAT883_ALERT_1_G No Info/Value for _atom_sites_solution_primary .     Please Do !
PLAT910_ALERT_3_G Missing # of FCF Reflection(s) Below Theta(Min).           3 Note
PLAT933_ALERT_2_G Number of OMIT Records in Embedded .res File ...           3 Note
PLAT941_ALERT_3_G Average HKL Measurement Multiplicity ...........         1.5 Low
PLAT978_ALERT_2_G Number C-C Bonds with Positive Residual Density.           0 Info
PLAT992_ALERT_5_G Repd & Actual _reflns_number_gt Values Differ by           2 Check
```

0 **ALERT level A** = Most likely a serious problem - resolve or explain
2 **ALERT level B** = A potentially serious problem, consider carefully
6 **ALERT level C** = Check. Ensure it is not caused by an omission or oversight
7 **ALERT level G** = General information/check it is not something unexpected

1 ALERT type 1 CIF construction/syntax error, inconsistent or missing data
6 ALERT type 2 Indicator that the structure model may be wrong or deficient
6 ALERT type 3 Indicator that the structure quality may be low
0 ALERT type 4 Improvement, methodology, query or suggestion
2 ALERT type 5 Informative message, check

Datablock: mf2mpm

Bond precision: C-C = 0.0066 A Wavelength=0.71073

Cell:	a=7.954(3)	b=8.027(3)	c=15.822(5)
	alpha=83.883(7)	beta=85.659(7)	gamma=66.931(7)
Temperature:	298 K		

	Calculated	Reported
Volume	923.5(6)	923.5(6)
Space group	P -1	P -1
Hall group	-P 1	-P 1
Moiety formula	C15 H14 N O2, C5 H7 N2, H2 O	C15 H14 N O2, C5 H7 N2, H2 O
Sum formula	C20 H23 N3 O3	C20 H23 N3 O3
Mr	353.41	353.41
Dx,g cm-3	1.271	1.271
Z	2	2
Mu (mm-1)	0.087	0.087
F000	376.0	376.0
F000'	376.16	
h,k,lmax	9,9,18	9,9,18
Nref	3271	3216
Tmin,Tmax	0.978,0.984	0.776,0.992
Tmin'	0.978	

Correction method= # Reported T Limits: Tmin=0.776 Tmax=0.992
AbsCorr = MULTI-SCAN

Data completeness= 0.983 Theta(max)= 24.987

R(reflections)= 0.0928(2106) wR2(reflections)= 0.2163(3216)

S = 1.091 Npar= 245

The following ALERTS were generated. Each ALERT has the format
test-name_ALERT_alert-type_alert-level.
Click on the hyperlinks for more details of the test.

Alert level C

```
PLAT230_ALERT_2_C Hirshfeld Test Diff for    C12      --C17      .        6.8 s.u.
PLAT230_ALERT_2_C Hirshfeld Test Diff for    C17      --C19      .        5.2 s.u.
PLAT241_ALERT_2_C High   'MainMol' Ueq as Compared to Neighbors of         N1 Check
PLAT340_ALERT_3_C Low Bond Precision on  C-C Bonds ...............    0.00663 Ang.
PLAT906_ALERT_3_C Large K Value in the Analysis of Variance ......     34.156 Check
PLAT906_ALERT_3_C Large K Value in the Analysis of Variance ......      5.684 Check
PLAT906_ALERT_3_C Large K Value in the Analysis of Variance ......      2.798 Check
PLAT911_ALERT_3_C Missing FCF Refl Between Thmin & STh/L=    0.594         54 Report
```

Alert level G

```
PLAT007_ALERT_5_G Number of Unrefined Donor-H Atoms ..............          4 Report
PLAT154_ALERT_1_G The s.u.'s on the Cell Angles are Equal ..(Note)      0.007 Degree
PLAT883_ALERT_1_G No Info/Value for _atom_sites_solution_primary .     Please Do !
PLAT909_ALERT_3_G Percentage of I>2sig(I) Data at Theta(Max) Still        32% Note
PLAT910_ALERT_3_G Missing # of FCF Reflection(s) Below Theta(Min).          1 Note
PLAT933_ALERT_2_G Number of OMIT Records in Embedded .res File ...          1 Note
PLAT941_ALERT_3_G Average HKL Measurement Multiplicity ...........        1.5 Low
PLAT978_ALERT_2_G Number C-C Bonds with Positive Residual Density.          0 Info
PLAT992_ALERT_5_G Repd & Actual _reflns_number_gt Values Differ by          3 Check
```

0 **ALERT level A** = Most likely a serious problem - resolve or explain
0 **ALERT level B** = A potentially serious problem, consider carefully
8 **ALERT level C** = Check. Ensure it is not caused by an omission or oversight
9 **ALERT level G** = General information/check it is not something unexpected

2 ALERT type 1 CIF construction/syntax error, inconsistent or missing data
5 ALERT type 2 Indicator that the structure model may be wrong or deficient
8 ALERT type 3 Indicator that the structure quality may be low
0 ALERT type 4 Improvement, methodology, query or suggestion
2 ALERT type 5 Informative message, check

Datablock: m3a6_0m_a_a

Bond precision: C-C = 0.0028 A Wavelength=0.71073

Cell:	a=6.0177(5)	b=11.1933(9)	c=13.7316(13)
	alpha=79.420(4)	beta=78.913(4)	gamma=75.134(3)
Temperature:	300 K		

	Calculated	Reported
Volume	868.45(13)	868.45(13)
Space group	P -1	P -1
Hall group	-P 1	-P 1
Moiety formula	C15 H15 N O2, C5 H6 N2	C15 H15 N O2, C5 H6 N2
Sum formula	C20 H21 N3 O2	C20 H21 N3 O2
Mr	335.40	335.40
Dx,g cm-3	1.283	1.283
Z	2	2
Mu (mm-1)	0.084	0.084
F000	356.0	356.0
F000’	356.14	
h,k,lmax	7,13,16	7,13,16
Nref	3059	3055
Tmin,Tmax	0.978,0.987	0.867,0.987
Tmin’	0.978	

Correction method= # Reported T Limits: Tmin=0.867 Tmax=0.987
AbsCorr = MULTI-SCAN

Data completeness= 0.999 Theta(max)= 24.997

R(reflections)= 0.0517(2551) wR2(reflections)= 0.1471(3055)

S = 1.083 Npar= 230

The following ALERTS were generated. Each ALERT has the format
test-name_ALERT_alert-type_alert-level.
Click on the hyperlinks for more details of the test.

Alert level C

```
PLAT420_ALERT_2_C D-H Bond Without Acceptor  N2       --H2B      .     Please Check
PLAT906_ALERT_3_C Large K Value in the Analysis of Variance ......      2.503 Check
PLAT911_ALERT_3_C Missing FCF Refl Between Thmin & STh/L=    0.595          4 Report
```

Alert level G

```
PLAT007_ALERT_5_G Number of Unrefined Donor-H Atoms .............           4 Report
PLAT883_ALERT_1_G No Info/Value for _atom_sites_solution_primary .     Please Do !
PLAT909_ALERT_3_G Percentage of I>2sig(I) Data at Theta(Max) Still       58% Note
PLAT910_ALERT_3_G Missing # of FCF Reflection(s) Below Theta(Min).         1 Note
PLAT941_ALERT_3_G Average HKL Measurement Multiplicity ..........        3.1 Low
PLAT978_ALERT_2_G Number C-C Bonds with Positive Residual Density.         0 Info
```

0 **ALERT level A** = Most likely a serious problem - resolve or explain
0 **ALERT level B** = A potentially serious problem, consider carefully
3 **ALERT level C** = Check. Ensure it is not caused by an omission or oversight
6 **ALERT level G** = General information/check it is not something unexpected

1 ALERT type 1 CIF construction/syntax error, inconsistent or missing data
2 ALERT type 2 Indicator that the structure model may be wrong or deficient
5 ALERT type 3 Indicator that the structure quality may be low
0 ALERT type 4 Improvement, methodology, query or suggestion
1 ALERT type 5 Informative message, check

Datablock: mi9m

Bond precision: C-C = 0.0047 A Wavelength=0.71073

Cell:	a=7.785(2)	b=8.359(2)	c=28.691(8)
	alpha=90	beta=95.470(6)	gamma=90
Temperature:	298 K		

	Calculated	Reported
Volume	1858.6(8)	1858.5(9)
Space group	P 21/n	P 1 21/n 1
Hall group	-P 2yn	-P 2yn
Moiety formula	C15 H14 N O2, C5 H7 N2, H2 O	C15 H14 N O2, C5 H7 N2, H2 O
Sum formula	C20 H23 N3 O3	C20 H23 N3 O3
Mr	353.41	353.41
Dx,g cm-3	1.263	1.263
Z	4	4
Mu (mm-1)	0.086	0.086
F000	752.0	752.0
F000’	752.32	
h,k,lmax	9,9,34	9,9,33
Nref	3269	3262
Tmin,Tmax	0.981,0.986	0.697,0.988
Tmin’	0.978	
