# Supplementary material for: Multicomponent Solids of Niflumic and Mefenamic Acids Based on Acid-Pyridine Synthon
Source: Front Chem. 2022 Mar 31;10:729608. doi: 10.3389/fchem.2022.729608 (PMC9009247; doi:10.3389/fchem.2022.729608)
Supplement: Supplementary file 3 [file DataSheet2.docx]

“For Table of contents only”

# Multicomponent solids of niflumic and mefenamic acid based on acid-pyridine synthon

Vineet Kumar, ^†^ Pramod Kumar Goswami, ^†^ Balendra, ^†^ Shailabh Tewari, ^†^ and Arunachalam Ramanan^* †^

^†^Department of Chemistry, Indian Institute of Technology Delhi, HauzKhas, New Delhi-110016, India


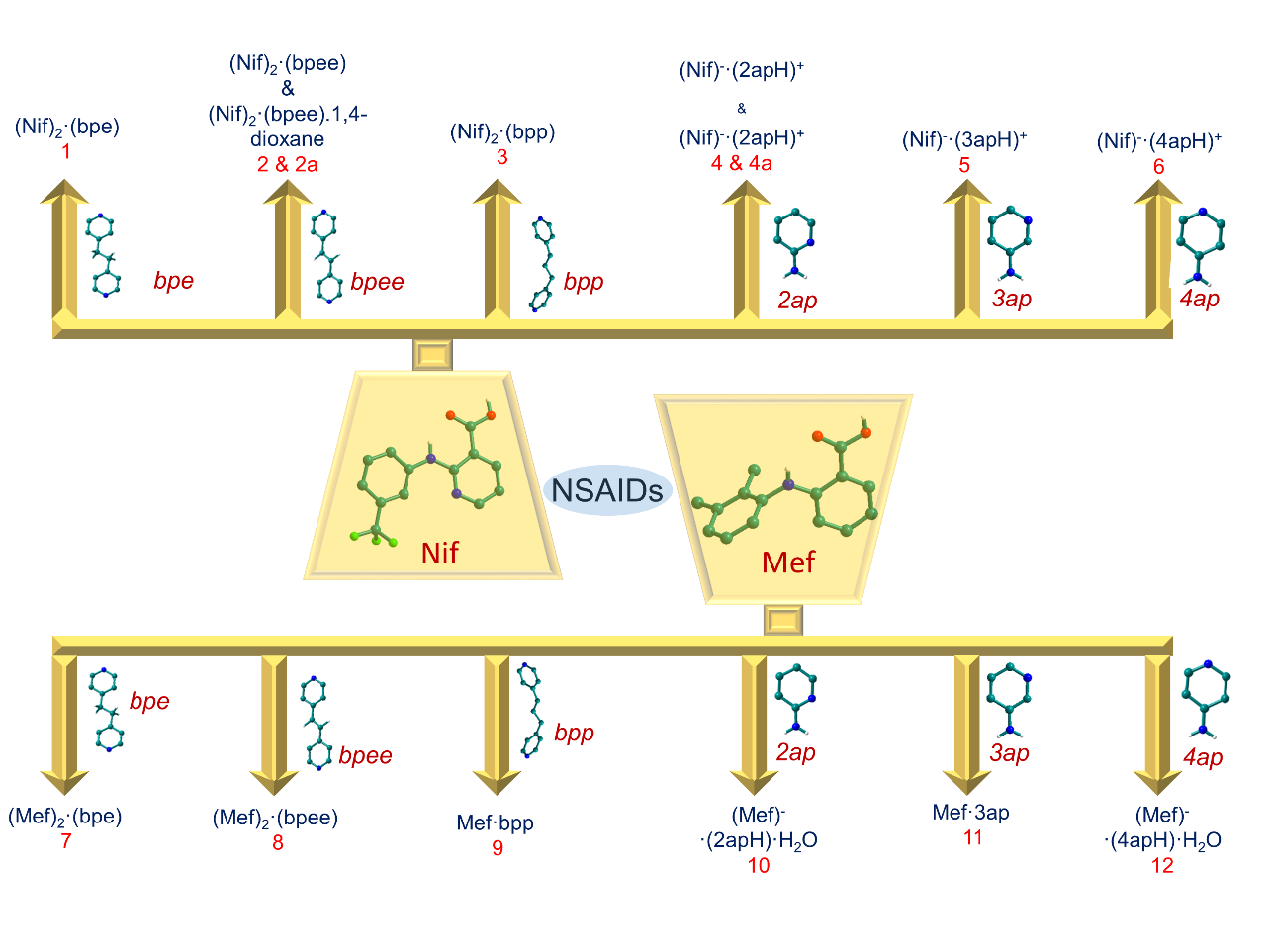
E-mail: [aramanan@chemistry.iitd.ac.in](mailto:aramanan@chemistry.iitd.ac.in) (A.R.), Tel: +91-11-26591507. Fax: +91-11-26581102.
